# Honey environmental DNA reveals entomological fingerprints through dual mitochondrial cytochrome c oxidase subunit 1 (COI) and cytochrome b (CYTB) metabarcoding

**DOI:** 10.1038/s41598-026-46493-y

**Published:** 2026-04-04

**Authors:** Anisa Ribani, Samuele Bovo, Valeria Taurisano, Kate Elise Nelson Johnson, Ayça Özkan Koca, Giuseppina Schiavo, Valerio Joe Utzeri, Francesca Bertolini, Luca Fontanesi

**Affiliations:** 1 Animal and Food Genomics Group, Division of Animal Sciences, Department of Agricultural and Food Sciences, University of Bologna, Viale Giuseppe Fanin 46, 40127 Bologna, Italy; 2 Department of Gastronomy and Culinary Arts, Maltepe University, 34857, Maltepe, Istanbul, Turkey

**Keywords:** Apiculture, Beekeeping, DNA fingerprint, eDNA, Hemiptera, Honeydew, Ecology, Ecology, Plant sciences, Zoology

## Abstract

Honey carries environmental DNA (eDNA) from organisms encountered by honey bees and can therefore encode an “entomological fingerprint” of agricultural and forest landscapes. We compared two metabarcoding assays targeting mitochondrial regions (cytochrome c oxidase subunit 1, COI; and cytochrome b, CYTB) to profile plant-sucking insects (Hemiptera) in honey samples from Italy and Türkiye, including citrus blossom, polyfloral, and honeydew honey samples. We designed a new short CYTB primer pair optimised for Aphididae and evaluated it alongside our previously published Hemiptera-COI assay. Across 3.51 million reads, our pipeline assigned 1.25 million COI and 1.48 million CYTB reads to Neoptera. CYTB resolved more taxa per sample (mean 7.9 families, 36 species) than COI (4.5 families, 11.7 species) and yielded a higher proportion of species-level assignments (98.3% vs. 43.7%). COI captured *Metcalfa pruinosa* (Flatidae) where abundant—often dominating polyfloral honey samples—whereas CYTB revealed fine-scale diversity of aphids relevant to citrus orchards and conifer/oak forests. Short-amplicon haplotype screening recovered multiple COI haplotypes for *M. pruinosa*, *Thelaxes suberi*, *Cinara cedri* and *Aphis gossypii*, suggesting potential for population monitoring from honey eDNA. Our results show that combining complementary metabarcodes mitigates primer and database biases and enhances landscape-scale inference, honey authentication, and surveillance of invasive or pest hemipterans.

**Supplementary Information:**

The online version contains supplementary material available at 10.1038/s41598-026-46493-y.

## Introduction

Honey is a unique traditional food product derived from insects, particularly honey bees. It is produced through enzymatic transformation by honey bees and subsequent biochemical maturation of carbohydrate-rich exudates of plant origin. Honey is classified into two main types based on its origin: (i) blossom or floral honey, which comes mainly from floral nectar, and (ii) honeydew honey, which is derived mainly from the secretions of certain trees or the excretions of Hemiptera insects that feed on epigean plant parts^1,2^. This food matrix is valued not only for its nutritional properties and sweet taste but also for the diversity of its flavour profiles, which depend on its origin, and in turn by its chemical composition^2–4^.

A minor chemical component of honey is DNA, derived from various organisms present in the ecological context in which honey is produced, including the honey bees that produced it and the organisms of the hive’s micro- and macro-ecosystems^5–17^. This environmental DNA (eDNA) enables fingerprinting the honey, providing direct or indirect insights into its biological, environmental, and geographical origin^10–20^. For example, targeted analyses of honey bee DNA have been used to identify the honey bee species and subspecies that produced it^12,21,22^. Additionally, these analyses have been utilised to investigate the distribution of *Apis mellifera* mitochondrial DNA lineages based on the geographic origin of honey samples and infer the subspecies that produced it^12,19–22^. Honey eDNA has also been employed to track the presence and spread of honey bee pathogens, parasites and pests^13,14,16,17^. End point PCR analyses and metabarcoding analyses, based on next generation sequencing techniques, have been used for these purposes^13,14,16,17^. Plant DNA metabarcoding of honey has overcome the constraints of earlier Sanger sequencing methods. This approach utilises universal plant barcode regions to decode complex botanical signatures and uncover the foraging preferences of honey bees, thereby supporting honey authentication and ecological research (e.g.^5–7,10,23–25^. The use of multiple barcoding systems has expanded the range of botanical profiles that can be identified, addressing some of the limitations associated with using only one barcode^6,23–27^. Assessment of biodiversity using metabarcoding approaches face well-known challenges: barcode coverage and informativeness; primer bias and efficiency; and incomplete or mis-annotated reference databases^28,29^.

Using a metabarcoding approach, we also investigated the entomological fingerprint derived from honeydew traces present in all honey samples regardless of their prevalent components having a nectar origin^18^. The barcode was based on a short fragment of the mitochondrial cytochrome c oxidase subunit 1 (COI or COX1) gene, which was amplified with specifically designed primers targeting insects of the order Hemiptera^18^. Hemipterans include all major plant-sucking insects that produce honeydew, which is then fed on by honey bees. The planthopper insect, *Metcalfa pruinosa*, was the most represented hemipteran in most honey samples from this study^18^, with other minor representatives from the Aphididae family, frequently being overshadowed by the overrepresentation of *M. pruinosa*. To gain a more complete understanding of the hemipterans parasitising plants in agricultural and forest landscapes, it is necessary to improve the barcoding system by targeting species that may go undetected using the previously designed primers. This is essential to address limitations of single targets that plant metabarcoding has partially solved by utilising multiple barcodes targeting different informative genomic regions^23–25,28,29^.

In this study, to enhance the barcoding potential for understanding the entomological fingerprint present in honey, we designed and tested a new primer pair that specifically targets the mitochondrial cytochrome b (CYTB) gene of the Aphididae family and closely related taxa. We then compared the results obtained with these new primers to those obtained using the system targeting the hemipteran COI mitochondrial DNA by analysing various honey samples from different geographical and botanical origins. In doing so, we developed a multiple entomological metabarcoding system that can provide enhanced insights into plant-sucking insects that inhabit the agricultural and forest landscapes visited by honey bees and that can be described from honey eDNA.

## Methods

### Honey samples

This study utilised 25 honey samples. Twelve honey samples were produced in 2021 in the province of Cosenza, located in the Calabria region (South of Italy), with each sample originating from a different apiary. The honey samples were monofloral, specifically sourced from the nectar of citrus fruit tree flowers. These honey samples derived from all colonies within the respective apiaries (ranging from 24 to 72 colonies). Eight polyfloral honey samples were collected in 2022, each from a honeycomb of a different colony, within three distinct apiaries situated in the province of Bologna, in the Emilia-Romagna region (North of Italy). Additionally, five honey samples were from Türkiye: two were polyfloral and were produced in 2022 (from the western part of the Black Sea region and the Eastern region of Anatolia, respectively); three were produced in 2021 in different apiaries, each situated in forests of pines, cedars or oaks. Further details regarding these honey samples can be found in Table 1.

**Table 1:** Honey samples utilised in the study.

Honey sample ID	Geographic origin	Geographic coordinates of the apiary^1^	Botanical origin	Year of production	Months of production
HC_1	Cosenza (Italy)	39.8170, 16.4801	Citrus tree	2021	May
HC_2	Cosenza (Italy)	39.3945, 16.2818	Citrus tree	2021	May
HC_3	Cosenza (Italy)	39.6808, 16.4355	Citrus tree	2021	May
HC_4	Cosenza (Italy)	39.6404, 16.5152	Citrus tree	2021	May
HC_5	Cosenza (Italy)	39.7661, 16.3950	Citrus tree	2021	May
HC_6	Cosenza (Italy)	39.7117, 16.4467	Citrus tree	2021	May
HC_7	Cosenza (Italy)	39.7687, 16.3864	Citrus tree	2021	May
HC_8	Cosenza (Italy)	39.4127, 16.2951	Citrus tree	2021	May
HC_9	Cosenza (Italy)	39.4241, 16.3052	Citrus tree	2021	May
HC_10	Cosenza (Italy)	39.6932, 16.4185	Citrus tree	2021	May
HC_11	Cosenza (Italy)	39.4228, 16.2651	Citrus tree	2021	May
HC_12	Cosenza (Italy)	39.4014, 16.2425	Citrus tree	2021	May
HB_1A1	Bologna (Italy)	44.5171, 11.4877	Polyfloral	2022	June
HB_1A2	Bologna (Italy)	44.5171, 11.4877	Polyfloral	2022	June
HB_1C1	Bologna (Italy)	44.5171, 11.4877	Polyfloral	2022	June
HB_2A2	Bologna (Italy)	44.4336, 11.4335	Polyfloral	2022	June
HB_2B2	Bologna (Italy)	44.4336, 11.4335	Polyfloral	2022	June
HB_2C2	Bologna (Italy)	44.4336, 11.4335	Polyfloral	2022	June
HB_3A3	Bologna (Italy)	44.4611, 11.3296	Polyfloral	2022	June
HB_3B3	Bologna (Italy)	44.4611, 11.3296	Polyfloral	2022	June
HT_1	Bolu-Centre (Türkiye)	40.7329, 31.6074	Polyfloral	2022	June-August
HT_3	Kars-Centre (Türkiye)	40.6012, 43.0969	Polyfloral	2022	June-August
HT_13	İzmir-Kemalpaşa (Türkiye)	38.4279, 27.4189	Pine forest honeydew	2021	September-October
HT_19	Isparta-Şarlıkaraağaç-Çarıksaraylar (Türkiye)	38.1210, 31.4188	Cedar forest honeydew	2021	June - July
HT_28	Çanakkale-Çan-Söğütalan (Türkiye)	39.9011, 26.9173	Oak forest honeydew	2021	July - August

^1^Latitude and longitude.

### DNA extraction from honey samples

DNA extraction from honey was carried out following the procedures described in previous studies^11,12^. Briefly, 50 g of honey was divided into four aliquots, each of 12.5 g, and were placed in 4 different Falcon tubes. Then, 40 mL of ultrapure water was added to each tube and vortexed for 10 s followed by an incubation at 40 °C for 1 min. This wash-incubation cycle was repeated ten times. The Falcon tubes were then centrifuged for 25 min at 5,000 g at room temperature. The supernatant was discarded, and the pellet was resuspended in 5 mL of ultrapure water. The contents of the four Falcon tubes were combined and further diluted with ultrapure water to reach 45 mL. The combined Falcon tube was centrifuged for 25 min at 5,000 g at room temperature and the supernatant was discarded. The pellet was resuspended in 0.5 mL of ultrapure water and transferred to a 1.5 mL tube, which was stored at -20 °C for subsequent DNA extraction. This process began with the addition of one mL of CTAB extraction buffer [2% (w/v) cetyltrimethylammoniumbromide; 1.4 M NaCl; 100 mM Tris-HCl; 20 mM EDTA pH 8.0; 5 µL of RNase A solution (10 mg/mL) pre-incubated for 10 min at 60 °C] along with 30 µL of proteinase K (20 mg/mL) to the pelleted honey material. The mixture was then incubated at 65 °C for 90 min with gentle mixing. After incubation, samples were cooled to room temperature and centrifuged for 10 min at 16,000 g. Next, 700 µL of supernatant was transferred to a tube containing 500 µL of chloroform/isoamyl alcohol (24:1) and vortexed for 30 s. The mixture was then centrifuged for 15 min at 16,000 g at room temperature. The supernatant was carefully transferred to a new 1.5 mL tube. DNA was precipitated in two steps: first with 500 µL of isopropanol, then with 500 µL of ethanol/water 70:30 (v/v). The resulting pellets were rehydrated with 30 µL of sterile water and stored at -20 °C until PCR analyses.

### Insect samples and DNA extraction from these specimens

Several insect species were sampled for this study. Specimens included *Apis mellifera ligustica* (n. 3), collected from a hive in the Emilia Romagna region (North of Italy), *Apis mellifera carnica* (n. 3), collected from a hive in Friuli-Venezia Giulia region (North-East of Italy), and *Apis mellifera mellifera* (n. 3), collected from a hive in the Liguria region (North-West of Italy). Additional samples were from various species of the order Hemiptera: *Metcalfa pruinosa* (n. 3), *Issus muscaeformis* (n. 3), and *Lyristes plebejus* (n. 3) from the suborder Auchenorrhyncha; *Halyomorpha halys* (n. 3), and *Corythucha ciliata* (n. 3) from the suborder Heteroptera; *Aphis craccivora* (n. 3), *Cinara cedri* (n. 3), *Cinara cupressi* (n. 3), *Cinara pectinata* (n. 3), *Toumeyella parvicornis* (n. 3), *Icerya purchasi* (n. 3), and *Myzus persicae* (n. 3) from the suborder Sternorrhyncha. All samples were collected in the Emilia-Romagna region (North of Italy). DNA extraction from the legs of these samples was performed using the Wizard Genomic DNA Purification Kit (Promega) following the manufacturer’s instructions for animal tissues. These species were utilised to test PCR amplification with a new primer pair that was designed in this study (see below).

### Design of PCR primers targeting the mitochondrial CYTB gene in aphids

Since the DNA contained in honey is highly degraded^10–21,30–32^, it was necessary to design universal primers that could amplify a short but informative amplicon. Therefore, all entries containing Aphididae mitochondrial cytochrome b (CYTB) gene sequences were retrieved from GenBank/EBI (November 2024). The alignment of these CYTB sequences was obtained using the MUltiple Sequence Comparison by Log-Expectation (MUSCLE) tool (http://www.ebi.ac.uk/Tools/msa/muscle/). Using the aligned sequences, PCR primers (forward: 5’-CCATGAGGWCAAATATCATTTT-3’; reverse: 5’-CCTCCTCAAATTCAAATWAC-3’) were selected in the most conserved regions to amplify a 101 bp fragment across the Hemiptera order. Supplementary material 1 Figure S1 shows the alignment of the corresponding sequence region from a few representative aphid species of different subfamilies. Datasets showing the alignments of the forward and reverse primers with hemipteran sequence entries retrieved from GenBank/EBI (November 2024) obtained using BLASTN (limited to 5000 entries) have been deposited in Zenodo.

### PCR for the CYTB and COI mitochondrial DNA gene targets

PCR was performed separately with two primer pairs: the pair described above that targets the hemipteran mitochondrial CYTB gene and another pair that targets the hemipteran mitochondrial COI gene, which was reported in our previous study (forward: 5’-TGGAWCAGGAACAGGATGAAC-3’; reverse: 5’-AAATGAARTTGATTGCTCCTA-3’^18^. Datasets showing the alignments of the forward and reverse COI primers with hemipteran sequence entries retrieved from GenBank/EBI (November 2024) obtained with BLASTN (limited to 5000 entries) have been deposited in Zenodo. These datasets update information reported in our previous study^18^.

PCR amplifications were carried out utilising a 2720 Thermal Cycler (Thermo Fisher Scientific, Waltham, MA, USA)) in total volume of 20 µL. The reaction mixture contained 2X Kapa HiFi HotStart ReadyMix PCR kit (Hoffmann-La Roche, Basel, Switzerland), 10 pmol of each primer and approx. 50 ng of template DNA. PCR amplification consisted of an initial denaturation at 95 °C for 5 min, followed by 35 cycles of 95 °C for 30 s, annealing for 30 s at the temperatures specified below, and 72 °C for 30 s, with a final extension at 72 °C for 5 min. PCR products were visualised after 2.5% agarose gel electrophoresis in TBE 1X buffer and 1X GelRed Nucleic Acid Gel Stain (Biotium Inc., Hayward, CA, USA). Every PCR analysis was performed with two replicates per sample, positive controls (two replicates using as template an equimolar mix of DNA extracted from the insect species listed above) and a negative control (a tube with no DNA template).

To determine the optimal annealing temperature for the CYTB primer pair for amplifying DNA from Hemiptera species isolated from honey, which also contains DNA of *Apis mellifera* that constitutes the most represented DNA of entomological origin in honey^12,15,18,20,21^, PCR analyses were conducted using a temperature range of 52–64 °C. The objective was to identify the temperature at which Hemiptera DNA would amplify preferentially over *A. mellifera* DNA. Amplification reactions were performed with DNA from the sampled Hemiptera and *A. mellifera* specimens. This empirical analysis revealed that 57 °C was the temperature that could potentially maximise amplification from Hemiptera species with reduced efficiency against honey bee DNA. The annealing temperature for the COI primer pair was set at 58 °C, as previously optimised using the gradient-based approach^18^. To validate that the newly designed primers successfully amplified the expected CYTB sequence, amplicons obtained from DNA of various insect species were sequenced using the BrightDye Terminator Cycle Sequencing Kit (NIMAGEN, Nijmegen, the Netherlands) and a capillary sequencer (ABI3100 Avant, Thermo Fisher Scientific,). Amplicons obtained with the two primer pairs from the honey DNA samples were utilised for next generation sequencing as described below.

### Next generation sequencing

Next generation sequencing (NGS) was carried out using Ion GeneStudio S5 System (Thermo Fisher Scientific) following the manufacturer’s instructions. Briefly, PCR products obtained using the two Hemiptera-specific primers were equimolarly pooled for each honey sample and then purified with ExoSAP-IT (Thermo Fisher Scientific). A total of 25 libraries were produced by end-repair and ligation of the DNA fragments with a specific barcode using the Ion Plus Fragment Library and Ion Xpress Barcode Adapters kits (Thermo Fisher Scientific). Each library was then quantified with qPCR using the Ion Library TaqMan™ Quantitation Kit and QuantStudio 7 Real Time instrument (Thermo Fisher Scientific) and processed for template preparation. The template preparation was completely automated by Ion Chef instrument (Thermo Fisher Scientific) and the protocol included the following steps: emulsion PCR, enrichment and sequencing chip loading steps using the Ion 510™ & Ion 520™ & Ion 530™ Chef kit (Thermo Fisher Scientific). A 520 Ion chip was loaded in the GeneStudio Ion S5 sequencer (Thermo Fisher Scientific) setting a 200 bp General Sequencing.

### Sequence data analysis

*Database development* **–** Marker specific COI and CYTB databases were constructed as follows: (1) for each marker, nucleotide sequences presenting the marker name in the FASTA header were retrieved from the NCBI nucleotide (nt) resource^33^; (2) sequences were screened for the presence of both forward and reverse primer sequences by using the *blastn* tool from the BLAST + v.2.7.1 + suite^34^. A minimum sequence identity threshold of 75% was used to consider a match valid; (3) only sequences presenting both primer regions were retained; (4) sequences were trimmed to retain only the region between the primers; (5) only sequences associated with a NCBI taxonomy identifier (taxid) assigned to the Neoptera group (taxid: 33340) were included in the final database(s). The COI database comprised 262,605 sequences [representing 53,270 taxids distributed over different taxonomic levels and 43,793 groups (a group is defined as a set of sequences sharing a sequence identity of 100%)], whereas the CYTB database comprised 8,768 sequences (no. of taxids = 1,670; no. of groups = 1,971). The two databases shared 699 taxids. To identify groups, sequences were clustered using CD-HIT v4.7^35^, which performed pairwise alignment of each sequence against all others.

*Next Generation Sequencing data processing* **–** Barcoded samples were demultiplexed using the Ion Torrent Suite v5.18.2. For each barcode, reads corresponding to the two amplicons were separated using Cutadapt v1.2^36^. Error tolerance was set to 20%. Primer sequences were simultaneously removed during this step. Only reads containing both the forward and reverse primer sequences were retained and then inspected with FastQC v.0.11.8 (http://www.bioinformatics.babraham.ac.uk/projects/fastqc/). To obtain high-quality datasets, PRINSEQ Lite v.0.20.4^37^ was then used to remove (i) nucleotides at the 5’- and 3’- ends presenting a quality score Q < 20, and (ii) reads with an average Q < 20.

*Taxonomic annotation of reads* **–** Taxonomic assignment followed (with some adjustments) the workflow reported by Bovo et al.^10,15^. Briefly, reads were aligned over the two databases with *blastn*. For each read, we retained all the alignments presenting the following statistics identical to the top alignment (i.e. the one with the lowest E-value): (i) read length (≥ 20 bp) (ii) sequence coverage (≥ 95%), (iii) sequence identity (≥ 95%) and (iv) E-value (< 0.01). Then, taxonomic assignment followed the Lowest Common Ancestor (LCA) approach, as implemented in Python 2.7 via in-house developed scripts. To avoid background noise, taxa with ≥ 0.5% of assignments based on total number of reads after filtering were selected. For graphical representation of species frequencies, percentages of reads assigned to genera and species were shown. The total number of families and species detected for each marker in each honey sample was counted and the averages per marker were calculated. Two-tailed paired t-tests were applied to evaluate potential differences between families and groups across markers.

*Mitochondrial haplotype analysis from NGS data* **–** The presence of mitochondrial haplotypes (also referred as mitotypes) was evaluated by analysing sequences obtained from four Hemiptera species for the COI region (*Metcalfa pruinosa*, *Thelaxes suberi*, *Cinara cedri* and *Aphis gossypii*) and two for CYTB region (*Cinara cedri* and *Myzus persicae*), selected according to the abundance in term of number of reads obtained across samples. Mitotypes were retrieved from the in-house curated databases as representative unique sequences, derived from NCBI nucleotide database. Annotated reads specific to the organism under consideration (after the LCA assignment) were then compared with the reference sequences and assigned to a given haplotype only if they showed 100% sequence identity to NCBI entries already reported by others and/or described in the literature. A specific mitotype was called among these reads if it was detected in at least two different honey samples or if its number of reads was > 0.05% of all considered reads for the corresponding hemipters. All analyses were performed in Python 3.2. Phylogenetic analysis of *A. gossypii* haplotypes was obtained through the construction of a neighbour-joining (NJ) tree applying 1000 bootstrap replicates using MEGA software v11^38^. The evolutionary distances were computed using the Maximum Composite Likelihood method, while the rate variation among sites was modeled with a gamma distribution (shape parameter = 1)^39^.

## Results

### Amplifications with the COI and CYTB barcodes and summary statistics of sequencing data

DNA extracted from all honey samples investigated was successfully amplified for the targeted COI and CYTB mitochondrial regions. As previously mentioned, CYTB PCR reactions were conducted at an annealing temperature of 57 °C, based on the optimal efficiency of end-point PCR using Hemiptera DNA as a template rather than *Apis mellifera* DNA. Specifically, *A. mellifera* DNA was amplified at an annealing temperature of 52 °C, but no detectable amplicons were visible on gel-electrophoresis at higher temperatures. Similarly, *Metcalfa pruinosa* DNA produced products at 52 °C, with no products observed at higher temperatures. In contrast, the DNA of almost all other hemipteran species yielded PCR products at an annealing temperature of 57 °C, while at 64 °C only some of them successfully amplified the expected product (Supplementary material 1 Table S1).

Ion GeneStudio S5 sequencing generated a total of 3,509,235 reads. After filtering, 1,641,071 COI reads and 1,868,057 CYTB reads were obtained from all the 25 honey samples analysed. Following the stringent bioinformatics pipelines we set up, a total of 1,247,610 and 1,482,524 sequences were assigned to Neoptera taxa for COI and CYTB, respectively, accounting for 76.02% and 79.36% of assigned reads in relation to filtered reads (Table 2).

**Table 2:** General statistics on the sequenced reads for the COI and CYTB barcodes of the analysed honey samples.

COI									CYTB							
Honey ID	No. of reads sequenced	No. of reads after filtering	Reads assigned to families^1^	% of annotated reads	No. of families	Reads assigned to species (%)	% reads assigned to species	No. of species	No. of reads sequenced	No. of reads after filtering	Reads assigned to families ^1^	% of annotated reads	No. families	Reads assigned to species (%)	% reads assigned to species	No. species
HC_1	30,906	30,906	17,533	56.73%	1	1384	7.89%	12	131,879	131,872	124,156	91.00%	13	121,374	97.75%	67
HC_2	56,349	56,349	40,077	71.12%	2	1376	3.43%	10	30,409	30,409	28,569	87.13%	4	28,001	98.01%	28
HC_3	14,740	14,740	10,693	72.54%	2	531	4.97%	10	25,132	25,132	23,703	88.03%	6	23,555	99.37%	27
HC_4	166,682	166,676	126,496	75.89%	5	12,897	10.20%	21	116,945	116,939	108,202	87.01%	9	105,482	97.48%	57
HC_5	89,602	89,599	68,371	76.31%	3	2738	4.00%	12	124,109	124,106	115,599	85.33%	10	114,319	98.89%	47
HC_6	52,056	52,055	30,251	58.11%	1	1796	5.94%	9	163,568	163,567	156,317	88.01%	2	154,364	98.75%	21
HC_7	129,731	129,729	99,841	76.96%	7	9965	9.98%	19	175,443	175,434	157,566	82.02%	7	157,164	99.74%	48
HC_8	58,409	58,408	42,617	72.96%	1	1419	3.33%	8	163,548	163,534	149,422	84.57%	8	147,187	98.50%	45
HC_9	56,761	56,757	24,957	43.97%	3	1383	5.54%	6	97,842	97,839	86,305	86.86%	8	85,814	99.43%	41
HC_10	71,478	71,478	62,273	87.12%	2	5377	8.63%	9	59,831	59,828	56,057	80.73%	7	55,922	99.76%	33
HC_11	77,652	77,650	55,298	71.21%	4	4835	8.74%	12	146,955	146,949	133,212	85.24%	8	132,523	99.48%	43
HC_12	81,793	81,793	59,525	72.78%	3	3803	6.39%	11	55,711	55,711	49,700	83.33%	4	48,739	98.07%	35
HB_1A1	43,170	43,168	18,874	43.72%	4	12,183	64.55%	10	16,545	16,545	14,215	86.00%	6	14,054	98.87%	20
HB_1A2	88,050	88,048	80,650	91.60%	4	59,612	73.91%	10	54,379	54,377	46,463	87.00%	5	46,332	99.72%	17
HB_1C1	1482	1481	662	44.70%	5	633	95.62%	10	15,599	15,597	9339	60.90%	3	5878	62.94%	19
HB_2A2	37,192	37,192	35,242	94.76%	10	30,457	86.42%	17	85,229	85,228	13,416	16.70%	14	12,890	96.08%	41
HB_2B2	18,359	18,358	16,345	89.03%	5	13,822	84.56%	10	51,855	51,854	4415	9.00%	14	3861	87.45%	40
HB_2C2	82,268	82,267	70,490	85.68%	6	58,985	83.68%	12	19,764	19,763	3960	27.10%	9	3501	88.41%	23
HB_3A3	80,549	80,549	73,599	91.37%	8	54,483	74.03%	11	52,847	52,839	24,491	60.30%	13	24,282	99.15%	35
HB_3B3	72,008	72,006	62,106	86.25%	7	41,355	66.59%	11	55,393	55,392	9274	20.40%	10	7251	78.19%	45
HT_1	23,777	23,775	851	3.58%	6	845	99.29%	11	18,754	18,753	8550	52.50%	10	7847	91.78%	40
HT_3	33,634	33,634	27,656	82.23%	4	2649	9.58%	15	48,663	48,663	45,517	93.60%	4	45,080	99.04%	25
HT_13	70,508	70,507	37,995	53.89%	6	37,918	99.80%	9	50,829	50,826	33,656	66.60%	6	32,663	97.05%	29
HT_19	87,725	87,725	81,678	93.11%	5	81,660	99.98%	14	70,629	70,626	63,635	90.60%	8	62,255	97.83%	46
HT_28	116,221	116,221	103,530	89.08%	9	103,525	100.00%	13	36,275	36,274	16,785	47.10%	10	16,547	98.58%	28
**Total/ Average** ^2^	**1,641,102**	**1,641,071**	**1,247,610**	**76.02%**	A 4.52 *	**545,631**	**43.73%**	A 11.68*	**1,868,133**	**1,868,057**	**1,482,524**	**79.36%**	A 7.92*	**1,456,885**	**98.27%**	A 36*

^1^ Excluding *A. mellifera.* ^2^ "A" indicates averages; other boxes report totals. *: The asterisk indicates statistical significance (p<0.001) of differences among averages of the number of families and species detected per marker.

### Overview of the COI-based entomological fingerprinting of honey samples

**Fig. 1:**
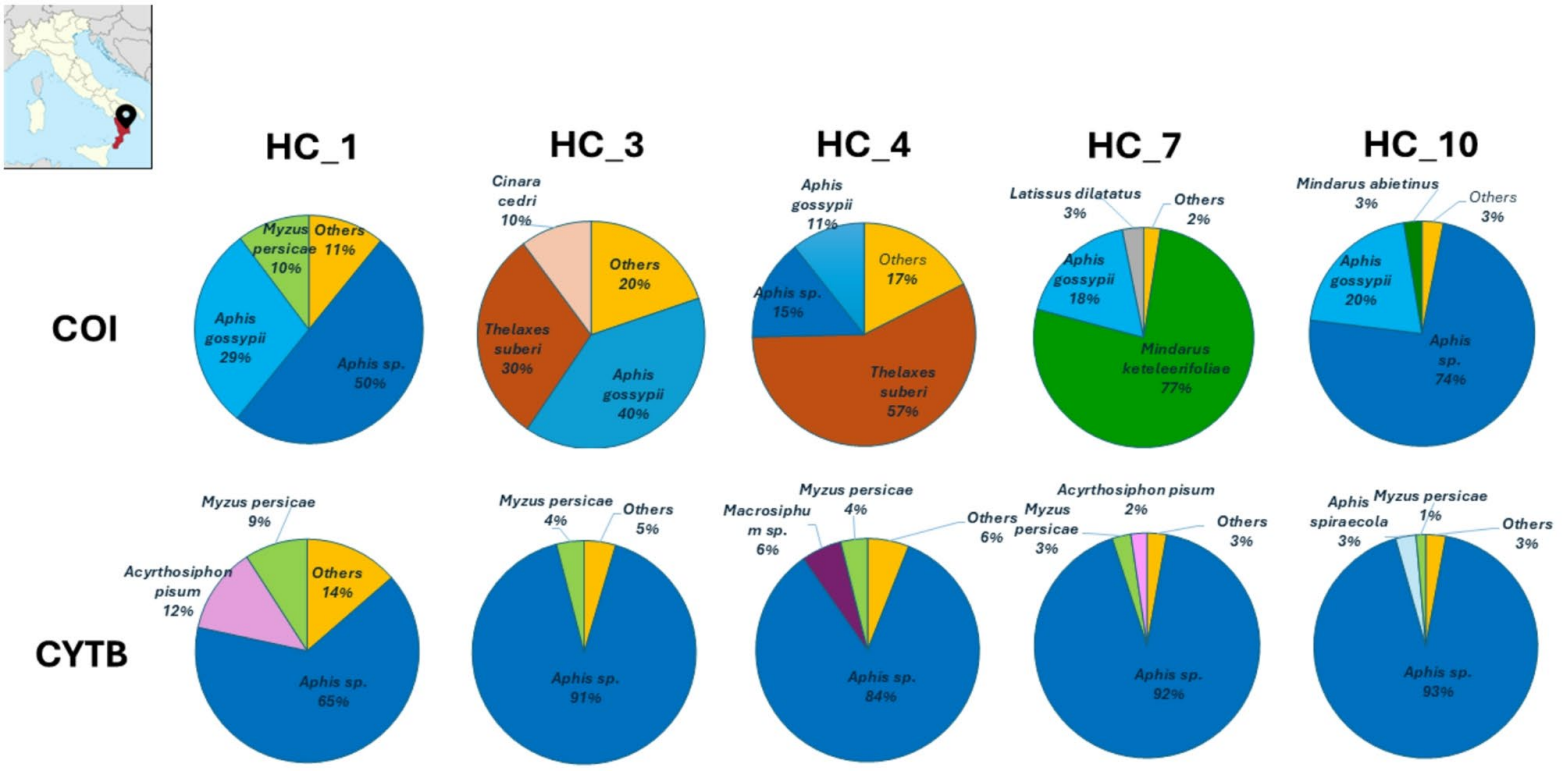
Proportion of COI and CYTB reads assigned to species for five representative citrus tree honey samples from Calabria region (Italy).

The most relevant insect species detected across samples produced from the three geographic regions considered (Calabria and Emilia-Romagna regions in Italy and Türkiye) are reported in Table 3. When considering the citrus tree honey samples from the Calabria region (Italy), the COI marker detected an average of 2.83 families and 11.58 species (including genera, species and varieties taxa) per sample. A summary of the assigned reads for a few representative citrus tree honey samples is shown in Fig. 1. Additional details for all samples can be found in Supplementary material 1 Table S2. Aphididae was the most frequently detected family (> 90% of frequency), mainly represented by the *Aphis* genus. Within the *Aphis* genus, *A. gossypii*, a polyphagous species that affects citrus among other plants, was found in every honey sample, with a frequency ranging from approximately 10% to 86% among the detected species. Other species belonging to the Aphididae family, such as *Brachycaudus helichrysi* (commonly known as leaf curl plum aphid or leaf-curling plum aphid, a pest of *Prunus* species), *Cinara cedri* (feeding on cedars, from which its common name of cedar bark aphid derives), and *Myzus persicae* (a highly damaging polyphagous and cosmopolitan aphid species), among others, were also detected with some reads in various samples. In a few honey samples, other families were detected, such as Thelaxidae, represented by the *Thelaxes suberi* species (feeding on *Quercus* *sp.*) and Mindaridae represented by the *Mindarus abetinus* species, mainly feeding on *Abies sp.* trees. These two species were the most prevalent for three honey samples.

**Fig. 2:**
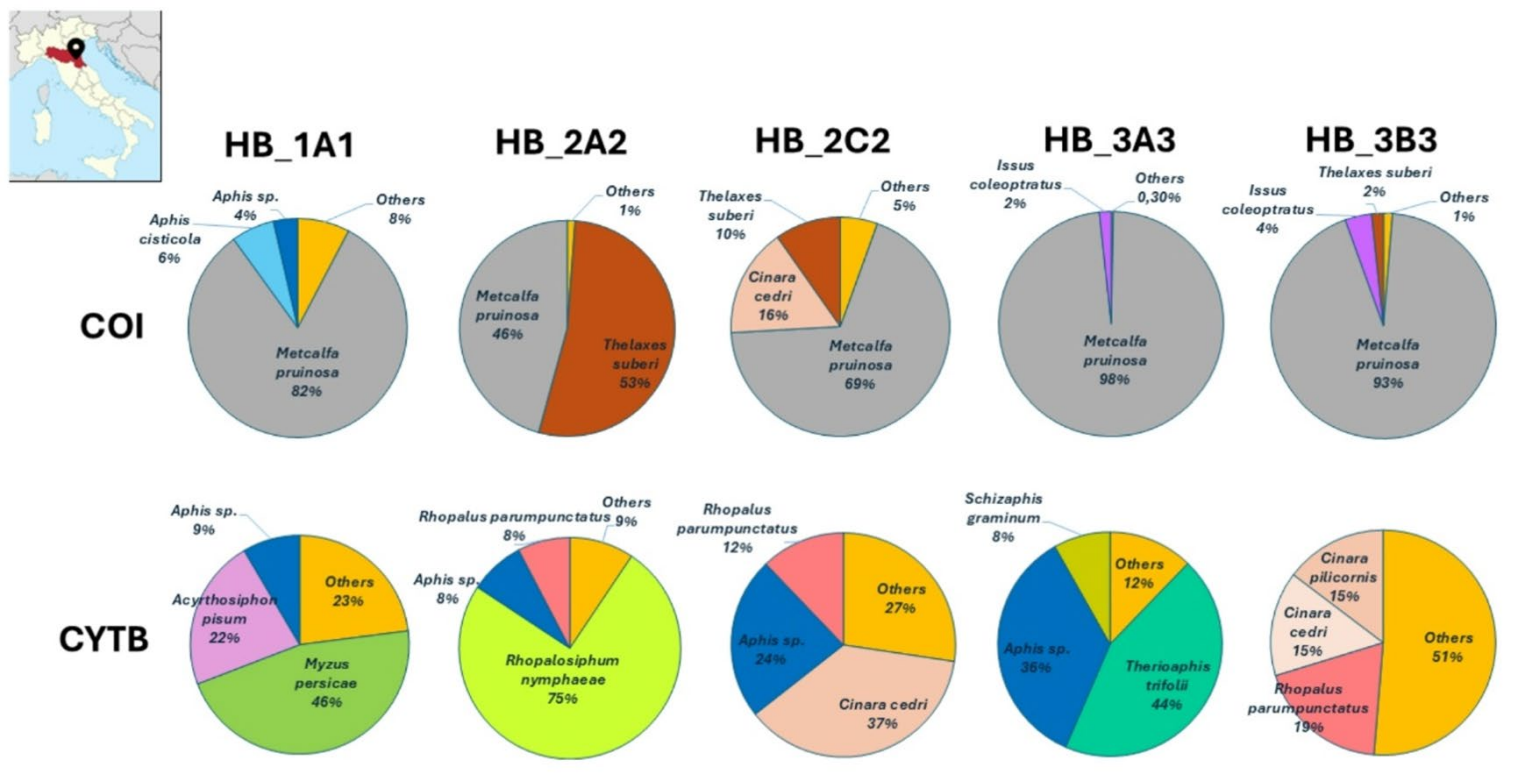
Proportion of COI and CYTB reads assigned to species for five representative polyfloral honey samples from Emilia-Romagna region (Italy).

The COI-based fingerprint of the polyfloral honey samples from the Emilia-Romagna region (Northern Italy) reflected the plant-sucking insect populations expected in the environmental context in which they were produced. Figure 2 summarises the assigned reads for a few representative honey samples from Emilia-Romagna. Supplementary material 1 Table S2 reports detailed information for all honey samples produced in this region. On average, 6.13 families and 11.38 species (including genera, species and varieties taxa) were identified per honey sample. Reads from the polyphagous hemipter *Metcalfa pruinosa* belonging to the Flatidae family were the most numerous in six out of eight honey samples, ranging from 66.3% to 99.8%. In the other two samples, *M. pruinosa* was the second most common species in terms of the number of reads (13.6% in HB_1C1 and 45.7% in HB_2A2), with *Protaphis (Aphis) anuraphoides* (common on plants of the Asteraceae family) and *Thelaxes suberi* (an oak aphid) being the most represented hemipters (82.2% and 53.1% of reads). Other species, including *Cinara cedri*, *Aphis cisticola* (that typically feeds on various rock rose species, namely from the genus *Cistus*) and *Issus coleoptratus* (a planthopper belonging to the family Issidae, feeding on the phloem of different trees) were identified in various samples with relevant numbers of reads. In addition, in a few samples, a small number of reads were assigned to insects from orders other than Hemiptera (Supplementary material 1 Table S2). For example, the coleopteran *Oryzaephilus surinamensis* was found in samples HB_1C1, HB_2A2, HB_2B2, HB_2C2 and HB_3B3 (with assigned reads ranging from 0.79% to 0.007%). Another coleopteran, *Meloe mediterraneus*, was identified in samples HB_2A2 (0.14%) and HB_2C2 (0.02%). Lepidopteran species *Paraperithous gnathaulax*, *Niphonyx segregate*, *Mompha sp.* and *Odonestis pruni* were found in sample HB_3A3 (with reads ranging from 0.01% to 0.002%).

**Fig. 3:**
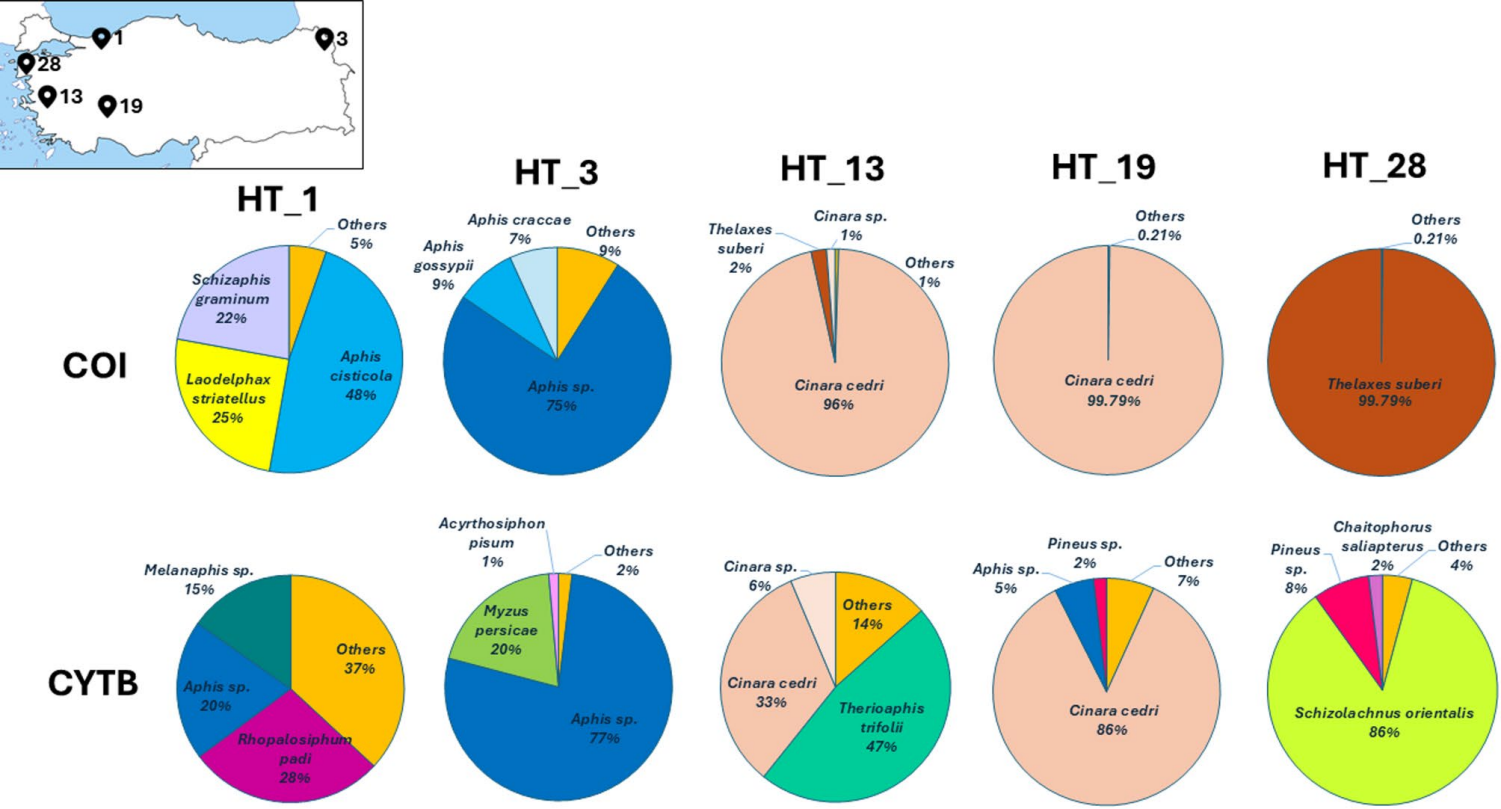
Proportion of COI and CYTB reads assigned to species for honey samples from Turkey.

The COI metabarcoding analysis of Turkish honey samples identified an average of 6 families and 12.4 species per sample. Figure 3 provides information on the read profiles of selected Turkish honey samples, while Supplementary material 1 Table S2 contains comprehensive data on the reads assigned to different insect species for all analysed honey samples from this area. One polyfloral honey sample (HT_1) exhibited a unique profile including *Aphis cisticola* (comprising approx. 47% of assigned reads), an aphid that primarily feeds on *Cistus sp.* plants. Additionally, *Laodelphax striatellus*, also known as the small brown planthopper (which primarily feeds on cereals), accounted for around 25% of reads, and *Schizaphis graminum*, also known as wheat aphid (which feeds on crops and other wild Poaceae plants), represented 22% of reads. The honey samples from pine forest (HT_13) and cedar forest (HT_19) predominantly showed *Cinara cedri*, making up ~ 96% and ~ 99% of reads, respectively. This aphid is known to feed on various conifers including *Pinus* and *Cedrus* trees. In the oak forest honey (HT_28), the most prevalent insect was *Thelaxes suberi* (> 99% of reads), an oak aphid. Also, in this group of Turkish honey samples, a small number of reads were assigned to insects belonging to orders other than Hemiptera. For example, a few coleopteran species were identified in samples HT_1 (*Cranophorus sp.*; 0.12% of annotated reads), HT_3 (*Meloe sp.*; 0.19%), HT_13 (*Oryzaephilus surinamensis*; 0.008%; *Cranophorus sp.*; 0.003%), HT_19 (*Meloe sp.*; 0.017%) and HT_28 (*Scymnus subvillosus*, a common aphidophagous species in the Middle East and in the Mediterranean region; 0.012%; *Diaprepes abbreviatus*; 0.001%). Additionally, the pyralid *Cadra figulilella* (0.002%) and the Cecidomyiidae *Cecidomyia pini* (0.001%) were identified in HT_28.

**Table 3:** List of the most prevalent insect species in terms of number of reads detected with the COI and CYTB barcodes across samples produced from the three geographic regions considered.

Region	COI	CYTB
Calabria (Italy)	*Aphis sp.*,* Aphis gossypii*,* Thelaxes suberi*	*Aphis sp.*,* Myzus persicae*,* Acyrthosiphon pisum*
Emilia-Romagna (Italy)	*Metcalfa pruinosa*,* Thelaxes suberi*,* Cinara cedri*	*Myzus persicae*,* Therioaphis trifolii*,* Rhopalosiphum nymphaeae*
Türkiye	*Aphis sp.*,* Cinara cedri*,* Thelaxes suberi*	*Aphis sp.*,* Cinara cedri*,* Schizolachnus orientalis*

### Overview of the CYTB-based entomological fingerprinting of honey samples

The most relevant insect species detected across samples produced from the three geographic regions considered (Calabria and Emilia-Romagna regions in Italy and Türkiye) are reported in Table 3. The metabarcoding analysis of the citrus honey samples from the Calabria region based on the CYTB gene detected an average of 7.17 insect families and 41 species. The obtained profiles were similar across samples. Figure 1 summarises the metabarcoding patterns for a few honey samples produced in this region. Information on the profiles obtained in all 12 analysed samples is reported in Supplementary material 1 Table S3. In all samples, most reads (ranging from ~ 63% to ~ 93%) were assigned to the taxa *Aphis sp. Myzus persicae*, a polyphagous aphid common in citrus orchards, was consistently among the top three to four species in terms of the number of reads, after *Aphis sp.. Aphis spiraecola*, also known as the green citrus aphid, was ranked among the other top 10 most represented species in 10 out of 12 analysed honey samples. *Macrosiphum sp.*, commonly found on citrus trees, was detected in all honey samples, and held a top 10 position in 10 samples. *Aphis gossypii*, another cosmopolitan and polyphagous aphid that feeds on citrus trees, was present in 10 of the honey samples. Other aphid species, including *Acyrthosiphon pisum* (that primarily feeds on plants of the family Fabaceae), *Hyalopterus amygdali* (the mealy almond aphid that feeds primarily on almond and apricot trees, as well as other *Prunus* species) and *Cinara formosana* (reported on various species of pine) provided a signature of the Mediterranean environment typical of the Calabria region.

The CYTB-based metabarcoding of polyfloral honey samples from the Emilia-Romagna region (Italy) identified an average of 9.25 families and 30 species. Figure 2 describes the entomological profiles for a few representative honey samples from this region, while Supplementary material 1 Table S3 ranks all matched species for all honey samples produced in Emilia-Romagna. The entomological profiles included *Myzus persicae*,* Acyrthosiphon pisum*, *Rhopalus parumpunctatus* (a bug typically found in dry grassland and associated with *Cerastium sp.* plant), *Therioaphis trifolii* (also known as the spotted alfalfa aphid or yellow clover aphid, a pest of legumes), *Cinara cedri* and *Rhopalosiphum nymphaeae* (commonly known as the water lily aphid, which feeds on various *Prunus* species) as the top species in terms of the number of reads detected in one or two of these honey samples. Most of these aphids were also frequently present in various honey samples where they were not the first species. The profiles also included other well represented species, such as *Schizaphis graminum*, also known as wheat aphid (which feeds predominantly on plants of the Poaceae family), *Cinara tujafilina*, commonly known as the cypress pine aphid or thuja aphid (an oligophagous species that primarily feeds on plants of the Cupressaceae family), *Cinara pilicornis*, also known as the spruce shoot aphid or brown spruce shoot aphid (a European native aphid that predominantly feeds on *Picea* *sp.*), and several other aphids. It was also interesting to note that in two honey samples (HB_2A2 and HB_2B2), reads were assigned to *Propylea japonica*, a coleopter of the family Coccinellidae, that represents a natural predator of various aphid species.

The analysis of the five Turkish honey samples based on the CYTB barcode identified an average of 7.6 families and 33.6 species per sample. Figure 3 summarises the proportion of assigned reads to various insect species for these samples, while Supplementary material 1 Table S3 includes their complete entomological profiles ranked according to the number of assigned reads. In the two polyfloral honey samples (HT_1 and HT_3), a heterogeneous entomological profile was observed, including both specialised and polyphagous aphids. In HT_1, the highest number of reads were assigned to *Rhopalosiphum padi* (bird cherry – oat aphid), a pest of several cereals, followed by *Aphis sp.* and other oligophagous or more specialised aphids that feed on groups of grasses or trees, such as *Melanaphis sp.* (primarily feeding on Rosaceae and Poaceae), *Chromaphis juglandicola* (attacking walnuts) and *Chaitophorus saliapterus* (specialised on *Salix* species), among others. In HT_3, the entomological profile included polyphagous and oligophagous species: the top taxa were *Aphis sp.*, followed by *Myzus persicae* and other aphids primarily feeding on legumes. The entomological profiles of the pine and cedar forest honeydew honey samples (HT_13 and HT_19) included *Cinara cedri* among the top species, with other aphids feeding on various legumes with a relevant number of reads (e.g., *Therioaphis trifolii*, *Acyrthosiphon pisum*). The highest number of reads obtained for the oak forest honeydew honey was assigned to *Schizolachnus orientalis*, followed by *Pineus sp.*, both primarily feeding on Pinaceae trees. This honey sample also returned reads (0.53%) assigned to the coleopteran predator of aphids, *Propylea japonica*. Notably, in all samples barcoded with the CYTB region, we identified reads assigned to various lepidoptera species (Supplementary material 1 Table S3). The proportion of these reads was lower than 0.1% in the Calabria and Emilia-Romagna honey samples, but higher than 0.1% in the Turkish samples. The most represented species in terms of the proportion of assigned reads were the pyralid *Plodia interpunctella* (0.69% of the assigned reads) and the noctuid *Mythimna separata* (0.39%) in HT_1 and the noctuid *Noctua janthe* (0.34%) in HT_19.

### Comparative analysis between the two barcoding systems

Generally, the newly designed CYTB-based barcoding identified more taxa in terms of the average number of families and species (including genera, species and subspecies) per honey sample than the COI-based barcoding system. Specifically, CYTB detected an average of 7.88 families compared to the 4.52 identified by COI (paired t-test: *p* = 2.0E-4) and 36 species versus the 11.8 detected by COI (*p* = 5.0E-8). The higher number of families detected with CYTB was primarily due to differences in the honey samples produced in Calabria (Supplementary material 1 Table S4). The higher number of species detected with the CYTB-based barcoding compared to the COI-based system was evident in the honey samples produced in all three regions (Supplementary material 1 Table S4). When these average numbers were compared across regions within markers, there were no differences except for the lower number of COI-detected families in the Calabria region (Supplementary material 1 Table S5). Additionally, most CYTB reads were assigned to genera and species (an average of 98.27% per sample) while only an average of 43.73% of COI sequences were assigned to species. Furthermore, approximately 56% of COI reads detected taxa corresponding to family, subfamily and tribe levels (Table 2). The two barcoding systems partially overlapped in terms of identified entomological profiles, especially for a few citrus honey samples (from the Calabria region) and the Turkish *Cedrus* honeydew honey sample. This last sample reported the same top two taxa for both markers, including *Cinara cedri*, which is a clear indicator of the *Cedrus* forest environment. For most other samples, the profiles obtained with the two barcodes were largely complementary, with only a small number of species appearing in different positions based on the number of assigned reads. An interesting observation is that the CYTB barcoding system never detected *Metcalfa pruinosa* or any other members of the family Flatidae. This outcome was expected because the new primer pair used for CYTB barcoding did not amplify *M. pruinosa* DNA in the end point-PCR analysis conducted for primer validation. In honey samples where *M. pruinosa* was absent or nearly absent (e.g. Calabrian samples), the number of COI reads assigned to species or genera was limited (about 10%), further supporting the higher resolution power of the CYTB barcoding system in detecting species in this context.

Finally, both barcoding markers detected several non-Hemiptera taxa, even if with a small number of assigned reads. These other taxa were primarily annotated as lepidopteran, coleopteran and dipteran species. Comparing the two systems, the CYTB metabarcoding analysis identified more species than the COI-based metabarcoding, prevalently from lepidopteran species (78 lepidopteran taxa and 2 coleopteran species). The COI system identified prevalently coleopteran (7 species), then 5 lepidopteran species, 1 dipteran and 1 hymenopteran species.

### Identification of mitochondrial haplotypes for some hemipter species using sequenced reads

The assignment of multiple reads to the same species within and across honey samples presents an opportunity to explore population genetic information for various insects in an unconventional way. By combining information from the literature and sequences deposited in NCBI for the two targeted mitochondrial genes, we aimed to identify different mitochondrial haplotypes (or mitotypes) from the same species by analysing sequenced reads from honey samples. In this analysis we focused on four hemipteran species for the COI barcode (*Metcalfa pruinosa*, *Thelaxes suberi*, *Cinara cedri* and *Aphis gossypii*; Supplementary material 1 Table S6) and two hemipteran species for the CYTB barcode (*Cinara cedri* and *Myzus persicae*; Supplementary material 1 Table S7). The criteria we used to assign a read to a haplotype was 100% sequence identity with what was already reported in the literature and/or deposited in NCBI, obtaining an indirect cross-validation of our results.

**Fig. 4:**
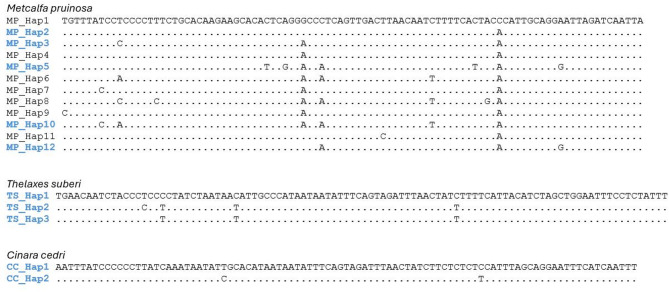
Sequence alignment of COI mitochondrial haplotypes found in *Metcalfa pruinosa*, *Thelaxes suberi* and *Cinara cedri*. Nucleotide positions identical with those of the first sequence are marked with a dot. Haplotypes detected in this study are reported in blue.

For *Metcalfa pruinosa*, we used a total of 995 COI sequence entries from our NCBI derived database to compile a list of 12 mitotypes (MP_Hap1-MP_Hap12) that could be distinguished within the short COI sequence amplified in this study (Fig. 4; Supplementary material Table S6). These haplotypes were also reported in our previous study^18^(Fig. 4). Other studies, based on a 470 bp gene sequence derived from *M. pruinosa* specimens collected in various countries, have reported a total of 20 mitochondrial COI haplotypes in this flatid^40,41^. The correspondence between the haplotypes identified in this study and those reported in previous studies is detailed in Supplementary Table S8^18,40,41^. Five out of the 12 compiled mitotypes, namely MP_Hap2, MP_Hap3, MP_Hap5, and MP_Hap10, were found in the eight honey samples from the Emilia-Romagna region, which were the only samples containing reads assigned to this planthopper (Supplementary material 1 Table S6). Our previous study^18^ only reported three of these haplotypes (see the correspondence in Supplementary material 1 Table S8). One sample analysed in the current study, HB_1A2 contained all five mitotypes, with four of them at minor frequencies (< 0.18%). The same three mitotypes (MP_Hap3, MP_Hap5 and MP_Hap12) were found in three samples (HB_1C1, HB_2C2 and HB_3A3), while two other samples (HB_2A2 and HB_3B3) contained two mitotypes (MP_Hap3 and MP_Hap12). MP_Hap3 was the most abundant mitotype in all eight Emilia-Romagna samples, with a fraction of reads ranging from 100% (in HB_1A1 and HB_2B2) to 97.8% (in HB_1C1). MP_Hap3 in our study corresponds to MPH01 from the study by Lee et al.^41^(Supplementary material 1 Table S8), which was also the most frequently reported in the Republic of Korea and several other countries, including Italy^40,41^. This was also the most abundant mitotype that we reported in our previous study^18^(indicated as Hap6; see Supplementary material 1 Table S8).

By considering the COI amplified region, we compiled three different mitotypes (TS_Hap1, TS_Hap2 and TS_Hap3) for *Thelaxes suberi* using nine sequence entries presented in our NCBI derived database (Fig. 4). All these mitotypes were found comparing reads obtained in seven honey samples (two from the Calabria region, three from the Emilia-Romagna region, and two from Türkiye; Supplementary material 1 Table S6). TS_Hap3 was the only one detected in three samples, the two from the Calabria region and one from the Emilia-Romagna region (HB_2B2). One honey sample (HB_2A2) from Emilia-Romagna and one from Türkiye contained all three mitotypes (with TS_Hap3 having > 99% of reads). Another sample from the Emilia-Romagna region contained TS_Hap2 (30.26%) and TS_Hap3 (69.74%) and another sample from Türkiye contained a peculiar profile with TS_Hap1 at high frequency (77.84%), together with TS_Hap2 (22.16%).

For *Cinara cedri*, we retrieved a total of 20 COI-gene sequence entries from our NCBI derived database and compiled only two mitochondrial haplotypes (indicated as CC_Hap1 and CC_Hap2) spanning the amplified COI region (Fig. 4). Both mitotypes were identified by comparing reads obtained from 10 honey samples (four from the Calabria region, four from the Emilia Romagna region and two from Türkiye; Supplementary material 1 Table S6). All Calabrian honey samples and one from Emilia-Romagna contained only haplotype CC_Hap2. The other three samples from Emilia-Romagna contained both haplotypes, with prevalence of CC_Hap1. The two Turkish samples contained only CC_Hap1.

**Fig. 5:**
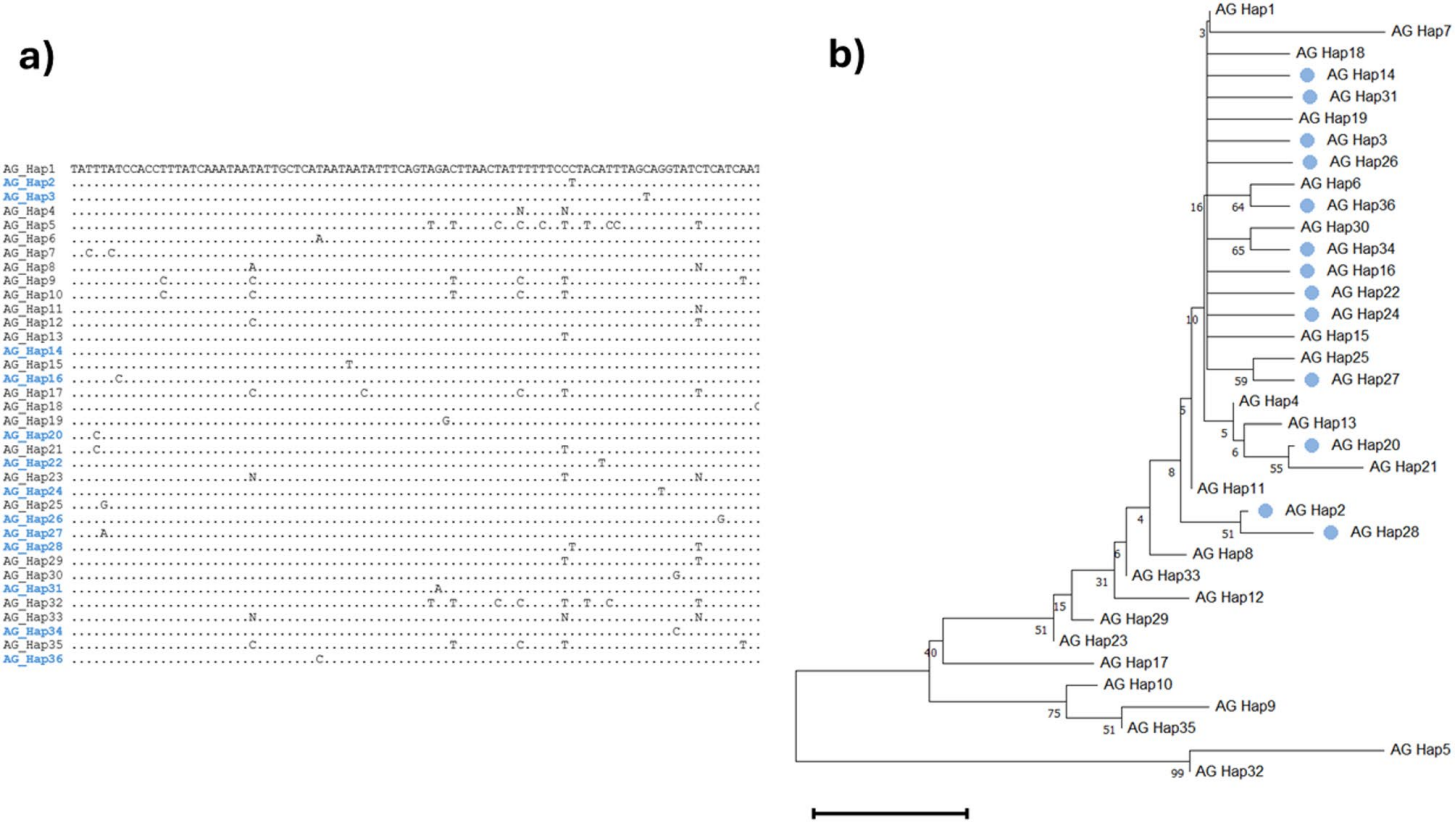
*Aphis gossypii* COI mitochondrial haplotypes. **(a)** Sequence alignment of *A. gossypii* COI mitochondrial haplotypes, including those identified in this study (highlighted in blue). Nucleotide positions identical with those of the first sequence are marked with a dot. **(b)** Neighbour-joining phylogenetic tree based on *A. gossypii* COI mitochondrial sequences, including those identified in this study (highlighted with blue dots). The percentage of replicate trees in which the associated taxa are clustered together in the bootstrap test (1000 replicates) are shown below the branches. The tree is drawn to scale, with branch lengths in the same units as those of the evolutionary distances used to infer the phylogenetic tree.

For *Aphis gossypii*, a total of 1232 COI sequences, annotated in their NCBI entries as being from this aphid species, were obtained providing information that differentiated 36 mitotypes distinguishable from the region amplified in this study (Fig. 5; Supplementary material 1 Table S6). Since a short gene region produced this large number of mitotypes, we evaluated their phylogenetic relationships to tentatively clarify this question. Figure 5 includes an unrooted NJ phylogenetic tree constructed with these short sequences to preliminarily explore this issue. Upon examining the topology of this tree, it was evident that approximately 50% of these haplotypes were quite divergent, forming two groups of haplotypes possibly constituting monophyletic clusters supported by high bootstrap values (first group: AG_Hap10, AG_Hap9, AG_Hap35; second group: AG_Hap5, AG_Hap32). This situation could be due by the presence of more than one *A. gossypii* like related cryptic species or subspecies. *Aphis gossypii* has already been indicated to be polyphyletic species by Coeur d’acier^42^, who reported three COI haplotypes based on the sequencing of a 658 bp region in four specimens. However, as the purpose of our study was not to clarify the phylogenetic relationships within this species (or correct any potential original assignment errors), we maintained the assignment to *A. gossypii* for all 36 mitotypes. Thirteen of these mitotypes were also identified in some of our honey samples derived from all groups. Two of them (AG_Hap2 and AG_Hap28) were clustered together separately from the core of the other haplotypes that were closely clustered, compared to the other block of more closely related haplotypes (Fig. 5; Supplementary material 1 Table S6). When considering all these 13 mitotypes, a very small number of reads were assigned to *A. gossypii* in two samples from Emilia-Romagna and two samples from Türkiye. More reads assigned to *A. gossypii* were obtained from all Calabrian honey samples, which included all 13 reported haplotypes (Supplementary material 1 Table S6). One sample, HC_4, contained 11 different mitotypes. The haplotypes with the largest number of reads were AG_Hap3 and AG_Hap24, which in the Calabrian honey samples ranged from 1.56% to 97.19% and from 1.08% to 97.82%, respectively. Therefore, from these results, it appears that several closely related mitotypes are present in various populations of this aphid species.

When considering the CYTB targeted region, we compiled two mitochondrial haplotypes for *Cinara cedri* and three mitochondrial haplotypes for *Myzus persicae* using nine and 10 sequence entries from our NCBI derived database, respectively (Supplementary material 1 Figure S2: Supplementary material 1 Table S7). All 11 honey samples that contained reads from *Cinara cedri*, carried only CC_CYTB_Hap1 (Supplementary material 1 Table S7). *Myzus persicae* reads were obtained from 21 honey samples, all of which reported the presence of only My_CYTB_Hap1 (Supplementary material 1 Table S7).

## Discussion

Honey is a unique source of eDNA, that accumulates in this food matrix from various organisms involved directly or indirectly in different production steps and the production ecosystem^10–13,22–27^. We have already demonstrated that honey can provide an entomological DNA fingerprint primarily originating from honeydew, produced by plant-sucking insects belonging to the Hemiptera order^18^. Honeydew is opportunistically used by honey bees to supplement their carbohydrate-rich diet mainly derived from nectars. In some cases, honeydew can also be the primary source of nutrients for honey bees, and some types of honey are also typically obtained mainly from this sweet source^2^. Honeydew contains the DNA of the insects that have produced it, which is not degraded during the honey bee transformation processes^18^.

The COI-barcoding system that we have previously developed has provided an initial overview of entomological profiles derived from honeydew found in honey samples from various regions and botanical prevalence, including forest honeydew honey samples^18^. However, a more comprehensive overview can be obtained addressing critical issues commonly associated with metabarcoding systems^43^. Two major challenges could be partially addressed by analysing samples using multiple barcoding systems. Firstly, metabarcoding approaches rely on an initial PCR step, which can introduce biases due to different priming preferences towards certain taxonomic groups. Using multiple primer pairs can help mitigate systematic biases in this regard. Secondly, the accuracy of assigning reads to specific taxa depends on the completeness of reference databases for the targeted region within the organism group being studied (insects in this case). Poor or incomplete representation in these databases can introduce biases, as evidenced by a significant portion of reads that cannot be assigned to any taxa. It is also important to note that while metabarcoding approaches may not be considered quantitative assays, the number of reads assigned to a specific taxon can give an approximate indication of the relative abundance of DNA present in samples derived from identified species^18,44^. This abundance could be related to the biological abundance of that species in a particular environmental area, aside from the potential biases mentioned above. In this context, as already proposed by others^45^, the concept of abundance could be expressed using a categorical classification (for instance: high abundance, medium abundance and low abundance) based on the relative proportion of reads over all assigned reads without any linear associations, if there are no strong biases in amplification preferences of the specific metabarcoding system. In our case, another challenge arises from the fact that the DNA present in honey is highly degraded^10–21,30–32^. Therefore, we had to use universal primers that could amplify short fragments. This issue may introduce limitations in the selection of useful primers and, in turn, in the informativity of the amplified regions. This could further enhance the risk of biases related to the two main challenges of all metabarcoding studies.

In this pilot study we analysed polyfloral, monofloral and honeydew honey samples produced in various regions, different environments and climatic zones (North of Italy, South of Italy and Türkiye) using two mitochondrial metabarcoding systems: the previously designed COI-based metabarcode and a newly designed CYTB-based metabarcode. The parallel use of these two systems contributed to partially compensating for the biases mentioned above and provided a comparative overview that enhanced the entomological fingerprints of the analysed honey samples.

The COI-based system was confirmed to efficiently capture *Metcalfa pruinosa* from samples containing DNA from this flatid. This was evident in most samples from Emilia-Romagna. *Metcalfa pruinosa*, also known as citrus flatid planthopper, is an invasive pest that was first detected in Italy in 1979^46^. It is known to be a significant source of honeydew, making it beneficial for beekeepers^47^, despite its damaging impact on agricultural, forest and urban landscapes^48^. It was also interesting to note that we did not identify *M. pruinosa* in citrus derived honey samples from Calabria, even though this pest is well known for attacking citrus trees^46,49,50^. This is likely due to the extensive measures taken to minimise the impact of this flatid on citrus orchards, suggesting that honey entomological DNA profiles may indicate effective pest control strategies. Additionally, in this study we slightly increased the number of analysed polyfloral honey samples (n. 10) with the COI-system compared to our previous study where only two samples had this heterogeneous botanical origin^18^. This further expanded the entomological profiles obtained from heterogeneous environments, indirectly defined by their declared complex botanical origins. Despite the potential heterogeneity in terms of botanical origins, which could also result in a highly diverse entomological profile, most samples showed a predominant hemipteran taxon that accounted for the majority of assigned reads. This may be due to a seasonal effect, as honey was produced during a specific period of the year, possibly when there was a particular hemipteran infestation in the agricultural or forest environments surrounding the apiaries where samples were produced. Longitudinal studies over the entire honey production seasons could investigate this hypothesis in more detail.

When we compared the CYTB-derived profiles of these honey samples, along with all other samples, we observed that the CYTB barcoding system captured a larger number of species. Additionally, a larger proportion of reads per sample obtained with the CYTB metabarcoding system was assigned at the species level compared to those with the COI metabarcoding system (with a mean of 36 species for the CYTB and 11.68 species for the COI metabarcoding systems per sample). Since both the COI and CYTB mitochondrial genes exhibit a very similar low evolutionary mutation rate in Metazoa, including the Hemiptera order^51–53^, the difference in discriminative power of these two genes used in this study could be attributed to the informativeness of the reference databases that we compiled, which could also be related to the small size of the amplified gene regions. Even if the number of COI sequences in the compiled reference database was larger compared to that of the CYTB database, it could be possible that the amplified COI region was not as informative as the CYTB region. The CYTB database may contain more sequences present in the environmental agricultural and forest landscapes that we investigated through the analysed honey samples. Several other studies have compared the COI and CYTB resolutive power in metabarcoding analyses of environmental samples, yielding discordant results depending on the taxa investigated^54–56^. When considering the Hemiptera order, the informativeness of the COI and CYTB databases is still incomplete as several taxa are poorly represented. In our specific study we also cannot exclude that the designed COI primers could introduce a larger bias in terms of the number of amplifiable hemipteran species than the CYTB primers. Other studies are needed to clarify this question.

The combined profiles produced by the COI and CYTB metabarcoding data showed partially overlapping entomological DNA fingerprints for the same samples. The extent of the overlaps, in terms of species and relative abundance of their reads, varied and was mainly sample specific, even though some samples shared the same geographic and botanical origin. Despite some specific differences, the citrus honey samples from the Calabria region clearly exhibited an entomological profile typical of a Mediterranean region. This profile included hemipteran species that are commonly found in citrus orchards and in close derived agricultural landscapes, where some aphid species are known to attack citrus trees and other Mediterranean plants, despite several species exhibiting polyphagous behaviour (such as *Aphis gossypii* and *Myzus persicae*). The samples produced from the Emilia-Romagna region mainly showed complementary profiles derived from the two metabarcoding systems, because of the high abundance of *Metcalfa pruinosa* in most samples for the COI system, as previously mentioned. In a few samples of this region, combining the information from the COI-metabarcoding, which identified a significant abundance of reads assigned to *Thelaxes suberi*, with the identification of *Cinara* species observed for both metabarcoding systems, we can infer the presence of oaks and conifers in the landscape where they were produced^57,58^. The pine, cedrous and oak forest honeydew samples produced in Türkiye showed complementary COI and CYTB profiles that pointed to their specific honeydew origin, with the high relative abundance of reads assigned to *Thelaxes suberi* and *Cinara* species, which are typically present in forests of this region^59,60^.

A fraction of the sequenced reads, immediately filtered out by our bioinformatic pipeline, were assigned to *Apis mellifera*, which does not belong to the Hemiptera order. The amplification of *A. mellifera* DNA, although at very low efficiency, according to our initial negative tests, may be due to the over-representation of its DNA compared to that of other insect species^18^. In addition to honey bee reads, the entomological profiles obtained also provided information on other insects not belonging to the Hemiptera order, detected with a small number of reads. This may be because the COI and CYTB primers may not be completely hemipteran-specific and could amplify DNA from a broader taxonomic group, albeit with lower efficiency or only for a small number of species. The identified lepidopteran, coleopteran and dipteran species further complete the entomological profiles of samples, providing additional information on the ecological interactions within the agricultural and forest landscapes where they were produced. Particularly, the identification of the aphid-eating ladybugs *Propylea japonica* and *Scymnus subvillosus* can offer insights into their feeding ecological systems. These predators may leave DNA traces on honeydew, produced by their predated aphids, and collected by honey bees. Since some coccinellids, like *Propylea japonica*, act as biological control agents for aphid infestations^61^, it would be intriguing to gather indirect information on their distribution and spread using honey eDNA containing these traces. To achieve this, more targeted primers could be developed. These could also be designed to detect invasive entomological species or specific plant pests, opening new opportunities for indirect monitoring approaches in agricultural and forest landscapes.

Sequenced reads assigned to the same insect species within and across honey samples provided the opportunity to explore population genetic information of some hemipteran species, as a by-product of the main aim of this study. Initially, various mitochondrial haplotypes were compiled using sequence information from published literature and entries available in NCBI for the two barcodes. This process allowed us to construct reference resources for four species based on the COI gene region (*Metcalfa pruinosa*, *Thelaxes suberi*, *Cinara cedri* and *Aphis gossypii*) and two species for the CYTB gene region (*Cinara cedri* and *Myzus persicae*) that could be utilised in other studies aiming to monitor population genetic structures of these hemipters. Previous studies have also reported mitochondrial COI haplotypes in some of these species. For example, when comparing haplotype information for *Metcalfa pruinosa* populations as reported by Park et al.^40^ and Lee et al.^41^, we confirmed the high prevalence of the same mitotype in Italy. Furthermore, in this study we were able to identify five *M. pruinosa* COI haplotypes, an increase from the previously reported two^18^. This suggests that extensive honey-derived entomological metabarcoding can be a valuable tool in monitoring the population genetic structures and dynamics of invasive hemipteran pests. Another study reported three and two COI haplotypes in *Thelaxes suberi* and *Cinara cedri*, respectively^42^. We reported the same numbers of haplotypes for these two species, in addition to estimations of their frequencies based on the level of abundance of assigned reads. There was also some evidence of specific geographic distributions of some of these haplotypes, based on the geographical origin of the honey samples where they were identified. *Aphis gossypii* represents a polytypic species, with a high number of COI haplotypes, as derived from our compiled haplotype database and reported from other studies that investigated variability in this mitochondrial gene of this aphid in various countries^42,62–66^. We confirmed the presence of a high number of COI haplotypes in *Aphis gossypii* (13 were identified in our samples), with heterogeneous “mitotype fingerprints” across samples, that could provide an additional level of information. The CYTB metabarcoding data produced in the analysed honey samples did not provide any different haplotypes. In all samples that contained reads assigned to *Cinara cedri* and *Myzus persicae*, only one mitotype was detected. This could be due to the lower informativity within-species of the targeted CYTB gene region and the fewer number of studies focused on this gene which are useful for evaluating variability compared to what is available for the COI gene. In fact, only two and three haplotypes were compiled in silico for the two species respectively. Therefore, it is evident that critical issues for the identification of haplotypes using data generated with metabarcoding systems are the completeness and correctness of the reference databases. This would provide the opportunity for an indirect cross-validation of information linking previous studies with newly generated data, reducing biases derived from sequencing errors.

## Conclusions

Honey environmental DNA accumulates all biological traces collected through the high environmental exploration capacity of honey bees. Targeted DNA metabarcoding of this source of biological information reveals different aspects that may describe the ecological landscapes of agricultural and forest production systems and the honey bee feeding behavior in these environments.

By utilising multiple metabarcoding systems, more thorough entomological fingerprints can be obtained from honey eDNA, compensating potential biases derived from single systems. These fingerprints can be utilised for various purposes. For example, entomological profiles are linked to the geographical and botanical origin of honey, making this DNA-derived picture useful for honey authentication. This can be achieved by utilising information across species and specific haplotype-derived patterns of targeted insects. Within-species genetic variability obtained from these entomological profiles can provide cost-effective population genetic information that would otherwise be difficult or almost impossible to obtain. Entomological profiles derived from honey eDNA can be valuable for monitoring invasive insect species, determining their presence in a specific environment and understanding their population dynamics, which could have significant implications for their control. Additionally, insights into the biology of certain insects and their ecological interactions can also be inferred. To achieve these goals, appropriate models need to be developed.

To gain a more comprehensive understanding of the advantages and disadvantages in terms of complementarity and unresolved biases, it is necessary to analyse more honey samples from various regions, seasons and production systems using these two entomological barcoding systems. Additionally, in order to broaden the scope of entomological information that can be obtained from this source of eDNA, it is important to test and evaluate other universal primers using different regions of the mitochondrial COI and CYTB genes, as well as other informative targets.

## Supplementary Information

Below is the link to the electronic supplementary material.

Supplementary Material 1

## Data Availability

The datasets generated during the current study showing alignments of CYTB and COI primers with Hemiptera sequences are available in Zenodo with the following DOI: https://doi.org/10.5281/zenodo.19183389 . The sequence datasets generated and analysed during the current study are available in the EMBL-EBI European Nucleotide Archive (ENA) repository http://www.ebi.ac.uk/ena , with the project accession number PRJEB102161.
