# Supplementary material for: Honey environmental DNA reveals entomological fingerprints through dual mitochondrial cytochrome c oxidase subunit 1 (COI) and cytochrome b (CYTB) metabarcoding
Source: Sci Rep. 2026 Apr 4;16:18207. doi: 10.1038/s41598-026-46493-y (PMC13261148; doi:10.1038/s41598-026-46493-y)
Supplement: Supplementary file 1 — Supplementary Material 1 [file 41598_2026_46493_MOESM1_ESM.pdf]

Supplementary material 1

Honey environmental DNA reveals entomological fingerprints through dual mitochondrial cytochrome c oxidase subunit 1 (COI) and cytochrome b (CYTB) metabarcoding

Anisa Ribani, Samuele Bovo, Valeria Taurisano, Kate Elise Nelson Johnson, Ayça Özkan Koca, Giuseppina Schiavo, Valerio Joe Utzeri, Francesca Bertolini, Luca Fontanesi

Supplementary Tables

Table S1. Summary results on PCR efficiency and conditions of the CYTB primer pair on honey bee and hemipteran specimens.

DNA sample/ species	Order	Suborder	DNA concentration (ng/µL)	52 °C annealing temperature	57 °C annealing temperature	64 °C annealing temperature
Apis mellifera ligustica 1	Hymenoptera	Apocrita	50	specific & nonspecific products	no amplification	no amplification
Apis mellifera ligustica 2			50	specific & nonspecific products	no amplification	no amplification
Apis mellifera ligustica 3			50	specific & nonspecific products	no amplification	no amplification
Apis mellifera carnica 1			50	specific & nonspecific products	no amplification	no amplification
Apis mellifera carnica 2			50	specific & nonspecific products	no amplification	no amplification
Apis mellifera carnica 3			50	specific & nonspecific products	no amplification	no amplification
Apis mellifera mellifera 1			50	specific & nonspecific products	no amplification	no amplification
Apis mellifera mellifera 2			50	specific & nonspecific products	no amplification	no amplification
Apis mellifera mellifera 3			50	specific & nonspecific products	no amplification	no amplification
Metcalfa pruinosa 1	Hemiptera	Auchenorrhyncha	50	nonspecific products	no amplification	no amplification
Metcalfa pruinosa 2			50	nonspecific products	no amplification	no amplification
Metcalfa pruinosa 3			50	nonspecific products	no amplification	no amplification
Issus muscaeformis 1			50	nonspecific products	specific product	no amplification
Issus muscaeformis 2			50	nonspecific products	specific product	no amplification
Issus muscaeformis 3			50	nonspecific products	specific product	no amplification
Lyristes plebejus 1			50	smearing	specific product	specific product
Lyristes plebejus 2			50	smearing	specific product	specific product
Lyristes plebejus 3			50	smearing	specific product	specific product
Halyomorpha halys 1		Heteroptera	50	nonspecific products	specific product	no amplification
Halyomorpha halys 2			50	nonspecific products	specific product	no amplification
Halyomorpha halys 3			50	nonspecific products	specific product	no amplification
Corythucha ciliata 1			50	nonspecific products	specific product	no amplification
Corythucha ciliata 2			50	nonspecific products	specific product	no amplification
Corythucha ciliata 3			50	nonspecific products	specific product	no amplification
Aphis craccivora 1		Sternorrhyncha	50	smearing	specific product	no amplification
Aphis craccivora 2			50	smearing	specific product	no amplification
Aphis craccivora 3			50	smearing	specific product	no amplification
Cinara cedri 1			50	smearing	specific product	specific product
Cinara cedri 2			50	smearing	specific product	specific product
Cinara cedri 3			50	smearing	specific product	specific product
Cinara cupressi 1			50	smearing	specific product	specific product
Cinara cupressi 2			50	smearing	specific product	specific product
Cinara cupressi 3			50	smearing	specific product	specific product
Cinara pectinata 1			50	smearing	specific product	specific product
Cinara pectinata 2			50	smearing	specific product	specific product
Cinara pectinata 3			50	smearing	specific product	specific product
Toumeyella parvicornis 1			50	smearing	specific product	no amplification
Toumeyella parvicornis 2			50	smearing	specific product	no amplification

Toumeyella parvicornis 3	50	smearing	specific product	no amplification
Icerya purchasi 1	50	nonspecific products	specific product	no amplification
Icerya purchasi 2	50	nonspecific products	specific product	no amplification
Icerya purchasi 3	50	nonspecific products	specific product	no amplification
Myzus persicae 1	50	smearing	specific product	no amplification
Myzus persicae 2	50	smearing	specific product	no amplification
Myzus persicae 3	50	smearing	specific product	no amplification

Table S2. Insect taxa identified through COI metabarcoding of the different honey samples investigated.

Honey ID	Taxon ID	Level	Taxon	Family	Order	Reads
HC_1	27482	family	Aphididae	Aphididae	Hemiptera	16130
	80764	genus	*Aphis*	Aphididae	Hemiptera	683
	80765	species	*Aphis gossypii*	Aphididae	Hemiptera	401
	13164	species	*Myzus persicae*	Aphididae	Hemiptera	142
	12998	genus	*Chaitophorus*	Aphididae	Hemiptera	56
	112272	genus	*Megoura*	Aphididae	Hemiptera	39
	330452	species	*Brachycaudus helichrysi*	Aphididae	Hemiptera	24
	527585	species	*Aphis sp.*	Aphididae	Hemiptera	12
	312887	genus	*Hyalopterus*	Aphididae	Hemiptera	12
	1425459	species	*Aphis lichtensteini*	Aphididae	Hemiptera	12
	33386	tribe	Macrosiphini	Aphididae	Hemiptera	7
	133076	subfamily	Aphidinae	Aphididae	Hemiptera	7
	97069	species	*Maculolachnus submacula*	Aphididae	Hemiptera	2
	87309	genus	*Uroleucon*	Aphididae	Hemiptera	2
	464929	subgenus	*Aphis*	Aphididae	Hemiptera	2
	384670	species	*Brachycaudus persicae*	Aphididae	Hemiptera	1
	13130	genus	*Macrosiphum*	Aphididae	Hemiptera	1
HC_2	27482	family	Aphididae	Aphididae	Hemiptera	38676
	80765	species	*Aphis gossypii*	Aphididae	Hemiptera	1180
	80764	genus	*Aphis*	Aphididae	Hemiptera	143
	1897835	species	*Issus coleoptratus*	Issidae	Hemiptera	43
	33386	tribe	Macrosiphini	Aphididae	Hemiptera	20
	13164	species	*Myzus persicae*	Aphididae	Hemiptera	4
	202455	genus	*Aulacorthum*	Aphididae	Hemiptera	3
	1656004	species	*Abstrusomyzus phloxae*	Aphididae	Hemiptera	3
	330452	species	*Brachycaudus helichrysi*	Aphididae	Hemiptera	2
	511039	species	*Aphis neospiraeae*	Aphididae	Hemiptera	1
	345564	species	*Aphis taraxacicola*	Aphididae	Hemiptera	1
	1889662	species	*Diuraphis sp.*	Aphididae	Hemiptera	1
HC_3	27482	family	Aphididae	Aphididae	Hemiptera	10159
	80765	species	*Aphis gossypii*	Aphididae	Hemiptera	213
	135956	species	*Thelaxes suberi*	Thelaxidae	Hemiptera	161
	506608	species	*Cinara cedri*	Aphididae	Hemiptera	54
	312887	genus	*Hyalopterus*	Aphididae	Hemiptera	37
	136351	species	*Chaitophorus leucomelas*	Aphididae	Hemiptera	25
	80764	genus	*Aphis*	Aphididae	Hemiptera	23
	511022	species	*Lipaphis pseudobrassicae*	Aphididae	Hemiptera	10
	527585	species	*Aphis sp.*	Aphididae	Hemiptera	4
	33386	tribe	Macrosiphini	Aphididae	Hemiptera	2
	112272	genus	*Megoura*	Aphididae	Hemiptera	2
	994501	subspecies	*Myzus cerasi cerasi*	Aphididae	Hemiptera	1
	96541	subfamily	*Lachninae*	Aphididae	Hemiptera	1
	13164	species	*Myzus persicae*	Aphididae	Hemiptera	1
HC_4	27482	family	Aphididae	Aphididae	Hemiptera	113725
	135956	species	*Thelaxes suberi*	Thelaxidae	Hemiptera	7367
	80765	species	*Aphis gossypii*	Aphididae	Hemiptera	1374

	527585	species	*Aphis sp.*	Aphididae	Hemiptera	1211
	80764	genus	*Aphis*	Aphididae	Hemiptera	558
	506608	species	*Cinara cedri*	Aphididae	Hemiptera	470
	214835	genus	*Dysaphis*	Aphididae	Hemiptera	465
	12998	genus	*Chaitophorus*	Aphididae	Hemiptera	290
	82742	species	*Orius laevigatus*	Anthocoridae	Hemiptera	270
	1897836	species	*Issus muscaeformis*	Issidae	Hemiptera	198
	312887	genus	*Hyalopterus*	Aphididae	Hemiptera	150
	2179459	species	*Aphis sp.*	Aphididae	Hemiptera	115
	13130	genus	*Macrosiphum*	Aphididae	Hemiptera	101
	136351	species	*Chaitophorus leucomelas*	Aphididae	Hemiptera	63
	511022	species	*Lipaphis pseudobrassicae*	Aphididae	Hemiptera	46
	13164	species	*Myzus persicae*	Aphididae	Hemiptera	26
	96541	subfamily	Lachninae	Aphididae	Hemiptera	22
	464929	subgenus	*Aphis*	Aphididae	Hemiptera	11
	33386	tribe	Macrosiphini	Aphididae	Hemiptera	11
	1889959	species	*Sitobion sp.*	Aphididae	Hemiptera	7
	330452	species	*Brachycaudus helichrysi*	Aphididae	Hemiptera	4
	112272	genus	*Megoura*	Aphididae	Hemiptera	3
	1425465	species	*Aphis proffti*	Aphididae	Hemiptera	2
	133076	subfamily	Aphidinae	Aphididae	Hemiptera	2
	715371	species	*Aphis anuraphoides*	Aphididae	Hemiptera	1
	464946	genus	*Aphis*	Aphididae	Hemiptera	1
	1452851	species	*Usingerida sp.*	Aradidae	Hemiptera	1
	136356	species	*Schizolachnus pineti*	Aphididae	Hemiptera	1
	1173396	species	*Greenidea prunicola*	Aphididae	Hemiptera	1
HC_5	27482	family	Aphididae	Aphididae	Hemiptera	64208
	1105439	family	Aphididae	Aphididae	Hemiptera	1425
	133092	species	*Mindarus abietinus*	Mindaridae	Hemiptera	1160
	80765	species	*Aphis gossypii*	Aphididae	Hemiptera	983
	506608	species	*Cinara cedri*	Aphididae	Hemiptera	459
	80764	genus	*Aphis*	Aphididae	Hemiptera	62
	1897836	species	*Issus muscaeformis*	Issidae	Hemiptera	29
	12998	genus	*Chaitophorus*	Aphididae	Hemiptera	18
	1656547	species	*Illinoia rubicola*	Aphididae	Hemiptera	15
	97063	species	*Cinara pseudotaxifoliae*	Aphididae	Hemiptera	7
	464929	subgenus	*Aphis*	Aphididae	Hemiptera	1
	345564	species	*Aphis taraxacicola*	Aphididae	Hemiptera	1
	163907	species	*Metopeurum fuscoviride*	Aphididae	Hemiptera	1
	13164	species	*Myzus persicae*	Aphididae	Hemiptera	1
	1300246	species	*Semiaphis dauci*	Aphididae	Hemiptera	1
HC_6	27482	family	Aphididae	Aphididae	Hemiptera	28451
	80765	species	*Aphis gossypii*	Aphididae	Hemiptera	596
	312887	genus	*Hyalopterus*	Aphididae	Hemiptera	478
	80764	genus	*Aphis*	Aphididae	Hemiptera	355
	527585	species	*Aphis sp.*	Aphididae	Hemiptera	203
	269402	genus	*Nasonovia*	Aphididae	Hemiptera	93
	112272	genus	*Megoura*	Aphididae	Hemiptera	50
	33386	tribe	Macrosiphini	Aphididae	Hemiptera	14
	330452	species	*Brachycaudus helichrysi*	Aphididae	Hemiptera	7

	345564	species	*Aphis taraxacicola*	Aphididae	Hemiptera	1
	224527	species	*Aphis spiraecola*	Aphididae	Hemiptera	1
	133076	subfamily	Aphidinae	Aphididae	Hemiptera	1
	13130	genus	*Macrosiphum*	Aphididae	Hemiptera	1
HC_7	27482	family	Aphididae	Aphididae	Hemiptera	89864
	1078267	species	*Mindarus keteleerifoliae*	Mindaridae	Hemiptera	7639
	80765	species	*Aphis gossypii*	Aphididae	Hemiptera	1767
	1897842	species	*Latissus dilatatus*	Issidae	Hemiptera	312
	80764	genus	*Aphis*	Aphididae	Hemiptera	162
	581208	species	*Pineus sp.*	Adelgidae	Hemiptera	26
	13152	genus	*Mindarus*	Mindaridae	Hemiptera	25
	133076	subfamily	Aphidinae	Aphididae	Hemiptera	13
	330452	species	*Brachycaudus helichrysi*	Aphididae	Hemiptera	6
	236622	genus	*Nysius*	Lygaeidae	Hemiptera	6
	419076	genus	*Pineus*	Adelgidae	Hemiptera	5
	464929	subgenus	*Aphis*	Aphididae	Hemiptera	3
	345564	species	*Aphis taraxacicola*	Aphididae	Hemiptera	3
	805322	genus	*Yamatochaitophorus*	Aphididae	Hemiptera	1
	796231	species	*Kurisakia querciphila*	Aphididae	Hemiptera	1
	419086	species	*Pineus coloradensis*	Adelgidae	Hemiptera	1
	384362	species	*Kurisakia onigurumii*	Thelaxidae	Hemiptera	1
	312887	genus	*Hyalopterus*	Aphididae	Hemiptera	1
	2027652	species	*Periphyllus bengalensis*	Aphididae	Hemiptera	1
	1452851	species	*Usingerida sp.*	Aradidae	Hemiptera	1
	136351	species	*Chaitophorus leucomelas*	Aphididae	Hemiptera	1
	133092	species	*Mindarus abietinus*	Mindaridae	Hemiptera	1
	13164	species	*Myzus persicae*	Aphididae	Hemiptera	1
HC_8	27482	family	Aphididae	Aphididae	Hemiptera	41196
	80765	species	*Aphis gossypii*	Aphididae	Hemiptera	1226
	80764	genus	*Aphis*	Aphididae	Hemiptera	156
	13164	species	*Myzus persicae*	Aphididae	Hemiptera	15
	202455	genus	*Aulacorthum*	Aphididae	Hemiptera	9
	330452	species	*Brachycaudus helichrysi*	Aphididae	Hemiptera	5
	469903	species	*Megoura lespedezae*	Aphididae	Hemiptera	2
	464929	subgenus	*Aphis*	Aphididae	Hemiptera	2
	345564	species	*Aphis taraxacicola*	Aphididae	Hemiptera	2
	33386	tribe	Macrosiphini	Aphididae	Hemiptera	2
	464946	genus	*Aphis*	Aphididae	Hemiptera	1
	112272	genus	*Megoura*	Aphididae	Hemiptera	1
HC_9	27482	family	Aphididae	Aphididae	Hemiptera	23551
	511031	species	*Aphis gossypii*	Aphididae	Hemiptera	854
	1078267	species	*Mindarus keteleerifoliae*	Mindaridae	Hemiptera	430
	112272	genus	Megoura	Aphididae	Hemiptera	80
	33386	tribe	Macrosiphini	Aphididae	Hemiptera	23
	80764	genus	*Aphis*	Aphididae	Hemiptera	13
	464929	subgenus	*Aphis*	Aphididae	Hemiptera	2
	13152	genus	*Mindarus*	Mindaridae	Hemiptera	2
	796231	species	*Kurisakia querciphila*	Thelaxidae	Hemiptera	1
	527585	species	*Aphis sp. B RGF-2008*	Aphididae	Hemiptera	1
HC_10	27482	family	Aphididae	Aphididae	Hemiptera	56876

	527585	species	*Aphis sp.*	Aphididae	Hemiptera	2841
	80764	genus	*Aphis*	Aphididae	Hemiptera	1119
	80765	species	*Aphis gossypii*	Aphididae	Hemiptera	1101
	133092	species	*Mindarus abietinus*	Mindaridae	Hemiptera	140
	506608	species	*Cinara cedri*	Aphididae	Hemiptera	126
	13164	species	*Myzus persicae*	Aphididae	Hemiptera	29
	12998	genus	*Chaitophorus*	Aphididae	Hemiptera	20
	464929	subgenus	*Aphis*	Aphididae	Hemiptera	9
	136351	species	*Chaitophorus leucomelas*	Aphididae	Hemiptera	7
	464946	genus	*Aphis*	Aphididae	Hemiptera	2
	527582	species	*Aphis pomi*	Aphididae	Hemiptera	1
	511036	species	*Aphis ichigo*	Aphididae	Hemiptera	1
	136356	species	*Schizolachnus pineti*	Aphididae	Hemiptera	1
HC_11	27482	family	Aphididae	Aphididae	Hemiptera	50463
	133092	species	Mindarus abietinus	Mindaridae	Hemiptera	1987
	80765	species	Aphis gossypii	Aphididae	Hemiptera	1190
	80764	genus	Aphis	Aphididae	Hemiptera	773
	527585	species	Aphis sp.	Aphididae	Hemiptera	479
	330452	species	Brachycaudus helichrysi	Aphididae	Hemiptera	195
	312887	genus	Hyalopterus	Aphididae	Hemiptera	193
	2546820	species	Pachypappa warshavensis	Pemphigidae	Hemiptera	8
	384362	species	Kurisakia onigurumii	Thelaxidae	Hemiptera	2
	12998	genus	Chaitophorus	Aphididae	Hemiptera	2
	112272	genus	Megoura	Aphididae	Hemiptera	2
	904942	species	Tuberculatus pilosus	Aphididae	Hemiptera	1
	464929	subgenus	Aphis	Aphididae	Hemiptera	1
	13164	species	Myzus persicae	Aphididae	Hemiptera	1
	1300245	species	Myzocallis castanicola	Aphididae	Hemiptera	1
HC_12	27482	family	Aphididae	Aphididae	Hemiptera	55704
	527585	species	*Aphis sp.*	Aphididae	Hemiptera	1416
	80765	species	*Aphis gossypii*	Aphididae	Hemiptera	1089
	80764	genus	*Aphis*	Aphididae	Hemiptera	841
	1897836	species	*Issus muscaeformis*	Issidae	Hemiptera	192
	464929	subgenus	*Aphis*	Aphididae	Hemiptera	136
	13164	species	*Myzus persicae*	Aphididae	Hemiptera	104
	12998	genus	*Chaitophorus*	Aphididae	Hemiptera	14
	33387	tribe	Aphidini	Aphididae	Hemiptera	9
	581208	species	*Pineus sp.*	Adelgidae	Hemiptera	3
	345564	species	*Aphis taraxacicola*	Aphididae	Hemiptera	3
	136351	species	*Chaitophorus leucomelas*	Aphididae	Hemiptera	3
	133076	subfamily	Aphidinae	Aphididae	Hemiptera	3
	464946	genus	*Aphis*	Aphididae	Hemiptera	2
	312887	genus	*Hyalopterus*	Aphididae	Hemiptera	2
	486029	species	*Brachycaudus salicinae*	Aphididae	Hemiptera	1
	33386	tribe	Macrosiphini	Aphididae	Hemiptera	1
	1425459	species	*Aphis lichtensteini*	Aphididae	Hemiptera	1
	112272	genus	*Megoura*	Aphididae	Hemiptera	1
HB_1A1	1185500	species	*Metcalfa pruinosa*	Flatidae	Hemiptera	10029
	30089	family	Flatidae	Flatidae	Hemiptera	4458
	27482	family	Aphididae	Aphididae	Hemiptera	2232

	1425451	species	*Aphis cisticola*	Aphididae	Hemiptera	778
	80764	genus	*Aphis*	Aphididae	Hemiptera	437
	506608	species	*Cinara cedri*	Aphididae	Hemiptera	382
	82741	genus	*Orius*	Anthocoridae	Hemiptera	300
	12998	genus	*Chaitophorus*	Aphididae	Hemiptera	166
	136351	species	*Chaitophorus leucomelas*	Aphididae	Hemiptera	71
	82742	species	*Orius laevigatus*	Anthocoridae	Hemiptera	12
	80765	species	*Aphis gossypii*	Aphididae	Hemiptera	7
	796232	species	*Macrosiphoniella formosartemisiae*	Aphididae	Hemiptera	1
	2351320	family	Cecidomyiidae sp. BIOUG21204-D02	Cecidomyiidae	Diptera	1
HB_1A2	1185500	species	*Metcalfa pruinosa*	Flatidae	Hemiptera	59518
	30089	family	Flatidae	Flatidae	Hemiptera	21038
	80764	genus	*Aphis*	Aphididae	Hemiptera	64
	82742	species	*Orius laevigatus*	Anthocoridae	Hemiptera	12
	135956	species	*Thelaxes suberi*	Thelaxidae	Hemiptera	9
	87307	genus	*Macrosiphoniella*	Aphididae	Hemiptera	3
	506608	species	*Cinara cedri*	Aphididae	Hemiptera	2
	384362	species	*Kurisakia onigurumii*	Thelaxidae	Hemiptera	1
	1897835	species	*Issus coleoptratus*	Issidae	Hemiptera	1
	1425451	species	*Aphis cisticola*	Aphididae	Hemiptera	1
	13262	species	*Schizaphis graminum*	Aphididae	Hemiptera	1
HB_1C1	715371	species	*Aphis anuraphoides*	Aphididae	Hemiptera	520
	1185500	species	*Metcalfa pruinosa*	Flatidae	Hemiptera	86
	30089	family	Flatidae	Flatidae	Hemiptera	27
	135956	species	*Thelaxes suberi*	Thelaxidae	Hemiptera	6
	41112	species	*Oryzaephilus surinamensis*	Silvanidae	Coleoptera	5
	87307	genus	*Macrosiphoniella*	Aphididae	Hemiptera	4
	82742	species	*Orius laevigatus*	Anthocoridae	Hemiptera	4
	80764	genus	*Aphis*	Aphididae	Hemiptera	2
	796232	species	*Macrosiphoniella formosartemisiae*	Aphididae	Hemiptera	2
	27482	family	Aphididae	Aphididae	Hemiptera	2
	1078272	species	*Schizolachnus orientalis*	Aphididae	Hemiptera	2
	506608	species	*Cinara cedri*	Aphididae	Hemiptera	1
	1889932	species	*Macrosiphoniella sp. BOLD-2016*	Aphididae	Hemiptera	1
HB_2A2	135956	species	*Thelaxes suberi*	Thelaxidae	Hemiptera	16175
	1185500	species	*Metcalfa pruinosa*	Flatidae	Hemiptera	13924
	30089	family	Flatidae	Flatidae	Hemiptera	4784
	506608	species	*Cinara cedri*	Aphididae	Hemiptera	108
	1897835	species	*Issus coleoptratus*	Issidae	Hemiptera	60
	40931	genus	*Rhopalosiphum*	Aphididae	Hemiptera	47
	1657232	species	*Tuberculatus querceus*	Aphididae	Hemiptera	45
	2781717	species	*Meloe mediterraneus*	Meloidae	Coleoptera	42
	80764	genus	*Aphis*	Aphididae	Hemiptera	29
	99934	species	*Lachnus roboris*	Aphididae	Hemiptera	13
	97051	genus	*Cinara*	Aphididae	Hemiptera	4
	87307	genus	*Macrosiphoniella*	Aphididae	Hemiptera	2
	749392	species	*Phylloxera coccinea*	Phylloxeridae	Hemiptera	2
	41112	species	*Oryzaephilus surinamensis*	Silvanidae	Coleoptera	2
	82742	species	*Orius laevigatus*	Anthocoridae	Hemiptera	1
	796232	species	*Macrosiphoniella formosartemisiae*	Aphididae	Hemiptera	1

	33386	tribe	Macrosiphini	Aphididae	Hemiptera	1
	133066	genus	*Anoecia*	Anoeciidae	Hemiptera	1
	1295445	species	*Ortholomia sp. BOLD:AAL8407*	Notodontidae	Lepidoptera	1
HB_2B2	1185500	species	*Metcalfa pruinosa*	Flatidae	Hemiptera	8549
	506608	species	*Cinara cedri*	Aphididae	Hemiptera	4406
	30089	family	Flatidae	Flatidae	Hemiptera	2523
	135956	species	*Thelaxes suberi*	Thelaxidae	Hemiptera	717
	97051	genus	*Cinara*	Aphididae	Hemiptera	47
	1657232	species	*Tuberculatus querceus*	Aphididae	Hemiptera	31
	1897835	species	*Issus coleoptratus*	Issidae	Hemiptera	30
	41112	species	*Oryzaephilus surinamensis*	Silvanidae	Coleoptera	19
	40931	genus	*Rhopalosiphum*	Aphididae	Hemiptera	13
	80764	genus	*Aphis*	Aphididae	Hemiptera	6
	1425451	species	*Aphis cisticola*	Aphididae	Hemiptera	4
HB_2C2	1185500	species	*Metcalfa pruinosa*	Flatidae	Hemiptera	40463
	30089	family	Flatidae	Flatidae	Hemiptera	11503
	506608	species	*Cinara cedri*	Aphididae	Hemiptera	9516
	135956	species	*Thelaxes suberi*	Thelaxidae	Hemiptera	5759
	1425451	species	*Aphis cisticola*	Aphididae	Hemiptera	2184
	1897835	species	*Issus coleoptratus*	Issidae	Hemiptera	404
	80764	genus	*Aphis*	Aphididae	Hemiptera	350
	41112	species	*Oryzaephilus surinamensis*	Silvanidae	Coleoptera	239
	97051	genus	*Cinara*	Aphididae	Hemiptera	54
	2781717	species	*Meloe mediterraneus*	Meloidae	Coleoptera	13
	96541	subfamily	Lachninae	Aphididae	Hemiptera	1
	527605	species	*Cinara fornacula*	Aphididae	Hemiptera	1
	27482	family	Aphididae	Aphididae	Hemiptera	1
	214835	genus	*Dysaphis*	Aphididae	Hemiptera	1
	1897833	genus	*Issus*	Issidae	Hemiptera	1
HB_3A3	1185500	species	*Metcalfa pruinosa*	Flatidae	Hemiptera	53389
	30089	family	Flatidae	Flatidae	Hemiptera	19116
	1897835	species	*Issus coleoptratus*	Issidae	Hemiptera	931
	135956	species	*Thelaxes suberi*	Thelaxidae	Hemiptera	107
	1897842	species	*Latissus dilatatus*	Issidae	Hemiptera	23
	80764	genus	*Aphis*	Aphididae	Hemiptera	21
	2776077	species	*Paraperithous gnathaulax*	Ichneumonidae	Hymenoptera	6
	1212802	species	*Niphonyx segregata*	Noctuidae	Lepidoptera	2
	80765	species	*Aphis gossypii*	Aphididae	Hemiptera	1
	568383	species	*Mompha sp. JFL01*	Momphidae	Lepidoptera	1
	1425451	species	*Aphis cisticola*	Aphididae	Hemiptera	1
	1310355	species	*Odonestis pruni*	Lasiocampidae	Lepidoptera	1
HB_3B3	1185500	species	*Metcalfa pruinosa*	Flatidae	Hemiptera	38534
	30089	family	Flatidae	Flatidae	Hemiptera	20751
	1897835	species	*Issus coleoptratus*	Issidae	Hemiptera	1583
	135956	species	*Thelaxes suberi*	Thelaxidae	Hemiptera	696
	506608	species	*Cinara cedri*	Aphididae	Hemiptera	325
	41112	species	*Oryzaephilus surinamensis*	Silvanidae	Coleoptera	149
	12998	genus	*Chaitophorus*	Aphididae	Hemiptera	60
	82744	species	*Orius minutus*	Anthocoridae	Hemiptera	2
	464929	subgenus	*Aphis*	Aphididae	Hemiptera	2

	13262	species	*Schizaphis graminum*	Aphididae	Hemiptera	2
	1897833	genus	*Issus*	Issidae	Hemiptera	1
	1308479	species	*Epeurysa nawaii*	Delphacidae	Hemiptera	1
HT_1	1425451	species	*Aphis cisticola*	Aphididae	Hemiptera	401
	195883	species	*Laodelphax striatellus*	Delphacidae	Hemiptera	212
	13262	species	*Schizaphis graminum*	Aphididae	Hemiptera	187
	80764	genus	*Aphis*	Aphididae	Hemiptera	35
	27482	family	Aphididae	Aphididae	Hemiptera	3
	136351	species	*Chaitophorus leucomelas*	Aphididae	Hemiptera	3
	2819898	species	*Orosanga japonica*	Ricaniidae	Hemiptera	2
	80765	species	*Aphis gossypii*	Aphididae	Hemiptera	1
	506608	species	*Cinara cedri*	Aphididae	Hemiptera	1
	33386	tribe	Macrosiphini	Aphididae	Hemiptera	1
	329961	family	Cecidomyiidae	Cecidomyiidae	Diptera	1
	30089	family	Flatidae	Flatidae	Hemiptera	1
	13261	genus	*Schizaphis*	Aphididae	Hemiptera	1
	1185500	species	*Metcalfa pruinosa*	Flatidae	Hemiptera	1
	1009738	species	*Cranophorus sp. AG06*	Coccinellidae	Coleoptera	1
HT_3	27482	family	Aphididae	Aphididae	Hemiptera	24999
	80764	genus	*Aphis*	Aphididae	Hemiptera	1556
	464929	subgenus	*Aphis*	Aphididae	Hemiptera	397
	80765	species	*Aphis gossypii*	Aphididae	Hemiptera	225
	345549	species	*Aphis craccae*	Aphididae	Hemiptera	176
	13164	species	*Myzus persicae*	Aphididae	Hemiptera	134
	511031	species	*Aphis egomae*	Aphididae	Hemiptera	61
	1425469	species	*Aphis serpylli*	Aphididae	Hemiptera	41
	511036	species	*Aphis ichigo*	Aphididae	Hemiptera	27
	1425466	species	*Aphis pseudocomosa*	Aphididae	Hemiptera	16
	133076	subfamily	Aphidinae	Aphididae	Hemiptera	8
	34750	genus	*Meloe*	Meloidae	Coleoptera	4
	506608	species	*Cinara cedri*	Aphididae	Hemiptera	2
	236622	genus	*Nysius*	Lygaeidae	Hemiptera	2
	1425448	species	*Aphis brotericola*	Aphididae	Hemiptera	2
	527585	species	*Aphis sp. B RGF-2008*	Aphididae	Hemiptera	1
	527517	species	*Acyrthosiphon purshiae*	Aphididae	Hemiptera	1
	464946	genus	*Aphis*	Aphididae	Hemiptera	1
	2781714	species	*Meloe glazunovi*	Meloidae	Coleoptera	1
	1452851	species	*Usingerida sp. EH001*	Aradidae	Hemiptera	1
	1425451	species	*Aphis cisticola*	Aphididae	Hemiptera	1
HT_13	506608	species	*Cinara cedri*	Aphididae	Hemiptera	36435
	135956	species	*Thelaxes suberi*	Thelaxidae	Hemiptera	828
	97051	genus	*Cinara*	Aphididae	Hemiptera	426
	1371672	species	*Cadra figulilella*	Pyralidae	Lepidoptera	184
	27482	family	Aphididae	Aphididae	Hemiptera	73
	2638729	no rank	*Cinara*	Aphididae	Hemiptera	38
	96541	subfamily	Lachninae	Aphididae	Hemiptera	4
	41112	species	*Oryzaephilus surinamensis*	Silvanidae	Coleoptera	3
	506603	species	*Cinara juniperi*	Aphididae	Hemiptera	1
	384377	species	*Dasyaphis rhusae*	Aphididae	Hemiptera	1
	2819898	species	*Orosanga japonica*	Ricaniidae	Hemiptera	1

	1009738	species	*Cranophorus sp. AG06*	Coccinellidae	Coleoptera	1
HT_19	506608	species	*Cinara cedri*	Aphididae	Hemiptera	81488
	80764	genus	*Aphis*	Aphididae	Hemiptera	51
	136351	species	*Chaitophorus leucomelas*	Aphididae	Hemiptera	42
	527713	species	*Liosomaphis berberidis*	Aphididae	Hemiptera	30
	34750	genus	*Meloe*	Meloidae	Coleoptera	14
	27482	family	Aphididae	Aphididae	Hemiptera	13
	224525	genus	*Brachycaudus*	Aphididae	Hemiptera	13
	1425469	species	*Aphis serpylli*	Aphididae	Hemiptera	7
	1425451	species	*Aphis cisticola*	Aphididae	Hemiptera	6
	96541	subfamily	Lachninae	Aphididae	Hemiptera	4
	236622	genus	*Nysius*	Lygaeidae	Hemiptera	3
	527605	species	*Cinara fornacula*	Aphididae	Hemiptera	2
	527680	genus	*Eucallipterus*	Aphididae	Hemiptera	1
	33386	tribe	Macrosiphini	Aphididae	Hemiptera	1
	1421472	species	*Cinara palaestinensis*	Aphididae	Hemiptera	1
	135956	species	*Thelaxes suberi*	Thelaxidae	Hemiptera	1
	1185500	species	*Metcalfa pruinosa*	Flatidae	Hemiptera	1
HT_28	135956	species	*Thelaxes suberi*	Thelaxidae	Hemiptera	103309
	1421472	species	*Cinara palaestinensis*	Aphididae	Hemiptera	174
	136355	genus	*Schizolachnus*	Aphididae	Hemiptera	14
	703277	species	*Scymnus subvillosus*	Coccinellidae	Coleoptera	12
	1185500	species	*Metcalfa pruinosa*	Flatidae	Hemiptera	7
	27482	family	Aphididae	Aphididae	Hemiptera	3
	30089	family	Flatidae	Flatidae	Hemiptera	2
	1371672	species	*Cadra figulilella*	Pyralidae	Lepidoptera	2
	527713	species	*Liosomaphis berberidis*	Aphididae	Hemiptera	1
	527685	species	*Gypsoaphis oestlundi*	Aphididae	Hemiptera	1
	506608	species	*Cinara cedri*	Aphididae	Hemiptera	1
	2819898	species	*Orosanga japonica*	Ricaniidae	Hemiptera	1
	2597398	species	*Cecidomyia pini*	Cecidomyiidae	Diptera	1
	13040	species	*Diaprepes abbreviatus*	Curculionidae	Coleoptera	1
	1295445	species	*Ortholomia sp. BOLD:AAL8407*	Notodontidae	Lepidoptera	1

Table S3. Insect taxa identified through CYTB metabarcoding of the different honey samples investigated.

Honey ID	Taxon ID	Level	Taxon	Family	Order	Reads
HC_1	464929	subgenus	*Aphis*	Aphididae	Hemiptera	76055
	7029	species	*Acyrthosiphon pisum*	Aphididae	Hemiptera	15192
	13164	species	*Myzus persicae*	Aphididae	Hemiptera	11043
	1276923	species	*Hyalopterus amygdali*	Aphididae	Hemiptera	3946
	198323	species	*Cinara tujafilina*	Aphididae	Hemiptera	3814
	469905	species	*Megoura nigra*	Aphididae	Hemiptera	3380
	80764	genus	*Aphis*	Aphididae	Hemiptera	2523
	33386	tribe	Macrosiphini	Aphididae	Hemiptera	1231
	27482	family	Aphididae	Aphididae	Hemiptera	1090

330460	species	*Hyperomyzus lactucae*	Aphididae	Hemiptera	894
224527	species	*Aphis spiraecola*	Aphididae	Hemiptera	844
202396	species	*Rhopalosiphum padi*	Aphididae	Hemiptera	709
69196	species	*Brevicoryne brassicae*	Aphididae	Hemiptera	612
542820	species	*Chromaphis juglandicola*	Aphididae	Hemiptera	483
133076	subfamily	Aphidinae	Aphididae	Hemiptera	352
511022	species	*Lipaphis pseudobrassicae*	Aphididae	Hemiptera	334
44664	species	*Sitobion avenae*	Aphididae	Hemiptera	290
486035	species	*Macrosiphum sp. C1774*	Aphididae	Hemiptera	284
312888	species	*Hyalopterus pruni*	Aphididae	Hemiptera	214
464927	species	*Aphis odinae*	Aphididae	Hemiptera	138
935269	species	*Therioaphis trifolii*	Aphididae	Hemiptera	123
400569	species	*Aphis nasturtii*	Aphididae	Hemiptera	101
758717	species	*Acleris sparsana*	Tortricidae	Lepidoptera	77
1078272	species	*Schizolachnus orientalis*	Aphididae	Hemiptera	72
80765	species	*Aphis gossypii*	Aphididae	Hemiptera	45
464926	species	*Aphis aurantii*	Aphididae	Hemiptera	45
796229	species	*Greenidea ficicola*	Aphididae	Hemiptera	35
33387	tribe	Aphidini	Aphididae	Hemiptera	30
12998	genus	*Chaitophorus*	Aphididae	Hemiptera	27
464930	subgenus	*Bursaphis*	Aphididae	Hemiptera	21
511041	species	*Aphis sanguisorbicola*	Aphididae	Hemiptera	19
2024751	species	*Mahasena oolona*	Psychidae	Lepidoptera	14
1417364	species	*Athyma kasa*	Nymphalidae	Lepidoptera	13
2020618	subspecies	*Chaitophorus saliapterus quinquemaculatus*	Aphididae	Hemiptera	11
511039	species	*Aphis neospiraeae*	Aphididae	Hemiptera	9
168652	species	*Rhopalosiphum insertum*	Aphididae	Hemiptera	7
486037	species	*Anuraphis pyrilaseri*	Aphididae	Hemiptera	6
476189	species	*Rhopalosiphum musae*	Aphididae	Hemiptera	6
345546	species	*Aphis armata*	Aphididae	Hemiptera	6
351416	genus	*Athyma*	Nymphalidae	Lepidoptera	4
87309	genus	*Uroleucon*	Aphididae	Hemiptera	3
702717	species	*Tuta absoluta*	Gelechiidae	Lepidoptera	3
488727	species	*Astegopteryx styracophila*	Aphididae	Hemiptera	3
334015	species	*Coreana raphaelis*	Lycaenidae	Lepidoptera	3
224525	genus	*Brachycaudus*	Aphididae	Hemiptera	3
1350445	species	*Dermaphis crematogastri*	Aphididae	Hemiptera	3
1134961	subspecies	*Morpho sulkowskyi calderoni*	Nymphalidae	Lepidoptera	3
915061	species	*Uroleucon leonardi*	Aphididae	Hemiptera	2
51655	species	*Plutella xylostella*	Plutellidae	Lepidoptera	2
506608	species	*Cinara cedri*	Aphididae	Hemiptera	2
384385	species	*Macrosiphoniella yomogifoliae*	Aphididae	Hemiptera	2
1350441	species	*Nipponaphis sp.*	Hormaphididae	Hemiptera	2
1086193	species	*Barbourion lemaii*	Sphingidae	Lepidoptera	2
97055	genus	*Lachnus*	Aphididae	Hemiptera	1
97051	genus	*Cinara*	Aphididae	Hemiptera	1
96551	species	*Tuberolachnus salignus*	Aphididae	Hemiptera	1
910915	species	*Aphidura bozhkoae*	Aphididae	Hemiptera	1
685722	species	*Aulacorthum cirsicola*	Aphididae	Hemiptera	1
527880	species	*Schizolachnus piniradiatae*	Aphididae	Hemiptera	1

	511038	species	*Aphis kurosawai*	Aphididae	Hemiptera	1
	511030	species	*Aphis crinosa*	Aphididae	Hemiptera	1
	486027	species	*Brachycaudus prunicola*	Aphididae	Hemiptera	1
	486023	species	*Brachycaudus malvae*	Aphididae	Hemiptera	1
	476190	species	*Rhopalosiphum sp. X IL-2007*	Aphididae	Hemiptera	1
	464723	species	*Eilema bicolor*	Erebidae	Lepidoptera	1
	40932	species	*Rhopalosiphum padi*	Aphididae	Hemiptera	1
	384362	species	*Kurisakia onigurumii*	Thelaxidae	Hemiptera	1
	36997	species	*Ceratoglyphina styracicola*	Hormaphididae	Hemiptera	1
	345560	species	*Aphis rumicis*	Aphididae	Hemiptera	1
	223852	species	*Aphis citricidus*	Aphididae	Hemiptera	1
	2082423	species	*Ourapteryx adonidaria*	Geometridae	Lepidoptera	1
	206540	species	*Polyura narcaeus*	Nymphalidae	Lepidoptera	1
	1667255	species	*Macropodaphis sp. YW-2015*	Aphididae	Hemiptera	1
	134436	subspecies	*Spodoptera mauritia acronyctoides*	Noctuidae	Lepidoptera	1
	13262	species	*Schizaphis graminum*	Aphididae	Hemiptera	1
	1244007	species	*Cinara costata*	Aphididae	Hemiptera	1
	1087486	species	*Kentrochrysalis streckeri*	Sphingidae	Lepidoptera	1
HC_2	464929	subgenus	*Aphis*	Aphididae	Hemiptera	25571
	13164	species	*Myzus persicae*	Aphididae	Hemiptera	830
	69196	species	*Brevicoryne brassicae*	Aphididae	Hemiptera	427
	80764	genus	*Aphis*	Aphididae	Hemiptera	351
	486035	species	*Macrosiphum sp. C1774*	Aphididae	Hemiptera	252
	33386	tribe	Macrosiphini	Aphididae	Hemiptera	225
	488727	species	*Astegopteryx styracophila*	Aphididae	Hemiptera	220
	511022	species	*Lipaphis pseudobrassicae*	Aphididae	Hemiptera	144
	44664	species	*Sitobion avenae*	Aphididae	Hemiptera	86
	330460	species	*Hyperomyzus lactucae*	Aphididae	Hemiptera	74
	400569	species	*Aphis nasturtii*	Aphididae	Hemiptera	51
	224527	species	*Aphis spiraecola*	Aphididae	Hemiptera	50
	7029	species	*Acyrthosiphon pisum*	Aphididae	Hemiptera	48
	33385	superfamily	Aphidoidea	Aphididae	Hemiptera	35
	27482	family	Aphididae	Aphididae	Hemiptera	29
	312888	species	*Hyalopterus pruni*	Aphididae	Hemiptera	26
	202396	species	*Rhopalosiphum padi*	Aphididae	Hemiptera	24
	133076	subfamily	Aphidinae	Aphididae	Hemiptera	23
	469905	species	*Megoura nigra*	Aphididae	Hemiptera	22
	80765	species	*Aphis gossypii*	Aphididae	Hemiptera	17
	1276923	species	*Hyalopterus amygdali*	Aphididae	Hemiptera	14
	2572971	subfamily	Greenideinae	Aphididae	Hemiptera	12
	33387	tribe	Aphidini	Aphididae	Hemiptera	6
	1421473	species	*Cinara pectinatae*	Aphididae	Hemiptera	6
	1078272	species	*Schizolachnus orientalis*	Aphididae	Hemiptera	6
	2020618	subspecies	*Chaitophorus saliapterus quinquemaculatus*	Aphididae	Hemiptera	4
	12998	genus	*Chaitophorus*	Aphididae	Hemiptera	3
	76195	species	*Papilio helenus*	Papilionidae	Lepidoptera	2
	511039	species	*Aphis neospiraeae*	Aphididae	Hemiptera	2
	2024751	species	*Mahasena oolona*	Psychidae	Lepidoptera	2
	991824	species	*Aphis monardae*	Aphididae	Hemiptera	1
	910915	species	*Aphidura bozhkoae*	Aphididae	Hemiptera	1

	796220	species	*Aulacorthum vandenboschi*	Aphididae	Hemiptera	1
	311142	species	*Baeotus beotus*	Nymphalidae	Lepidoptera	1
	224525	genus	*Brachycaudus*	Aphididae	Hemiptera	1
	198323	species	*Cinara tujafilina*	Aphididae	Hemiptera	1
	168652	species	*Rhopalosiphum insertum*	Aphididae	Hemiptera	1
HC_3	464929	subgenus	*Aphis*	Aphididae	Hemiptera	20868
	13164	species	*Myzus persicae*	Aphididae	Hemiptera	929
	80764	genus	*Aphis*	Aphididae	Hemiptera	356
	1276923	species	*Hyalopterus amygdali*	Aphididae	Hemiptera	341
	469905	species	*Megoura nigra*	Aphididae	Hemiptera	192
	486035	species	*Macrosiphum sp.*	Aphididae	Hemiptera	186
	511022	species	*Lipaphis pseudobrassicae*	Aphididae	Hemiptera	184
	198323	species	*Cinara tujafilina*	Aphididae	Hemiptera	155
	7029	species	*Acyrthosiphon pisum*	Aphididae	Hemiptera	118
	133076	subfamily	Aphidinae	Aphididae	Hemiptera	44
	33386	tribe	Macrosiphini	Aphididae	Hemiptera	42
	488727	species	*Astegopteryx styracophila*	Hormaphididae	Hemiptera	37
	312888	species	*Hyalopterus pruni*	Aphididae	Hemiptera	35
	44664	species	*Sitobion avenae*	Aphididae	Hemiptera	31
	224527	species	*Aphis spiraecola*	Aphididae	Hemiptera	25
	80765	species	*Aphis gossypii*	Aphididae	Hemiptera	23
	527763	species	*Myzocallis coryli*	Aphididae	Hemiptera	20
	506608	species	*Cinara cedri*	Aphididae	Hemiptera	16
	69196	species	*Brevicoryne brassicae*	Aphididae	Hemiptera	14
	202396	species	*Rhopalosiphum padi*	Aphididae	Hemiptera	14
	33387	tribe	Aphidini	Aphididae	Hemiptera	13
	224525	genus	*Brachycaudus*	Aphididae	Hemiptera	12
	27482	family	Aphididae	Aphididae	Hemiptera	11
	935269	species	*Therioaphis trifolii*	Aphididae	Hemiptera	9
	1350445	species	*Dermaphis crematogastri*	Hormaphididae	Hemiptera	8
	330460	species	*Hyperomyzus lactucae*	Aphididae	Hemiptera	6
	1078272	species	*Schizolachnus orientalis*	Aphididae	Hemiptera	5
	76195	species	*Papilio helenus*	Papilionidae	Lepidoptera	3
	706528	species	*Apocheima cinerarius*	Geometridae	Lepidoptera	1
	685722	species	*Aulacorthum cirsicola*	Aphididae	Hemiptera	1
	51655	species	*Plutella xylostella*	Plutellidae	Lepidoptera	1
	511041	species	*Aphis sanguisorbicola*	Aphididae	Hemiptera	1
	33415	family	Nymphalidae	Nymphalidae	Lepidoptera	1
	2020618	subspecies	*Chaitophorus saliapterus quinquemaculatus*	Aphididae	Hemiptera	1
HC_4	464929	subgenus	*Aphis*	Aphididae	Hemiptera	87394
	486035	species	*Macrosiphum sp. C1774*	Aphididae	Hemiptera	6189
	13164	species	*Myzus persicae*	Aphididae	Hemiptera	4092
	469905	species	*Megoura nigra*	Aphididae	Hemiptera	1483
	80764	genus	*Aphis*	Aphididae	Hemiptera	1459
	33386	tribe	Macrosiphini	Aphididae	Hemiptera	1185
	198323	species	*Cinara tujafilina*	Aphididae	Hemiptera	1115
	44664	species	*Sitobion avenae*	Aphididae	Hemiptera	803
	511022	species	*Lipaphis pseudobrassicae*	Aphididae	Hemiptera	752
	1276923	species	*Hyalopterus amygdali*	Aphididae	Hemiptera	695
	27482	family	Aphididae	Aphididae	Hemiptera	507

7029	species	*Acyrthosiphon pisum*	Aphididae	Hemiptera	459
224525	genus	*Brachycaudus*	Aphididae	Hemiptera	364
312888	species	*Hyalopterus pruni*	Aphididae	Hemiptera	220
224527	species	*Aphis spiraecola*	Aphididae	Hemiptera	194
133076	subfamily	Aphidinae	Aphididae	Hemiptera	191
488727	species	*Astegopteryx styracophila*	Hormaphididae	Hemiptera	166
1078272	species	*Schizolachnus orientalis*	Aphididae	Hemiptera	166
527763	species	*Myzocallis coryli*	Aphididae	Hemiptera	108
80765	species	*Aphis gossypii*	Aphididae	Hemiptera	92
400569	species	*Aphis nasturtii*	Aphididae	Hemiptera	87
1667255	species	*Macropodaphis sp. YW-2015*	Aphididae	Hemiptera	70
33387	tribe	Aphidini	Aphididae	Hemiptera	68
202396	species	*Rhopalosiphum padi*	Aphididae	Hemiptera	67
2020618	subspecies	*Chaitophorus saliapterus quinquemaculatus*	Aphididae	Hemiptera	47
330460	species	*Hyperomyzus lactucae*	Aphididae	Hemiptera	40
442005	species	*Aulocera padma*	Nymphalidae	Lepidoptera	33
506608	species	*Cinara cedri*	Aphididae	Hemiptera	23
168652	species	*Rhopalosiphum insertum*	Aphididae	Hemiptera	15
345560	species	*Aphis rumicis*	Aphididae	Hemiptera	13
511039	species	*Aphis neospiraeae*	Aphididae	Hemiptera	12
69196	species	*Brevicoryne brassicae*	Aphididae	Hemiptera	11
1421473	species	*Cinara pectinatae*	Aphididae	Hemiptera	10
464946	genus	*Aphis*	Aphididae	Hemiptera	9
464927	species	*Aphis odinae*	Aphididae	Hemiptera	9
476189	species	*Rhopalosiphum musae*	Aphididae	Hemiptera	7
1350445	species	*Dermaphis crematogastri*	Hormaphididae	Hemiptera	4
384450	species	*Aleurodaphis mikaniae*	Hormaphididae	Hemiptera	3
97051	genus	*Cinara*	Aphididae	Hemiptera	2
866753	species	*Aulacorthum corydalicola*	Aphididae	Hemiptera	2
527681	species	*Eucallipterus tiliae*	Aphididae	Hemiptera	2
511041	species	*Aphis sanguisorbicola*	Aphididae	Hemiptera	2
469901	species	*Megoura brevipilosa*	Aphididae	Hemiptera	2
464926	species	*Aphis aurantii*	Aphididae	Hemiptera	2
387115	subfamily	Fordinae	Aphididae	Hemiptera	2
12998	genus	*Chaitophorus*	Aphididae	Hemiptera	2
1088304	species	*Theretra oldenlandiae*	Sphingidae	Lepidoptera	2
991824	species	*Aphis monardae*	Aphididae	Hemiptera	1
915061	species	*Uroleucon leonardi*	Aphididae	Hemiptera	1
758717	species	*Acleris sparsana*	Tortricidae	Lepidoptera	1
685722	species	*Aulacorthum cirsicola*	Aphididae	Hemiptera	1
511038	species	*Aphis kurosawai*	Aphididae	Hemiptera	1
506605	species	*Cinara maghrebica*	Aphididae	Hemiptera	1
486037	species	*Anuraphis pyrilaseri*	Aphididae	Hemiptera	1
469903	species	*Megoura lespedezae*	Aphididae	Hemiptera	1
464930	subgenus	*Bursaphis*	Aphididae	Hemiptera	1
419089	species	*Pineus similis*	Aphididae	Hemiptera	1
40931	genus	*Rhopalosiphum*	Aphididae	Hemiptera	1
384387	species	*Cinara formosana*	Aphididae	Hemiptera	1
384385	species	*Macrosiphoniella yomogifoliae*	Aphididae	Hemiptera	1
36997	species	*Ceratoglyphina styracicola*	Hormaphididae	Hemiptera	1

	30161	family	Hormaphididae	Hormaphididae	Hemiptera	1
	2768670	species	*Paurocephala sauteri*	Liviidae	Hemiptera	1
	2704473	species	*Macropodaphis paradoxa*	Aphididae	Hemiptera	1
	2082423	species	*Ourapteryx adonidaria*	Geometridae	Lepidoptera	1
	1925532	species	*Cinara sp. 3415*	Aphididae	Hemiptera	1
	168653	species	*Rhopalosiphum cerasifoliae*	Aphididae	Hemiptera	1
	1571514	species	*Gynaephora jiuzhiensis*	Erebidae	Lepidoptera	1
	1213610	species	*Galerucinae sp. 846596*	Chrysomelidae	Coleoptera	1
HC_5	464929	subgenus	*Aphis*	Aphididae	Hemiptera	85182
	384387	species	*Cinara formosana*	Aphididae	Hemiptera	14519
	13164	species	*Myzus persicae*	Aphididae	Hemiptera	6430
	511022	species	*Lipaphis pseudobrassicae*	Aphididae	Hemiptera	2053
	7029	species	*Acyrthosiphon pisum*	Aphididae	Hemiptera	1514
	486035	species	*Macrosiphum sp. C1774*	Aphididae	Hemiptera	1097
	80764	genus	*Aphis*	Aphididae	Hemiptera	1028
	33386	tribe	Macrosiphini	Aphididae	Hemiptera	789
	133076	subfamily	Aphidinae	Aphididae	Hemiptera	327
	69196	species	*Brevicoryne brassicae*	Aphididae	Hemiptera	303
	469905	species	*Megoura nigra*	Aphididae	Hemiptera	293
	330460	species	*Hyperomyzus lactucae*	Aphididae	Hemiptera	269
	1276923	species	*Hyalopterus amygdali*	Aphididae	Hemiptera	262
	224527	species	*Aphis spiraecola*	Aphididae	Hemiptera	214
	80765	species	*Aphis gossypii*	Aphididae	Hemiptera	161
	198323	species	*Cinara tujafilina*	Aphididae	Hemiptera	158
	51655	species	*Plutella xylostella*	Plutellidae	Lepidoptera	152
	1421473	species	*Cinara pectinatae*	Aphididae	Hemiptera	123
	527763	species	*Myzocallis coryli*	Aphididae	Hemiptera	97
	44664	species	*Sitobion avenae*	Aphididae	Hemiptera	88
	486031	species	*Brachycaudus sedi*	Aphididae	Hemiptera	76
	27482	family	Aphididae	Aphididae	Hemiptera	76
	33387	tribe	Aphidini	Aphididae	Hemiptera	60
	312888	species	*Hyalopterus pruni*	Aphididae	Hemiptera	56
	400569	species	*Aphis nasturtii*	Aphididae	Hemiptera	33
	97051	genus	*Cinara*	Aphididae	Hemiptera	32
	1078272	species	*Schizolachnus orientalis*	Aphididae	Hemiptera	27
	12998	genus	*Chaitophorus*	Aphididae	Hemiptera	26
	387115	subfamily	Fordinae	Aphididae	Hemiptera	20
	384450	species	*Aleurodaphis mikaniae*	Aphididae	Hemiptera	17
	168652	species	*Rhopalosiphum insertum*	Aphididae	Hemiptera	16
	1571514	species	*Gynaephora jiuzhiensis*	Erebidae	Lepidoptera	16
	702717	species	*Tuta absoluta*	Gelechiidae	Lepidoptera	15
	345560	species	*Aphis rumicis*	Aphididae	Hemiptera	9
	2024751	species	*Mahasena oolona*	Psychidae	Lepidoptera	9
	506608	species	*Cinara cedri*	Aphididae	Hemiptera	8
	30161	family	Hormaphididae	Hormaphididae	Hemiptera	7
	1421480	species	*Cinara wahluca*	Aphididae	Hemiptera	6
	464930	subgenus	*Bursaphis*	Aphididae	Hemiptera	5
	42295	genus	*Colias*	Pieridae	Lepidoptera	3
	40931	genus	*Rhopalosiphum*	Aphididae	Hemiptera	3
	2020618	subspecies	*Chaitophorus saliapterus quinquemaculatus*	Aphididae	Hemiptera	3

	476189	species	*Rhopalosiphum musae*	Aphididae	Hemiptera	2
	1244018	species	*Eulachnus alticola*	Aphididae	Hemiptera	2
	1209556	species	*Nomophila noctuella*	Crambidae	Lepidoptera	2
	910915	species	*Aphidura bozhkoae*	Aphididae	Hemiptera	1
	866753	species	*Aulacorthum corydalicola*	Aphididae	Hemiptera	1
	758717	species	*Acleris sparsana*	Tortricidae	Lepidoptera	1
	685722	species	*Aulacorthum cirsicola*	Aphididae	Hemiptera	1
	511041	species	*Aphis sanguisorbicola*	Aphididae	Hemiptera	1
	511038	species	*Aphis kurosawai*	Aphididae	Hemiptera	1
	220609	tribe	Cerataphidini	Aphididae	Hemiptera	1
	1497352	species	*Cinara aff. coloradensis 3048*	Aphididae	Hemiptera	1
	1350445	species	*Dermaphis crematogastri*	Aphididae	Hemiptera	1
	1242752	species	*Glyphodes pyloalis*	Crambidae	Lepidoptera	1
	1005069	species	*Adelges kitamiensis*	Adelgidae	Hemiptera	1
HC_6	464929	subgenus	*Aphis*	Aphididae	Hemiptera	111704
	1276923	species	*Hyalopterus amygdali*	Aphididae	Hemiptera	26667
	7029	species	*Acyrthosiphon pisum*	Aphididae	Hemiptera	7640
	13164	species	*Myzus persicae*	Aphididae	Hemiptera	3523
	44664	species	*Sitobion avenae*	Aphididae	Hemiptera	2834
	33386	tribe	Macrosiphini	Aphididae	Hemiptera	1730
	80764	genus	*Aphis*	Aphididae	Hemiptera	1015
	224527	species	*Aphis spiraecola*	Aphididae	Hemiptera	613
	469905	species	*Megoura nigra*	Aphididae	Hemiptera	164
	27482	family	Aphididae	Aphididae	Hemiptera	111
	80765	species	*Aphis gossypii*	Aphididae	Hemiptera	73
	133076	subfamily	Aphidinae	Aphididae	Hemiptera	70
	2020618	subspecies	Chaitophorus saliapterus quinquemaculatus	Aphididae	Hemiptera	69
	33387	tribe	Aphidini	Aphididae	Hemiptera	42
	168652	species	*Rhopalosiphum insertum*	Aphididae	Hemiptera	32
	1134961	subspecies	*Morpho sulkowskyi calderoni*	Nymphalidae	Lepidoptera	6
	1078272	species	*Schizolachnus orientalis*	Aphididae	Hemiptera	6
	464930	subgenus	*Bursaphis*	Aphididae	Hemiptera	5
	685722	species	*Aulacorthum cirsicola*	Aphididae	Hemiptera	4
	866753	species	*Aulacorthum corydalicola*	Aphididae	Hemiptera	2
	476189	species	*Rhopalosiphum musae*	Aphididae	Hemiptera	2
	511041	species	*Aphis sanguisorbicola*	Aphididae	Hemiptera	1
	511030	species	*Aphis crinosa*	Aphididae	Hemiptera	1
	486035	species	*Macrosiphum sp. C1774*	Aphididae	Hemiptera	1
	384465	species	*Ceratovacuna panici*	Aphididae	Hemiptera	1
	224525	genus	*Brachycaudus*	Aphididae	Hemiptera	1
HC_7	464929	subgenus	*Aphis*	Aphididae	Hemiptera	141900
	13164	species	*Myzus persicae*	Aphididae	Hemiptera	4256
	7029	species	*Acyrthosiphon pisum*	Aphididae	Hemiptera	3644
	80764	genus	*Aphis*	Aphididae	Hemiptera	2932
	1276923	species	*Hyalopterus amygdali*	Aphididae	Hemiptera	1094
	224527	species	*Aphis spiraecola*	Aphididae	Hemiptera	485
	486035	species	*Macrosiphum sp. C1774*	Aphididae	Hemiptera	471
	33386	tribe	Macrosiphini	Aphididae	Hemiptera	357
	44664	species	*Sitobion avenae*	Aphididae	Hemiptera	276
	1244010	species	*Cinara laricis*	Aphididae	Hemiptera	250

	511022	species	*Lipaphis pseudobrassicae*	Aphididae	Hemiptera	238
	69196	species	*Brevicoryne brassicae*	Aphididae	Hemiptera	198
	202396	species	*Rhopalosiphum padi*	Aphididae	Hemiptera	180
	80765	species	*Aphis gossypii*	Aphididae	Hemiptera	157
	133076	subfamily	Aphidinae	Aphididae	Hemiptera	141
	935269	species	*Therioaphis trifolii*	Aphididae	Hemiptera	137
	97078	species	*Stomaphis yanonis*	Aphididae	Hemiptera	125
	464926	species	*Aphis aurantii*	Aphididae	Hemiptera	106
	33387	tribe	Aphidini	Aphididae	Hemiptera	103
	400569	species	*Aphis nasturtii*	Aphididae	Hemiptera	94
	2572971	subfamily	Greenideinae	Aphididae	Hemiptera	81
	27482	family	Aphididae	Aphididae	Hemiptera	67
	486031	species	*Brachycaudus sedi*	Aphididae	Hemiptera	65
	30182	genus	*Pseudoregma*	Aphididae	Hemiptera	43
	2024751	species	*Mahasena oolona*	Psychidae	Lepidoptera	38
	464946	genus	*Aphis*	Aphididae	Hemiptera	31
	168652	species	*Rhopalosiphum insertum*	Aphididae	Hemiptera	13
	511041	species	*Aphis sanguisorbicola*	Aphididae	Hemiptera	12
	96541	subfamily	Lachninae	Aphididae	Hemiptera	9
	464930	subgenus	*Bursaphis*	Aphididae	Hemiptera	9
	1078272	species	*Schizolachnus orientalis*	Aphididae	Hemiptera	7
	1925539	species	*Cinara nr. spiculosa 3417*	Aphididae	Hemiptera	5
	97051	genus	*Cinara*	Aphididae	Hemiptera	4
	2082423	species	*Ourapteryx adonidaria*	Geometridae	Lepidoptera	4
	476189	species	*Rhopalosiphum musae*	Aphididae	Hemiptera	3
	1005069	species	*Adelges kitamiensis*	Adelgidae	Hemiptera	3
	76216	genus	*Lethe*	Nymphalidae	Lepidoptera	2
	51655	species	*Plutella xylostella*	Plutellidae	Lepidoptera	2
	42291	genus	*Parnassius*	Papilionidae	Lepidoptera	2
	384385	species	*Macrosiphoniella yomogifoliae*	Aphididae	Hemiptera	2
	2027753	species	*Periphyllus sp. ZMIOZ 26370*	Aphididae	Hemiptera	2
	12998	genus	*Chaitophorus*	Aphididae	Hemiptera	2
	1064604	species	*Stomaphis sinisalicis*	Aphididae	Hemiptera	2
	87309	genus	*Uroleucon*	Aphididae	Hemiptera	1
	511039	species	*Aphis neospiraeae*	Aphididae	Hemiptera	1
	511038	species	*Aphis kurosawai*	Aphididae	Hemiptera	1
	486027	species	*Brachycaudus prunicola*	Aphididae	Hemiptera	1
	40931	genus	*Rhopalosiphum*	Aphididae	Hemiptera	1
	345560	species	*Aphis rumicis*	Aphididae	Hemiptera	1
	2778777	species	*Tuberaphis sp. 3 TTX-2019*	Aphididae	Hemiptera	1
	220609	tribe	Cerataphidini	Aphididae	Hemiptera	1
	2027759	species	*Sipha (Rungsia) sp. ZMIOZ 17956*	Aphididae	Hemiptera	1
	2027655	species	*Sipha burakowskii*	Aphididae	Hemiptera	1
	2020618	subspecies	*Chaitophorus saliapterus quinquemaculatus*	Aphididae	Hemiptera	1
	1925532	species	*Cinara sp. 3415*	Aphididae	Hemiptera	1
	1421480	species	*Cinara wahluca*	Aphididae	Hemiptera	1
	13262	species	*Schizaphis graminum*	Aphididae	Hemiptera	1
HC_8	464929	subgenus	*Aphis*	Aphididae	Hemiptera	132545
	13164	species	*Myzus persicae*	Aphididae	Hemiptera	4479
	80764	genus	*Aphis*	Aphididae	Hemiptera	4351

486035	species	*Macrosiphum sp. C1774*	Aphididae	Hemiptera	2637
33386	tribe	Macrosiphini	Aphididae	Hemiptera	1314
7029	species	*Acyrthosiphon pisum*	Aphididae	Hemiptera	1132
224527	species	*Aphis spiraecola*	Aphididae	Hemiptera	645
27482	family	Aphididae	Aphididae	Hemiptera	428
44664	species	*Sitobion avenae*	Aphididae	Hemiptera	373
464926	species	*Aphis aurantii*	Aphididae	Hemiptera	315
33385	superfamily	Aphidoidea	Aphididae	Hemiptera	224
330460	species	*Hyperomyzus lactucae*	Aphididae	Hemiptera	174
133076	subfamily	Aphidinae	Aphididae	Hemiptera	154
33387	tribe	Aphidini	Aphididae	Hemiptera	110
202396	species	*Rhopalosiphum padi*	Aphididae	Hemiptera	105
69196	species	*Brevicoryne brassicae*	Aphididae	Hemiptera	104
80765	species	*Aphis gossypii*	Aphididae	Hemiptera	101
312888	species	*Hyalopterus pruni*	Aphididae	Hemiptera	58
1078272	species	*Schizolachnus orientalis*	Aphididae	Hemiptera	42
400569	species	*Aphis nasturtii*	Aphididae	Hemiptera	28
2024751	species	*Mahasena oolona*	Psychidae	Lepidoptera	21
2020618	subspecies	*Chaitophorus saliapterus quinquemaculatus*	Aphididae	Hemiptera	10
2082423	species	*Ourapteryx adonidaria*	Geometridae	Lepidoptera	9
469905	species	*Megoura nigra*	Aphididae	Hemiptera	8
33415	family	Nymphalidae	Nymphalidae	Lepidoptera	5
464946	genus	*Aphis*	Aphididae	Hemiptera	4
1667255	species	*Macropodaphis sp. YW-2015*	Aphididae	Hemiptera	4
511041	species	*Aphis sanguisorbicola*	Aphididae	Hemiptera	3
486023	species	*Brachycaudus malvae*	Aphididae	Hemiptera	3
476189	species	*Rhopalosiphum musae*	Aphididae	Hemiptera	3
464930	subgenus	*Bursaphis*	Aphididae	Hemiptera	3
991824	species	*Aphis monardae*	Aphididae	Hemiptera	2
511038	species	*Aphis kurosawai*	Aphididae	Hemiptera	2
345567	species	*Aphis ulmariae*	Aphididae	Hemiptera	2
198323	species	*Cinara tujafilina*	Aphididae	Hemiptera	2
133082	species	*Eriosoma lanigerum*	Aphididae	Hemiptera	2
13262	species	*Schizaphis graminum*	Aphididae	Hemiptera	2
1134961	subspecies	*Morpho sulkowskyi calderoni*	Nymphalidae	Lepidoptera	2
1005069	species	*Adelges kitamiensis*	Adelgidae	Hemiptera	2
910915	species	*Aphidura bozhkoae*	Aphididae	Hemiptera	1
796220	species	*Aulacorthum vandenboschi*	Aphididae	Hemiptera	1
7137	species	*Galleria mellonella*	Pyralidae	Lepidoptera	1
685722	species	*Aulacorthum cirsicola*	Aphididae	Hemiptera	1
685721	species	*Aulacorthum albimagnoliae*	Aphididae	Hemiptera	1
511039	species	*Aphis neospiraeae*	Aphididae	Hemiptera	1
511036	species	*Aphis ichigo*	Aphididae	Hemiptera	1
419084	species	*Pineus armandicola*	Adelgidae	Hemiptera	1
345560	species	*Aphis rumicis*	Aphididae	Hemiptera	1
323675	species	*Eurema blanda*	Pieridae	Lepidoptera	1
2638729	genus	*Cinara*	Aphididae	Hemiptera	1
168652	species	*Rhopalosiphum insertum*	Aphididae	Hemiptera	1
1350445	species	*Dermaphis crematogastri*	Aphididae	Hemiptera	1
1078808	species	*Sphinx morio*	Sphingidae	Lepidoptera	1

HC_9	464929	subgenus	*Aphis*	Aphididae	Hemiptera	65066
	469905	species	*Megoura nigra*	Aphididae	Hemiptera	7724
	13164	species	*Myzus persicae*	Aphididae	Hemiptera	3043
	1276923	species	*Hyalopterus amygdali*	Aphididae	Hemiptera	2119
	7029	species	*Acyrthosiphon pisum*	Aphididae	Hemiptera	2018
	486035	species	*Macrosiphum sp. C1774*	Aphididae	Hemiptera	1917
	80764	genus	*Aphis*	Aphididae	Hemiptera	1053
	33386	tribe	Macrosiphini	Aphididae	Hemiptera	1006
	158624	species	*Propylea japonica*	Coccinellidae	Coleoptera	385
	27482	family	Aphididae	Aphididae	Hemiptera	364
	44664	species	*Sitobion avenae*	Aphididae	Hemiptera	331
	330460	species	*Hyperomyzus lactucae*	Aphididae	Hemiptera	325
	224527	species	*Aphis spiraecola*	Aphididae	Hemiptera	238
	1078272	species	*Schizolachnus orientalis*	Aphididae	Hemiptera	120
	1421473	species	*Cinara pectinatae*	Aphididae	Hemiptera	111
	202396	species	*Rhopalosiphum padi*	Aphididae	Hemiptera	90
	133076	subfamily	Aphidinae	Aphididae	Hemiptera	87
	464926	species	*Aphis aurantii*	Aphididae	Hemiptera	82
	80765	species	*Aphis gossypii*	Aphididae	Hemiptera	41
	33387	tribe	Aphidini	Aphididae	Hemiptera	38
	2020618	subspecies	*Chaitophorus saliapterus quinquemaculatus*	Aphididae	Hemiptera	26
	41905	species	*Ceratovacuna lanigera*	Hormaphididae	Hemiptera	23
	97078	species	*Stomaphis yanonis*	Aphididae	Hemiptera	19
	2024751	species	*Mahasena oolona*	Psychidae	Lepidoptera	18
	30182	genus	*Pseudoregma*	Aphididae	Hemiptera	9
	198323	species	*Cinara tujafilina*	Aphididae	Hemiptera	8
	511022	species	*Lipaphis pseudobrassicae*	Aphididae	Hemiptera	6
	345560	species	*Aphis rumicis*	Aphididae	Hemiptera	6
	311130	species	*Salamis anteva*	Nymphalidae	Lepidoptera	4
	1209556	species	*Nomophila noctuella*	Crambidae	Lepidoptera	4
	1925532	species	*Cinara sp. 3415*	Aphididae	Hemiptera	3
	168652	species	*Rhopalosiphum insertum*	Aphididae	Hemiptera	3
	12998	genus	*Chaitophorus*	Aphididae	Hemiptera	2
	97051	genus	*Cinara*	Aphididae	Hemiptera	1
	935663	species	*Phyllaphis fagi*	Aphididae	Hemiptera	1
	910915	species	*Aphidura bozhkoae*	Aphididae	Hemiptera	1
	866753	species	*Aulacorthum corydalicola*	Aphididae	Hemiptera	1
	758717	species	*Acleris sparsana*	Tortricidae	Lepidoptera	1
	7137	species	*Galleria mellonella*	Pyralidae	Lepidoptera	1
	511039	species	*Aphis neospiraeae*	Aphididae	Hemiptera	1
	511030	species	*Aphis crinosa*	Aphididae	Hemiptera	1
	476190	species	*Rhopalosiphum sp. X IL-2007*	Aphididae	Hemiptera	1
	476189	species	*Rhopalosiphum musae*	Aphididae	Hemiptera	1
	469903	species	*Megoura lespedezae*	Aphididae	Hemiptera	1
	387115	subfamily	Fordinae	Aphididae	Hemiptera	1
	215788	tribe	Limenitidini	Nymphalidae	Lepidoptera	1
	168631	species	*Chilo suppressalis*	Crambidae	Lepidoptera	1
	1350441	species	*Nipponaphis sp. JC-2013*	Aphididae	Hemiptera	1
	1309588	species	*Cydalima perspectalis*	Crambidae	Lepidoptera	1
HC_10	464929	subgenus	*Aphis*	Aphididae	Hemiptera	50929

	224527	species	*Aphis spiraecola*	Aphididae	Hemiptera	1658
	80764	genus	*Aphis*	Aphididae	Hemiptera	937
	13164	species	*Myzus persicae*	Aphididae	Hemiptera	781
	1276923	species	*Hyalopterus amygdali*	Aphididae	Hemiptera	442
	202396	species	*Rhopalosiphum padi*	Aphididae	Hemiptera	375
	33386	tribe	Macrosiphini	Aphididae	Hemiptera	147
	511022	species	*Lipaphis pseudobrassicae*	Aphididae	Hemiptera	106
	488727	species	*Astegopteryx styracophila*	Aphididae	Hemiptera	97
	758717	species	*Acleris sparsana*	Tortricidae	Lepidoptera	76
	44664	species	*Sitobion avenae*	Aphididae	Hemiptera	69
	80765	species	*Aphis gossypii*	Aphididae	Hemiptera	60
	27482	family	Aphididae	Aphididae	Hemiptera	59
	133076	subfamily	Aphidinae	Aphididae	Hemiptera	59
	69196	species	*Brevicoryne brassicae*	Aphididae	Hemiptera	53
	7029	species	*Acyrthosiphon pisum*	Aphididae	Hemiptera	43
	527763	species	*Myzocallis coryli*	Aphididae	Hemiptera	41
	542820	species	*Chromaphis juglandicola*	Aphididae	Hemiptera	22
	486035	species	*Macrosiphum sp. C1774*	Aphididae	Hemiptera	18
	33387	tribe	Aphidini	Aphididae	Hemiptera	16
	486031	species	*Brachycaudus sedi*	Aphididae	Hemiptera	14
	312888	species	*Hyalopterus pruni*	Aphididae	Hemiptera	14
	214277	species	*Noctua pronuba*	Noctuidae	Lepidoptera	8
	400569	species	*Aphis nasturtii*	Aphididae	Hemiptera	7
	168652	species	*Rhopalosiphum insertum*	Aphididae	Hemiptera	4
	12998	genus	*Chaitophorus*	Aphididae	Hemiptera	3
	476189	species	*Rhopalosiphum musae*	Aphididae	Hemiptera	2
	1785073	species	*Periphyllus acerihabitans*	Aphididae	Hemiptera	2
	1350445	species	*Dermaphis crematogastri*	Aphididae	Hemiptera	2
	1209556	species	*Nomophila noctuella*	Crambidae	Lepidoptera	2
	1078272	species	*Schizolachnus orientalis*	Aphididae	Hemiptera	2
	991824	species	*Aphis monardae*	Aphididae	Hemiptera	1
	97051	genus	*Cinara*	Aphididae	Hemiptera	1
	464946	genus	*Aphis*	Aphididae	Hemiptera	1
	40931	genus	*Rhopalosiphum*	Aphididae	Hemiptera	1
	33415	family	Nymphalidae	Nymphalidae	Lepidoptera	1
	2704477	species	*Yamatocallis sauteri*	Aphididae	Hemiptera	1
	2082423	species	*Ourapteryx adonidaria*	Geometridae	Lepidoptera	1
	1904465	species	*Hybothoracaphis laevigata*	Aphididae	Hemiptera	1
	1265466	species	*Epipemphigus yunnanensis*	Pemphigidae	Hemiptera	1
HC_11	464929	subgenus	*Aphis*	Aphididae	Hemiptera	115078
	13164	species	*Myzus persicae*	Aphididae	Hemiptera	3959
	1276923	species	*Hyalopterus amygdali*	Aphididae	Hemiptera	2675
	7029	species	*Acyrthosiphon pisum*	Aphididae	Hemiptera	2568
	224527	species	*Aphis spiraecola*	Aphididae	Hemiptera	2290
	488727	species	*Astegopteryx styracophila*	Hormaphididae	Hemiptera	2198
	80764	genus	*Aphis*	Aphididae	Hemiptera	1890
	486035	species	*Macrosiphum sp. C1774*	Aphididae	Hemiptera	512
	469905	species	*Megoura nigra*	Aphididae	Hemiptera	396
	133076	subfamily	Aphidinae	Aphididae	Hemiptera	348
	33386	tribe	Macrosiphini	Aphididae	Hemiptera	260

	312888	species	*Hyalopterus pruni*	Aphididae	Hemiptera	163
	202396	species	*Rhopalosiphum padi*	Aphididae	Hemiptera	128
	33387	tribe	Aphidini	Aphididae	Hemiptera	118
	2704477	species	*Rhopalosiphum padi*	Aphididae	Hemiptera	93
	1209556	species	*Nomophila noctuella*	Crambidae	Lepidoptera	91
	80765	species	*Aphis gossypii*	Aphididae	Hemiptera	89
	12998	genus	*Chaitophorus*	Aphididae	Hemiptera	72
	27482	family	Aphididae	Aphididae	Hemiptera	55
	1350445	species	*Dermaphis crematogastri*	Hormaphididae	Hemiptera	47
	76195	species	*Papilio helenus*	Papilionidae	Lepidoptera	36
	464926	species	*Aphis aurantii*	Aphididae	Hemiptera	22
	1785073	species	*Periphyllus acerihabitans*	Aphididae	Hemiptera	19
	511022	species	*Lipaphis pseudobrassicae*	Aphididae	Hemiptera	18
	330460	species	*Hyperomyzus lactucae*	Aphididae	Hemiptera	15
	1078272	species	*Schizolachnus orientalis*	Aphididae	Hemiptera	13
	97078	species	*Stomaphis yanonis*	Aphididae	Hemiptera	9
	30182	genus	*Pseudoregma*	Hormaphididae	Hemiptera	8
	464930	subgenus	*Bursaphis*	Aphididae	Hemiptera	6
	168652	species	*Rhopalosiphum insertum*	Aphididae	Hemiptera	6
	511041	species	*Aphis sanguisorbicola*	Aphididae	Hemiptera	5
	97051	genus	*Cinara*	Aphididae	Hemiptera	3
	476189	species	*Rhopalosiphum musae*	Aphididae	Hemiptera	2
	345560	species	*Aphis rumicis*	Aphididae	Hemiptera	2
	1244010	species	*Cinara laricis*	Aphididae	Hemiptera	2
	1049572	species	*Mollitrichosiphum tenuicorpus*	Aphididae	Hemiptera	2
	96541	subfamily	Lachninae	Aphididae	Hemiptera	1
	7137	species	*Galleria mellonella*	Pyralidae	Lepidoptera	1
	527681	species	*Eucallipterus tiliae*	Aphididae	Hemiptera	1
	51655	species	*Plutella xylostella*	Plutellidae	Lepidoptera	1
	511039	species	*Aphis neospiraeae*	Aphididae	Hemiptera	1
	511038	species	*Aphis kurosawai*	Aphididae	Hemiptera	1
	464946	genus	*Aphis*	Aphididae	Hemiptera	1
	419089	species	*Pineus similis*	Adelgidae	Hemiptera	1
	384387	species	*Cinara formosana*	Aphididae	Hemiptera	1
	384385	species	*Macrosiphoniella yomogifoliae*	Aphididae	Hemiptera	1
	384373	species	*Cervaphis quercus*	Aphididae	Hemiptera	1
	2020618	subspecies	*Chaitophorus saliapterus quinquemaculatus*	Aphididae	Hemiptera	1
	1870435	species	*Ypsolopha scabrella*	Ypsolophidae	Lepidoptera	1
	133097	genus	*Periphyllus*	Aphididae	Hemiptera	1
HC_12	464929	subgenus	*Aphis*	Aphididae	Hemiptera	42845
	1276923	species	*Hyalopterus amygdali*	Aphididae	Hemiptera	2446
	13164	species	*Myzus persicae*	Aphididae	Hemiptera	917
	33386	tribe	Macrosiphini	Aphididae	Hemiptera	803
	224527	species	*Aphis spiraecola*	Aphididae	Hemiptera	727
	80764	genus	*Aphis*	Aphididae	Hemiptera	695
	469905	species	*Megoura nigra*	Aphididae	Hemiptera	215
	511022	species	*Lipaphis pseudobrassicae*	Aphididae	Hemiptera	173
	542820	species	*Chromaphis juglandicola*	Aphididae	Hemiptera	112
	486035	species	*Macrosiphum sp. C1774*	Aphididae	Hemiptera	96
	7029	species	*Acyrthosiphon pisum*	Aphididae	Hemiptera	84

	224525	genus	*Brachycaudus*	Aphididae	Hemiptera	83
	133076	subfamily	Aphidinae	Aphididae	Hemiptera	72
	27482	family	Aphididae	Aphididae	Hemiptera	71
	69196	species	*Brevicoryne brassicae*	Aphididae	Hemiptera	61
	80765	species	*Aphis gossypii*	Aphididae	Hemiptera	41
	202396	species	*Rhopalosiphum padi complex*	Aphididae	Hemiptera	40
	1078272	species	*Schizolachnus orientalis*	Aphididae	Hemiptera	35
	44664	species	*Sitobion avenae*	Aphididae	Hemiptera	32
	464946	genus	*Aphis*	Aphididae	Hemiptera	31
	13262	species	*Schizaphis graminum*	Aphididae	Hemiptera	26
	384385	species	*Macrosiphoniella yomogifoliae*	Aphididae	Hemiptera	20
	33387	tribe	Aphidini	Aphididae	Hemiptera	15
	400569	species	*Aphis nasturtii*	Aphididae	Hemiptera	11
	51655	species	*Plutella xylostella*	Plutellidae	Lepidoptera	9
	330460	species	*Hyperomyzus lactucae*	Aphididae	Hemiptera	9
	12998	genus	*Chaitophorus*	Aphididae	Hemiptera	6
	168652	species	*Rhopalosiphum insertum*	Aphididae	Hemiptera	5
	1209556	species	*Nomophila noctuella*	Crambidae	Lepidoptera	5
	866753	species	*Aulacorthum corydalicola*	Aphididae	Hemiptera	3
	511041	species	*Aphis sanguisorbicola*	Aphididae	Hemiptera	2
	97051	genus	*Cinara*	Aphididae	Hemiptera	1
	910915	species	*Aphidura bozhkoae*	Aphididae	Hemiptera	1
	796220	species	*Aulacorthum vandenboschi*	Aphididae	Hemiptera	1
	511039	species	*Aphis neospiraeae*	Aphididae	Hemiptera	1
	486023	species	*Brachycaudus malvae*	Aphididae	Hemiptera	1
	476189	species	*Rhopalosiphum musae*	Aphididae	Hemiptera	1
	419070	species	*Phylloxera sp. CNC-HEM017791*	Phylloxeridae	Hemiptera	1
	2020618	subspecies	*Chaitophorus saliapterus quinquemaculatus*	Aphididae	Hemiptera	1
	198323	species	*Cinara tujafilina*	Aphididae	Hemiptera	1
	1005069	species	*Adelges kitamiensis*	Phylloxeridae	Hemiptera	1
HB_1A1	13164	species	*Myzus persicae*	Aphididae	Hemiptera	6493
	7029	species	*Acyrthosiphon pisum*	Aphididae	Hemiptera	3140
	464929	subgenus	*Aphis*	Aphididae	Hemiptera	1175
	935269	species	*Therioaphis trifolii*	Aphididae	Hemiptera	738
	198323	species	*Cinara tujafilina*	Aphididae	Hemiptera	700
	12998	genus	*Chaitophorus*	Aphididae	Hemiptera	522
	13262	species	*Schizaphis graminum*	Aphididae	Hemiptera	433
	44664	species	*Sitobion avenae*	Aphididae	Hemiptera	356
	469905	species	*Megoura nigra*	Aphididae	Hemiptera	184
	133076	subfamily	*Aphidinae*	Aphididae	Hemiptera	137
	345571	genus	*Melanaphis*	Aphididae	Hemiptera	99
	486035	species	*Macrosiphum sp. C1774*	Aphididae	Hemiptera	92
	69196	species	*Brevicoryne brassicae*	Aphididae	Hemiptera	79
	80764	genus	*Aphis*	Aphididae	Hemiptera	18
	202396	species	*Rhopalosiphum padi complex*	Aphididae	Hemiptera	12
	27482	family	Aphididae	Aphididae	Hemiptera	11
	384450	species	*Aleurodaphis mikaniae*	Hormaphididae	Hemiptera	7
	33386	tribe	Macrosiphini	Aphididae	Hemiptera	5
	30161	family	Hormaphididae	Hormaphididae	Hemiptera	5
	1037414	family	Hepialidae	Hepialidae	Lepidoptera	3

	685721	species	*Aulacorthum albimagnoliae*	Aphididae	Hemiptera	1
	384386	species	*Cavariella salicicola*	Aphididae	Hemiptera	1
	2768670	species	*Paurocephala sauteri*	Liviidae	Hemiptera	1
	168631	species	*Chilo suppressalis*	Crambidae	Lepidoptera	1
	1242752	species	*Glyphodes pyloalis*	Crambidae	Lepidoptera	1
	1134961	subspecies	*Morpho sulkowskyi calderoni*	Nymphalidae	Lepidoptera	1
HB_1A2	935269	species	*Therioaphis trifolii*	Aphididae	Hemiptera	30115
	464929	subgenus	*Aphis*	Aphididae	Hemiptera	11398
	198318	species	*Tinocallis takachihoensis*	Aphididae	Hemiptera	1917
	13164	species	*Myzus persicae*	Aphididae	Hemiptera	1803
	12998	genus	*Chaitophorus*	Aphididae	Hemiptera	479
	96551	species	*Tuberolachnus salignus*	Aphididae	Hemiptera	337
	44664	species	*Sitobion avenae*	Aphididae	Hemiptera	211
	27482	family	Aphididae	Aphididae	Hemiptera	86
	133076	subfamily	Aphidinae	Aphididae	Hemiptera	35
	384387	species	*Cinara formosana*	Aphididae	Hemiptera	26
	384450	species	*Aleurodaphis mikaniae*	Hormaphididae	Hemiptera	17
	80764	genus	*Aphis*	Aphididae	Hemiptera	14
	30161	family	Hormaphididae	Hormaphididae	Hemiptera	8
	527648	species	*Drepanosiphum platanoidis*	Aphididae	Hemiptera	5
	51655	species	*Plutella xylostella*	Plutellidae	Lepidoptera	2
	253253	species	*Rhopalosiphum nymphaeae*	Aphididae	Hemiptera	2
	1037414	family	Hepialidae	Hepialidae	Lepidoptera	2
	100479	species	*Aphis nerii*	Aphididae	Hemiptera	2
	910915	species	*Aphidura bozhkoae*	Aphididae	Hemiptera	1
	41905	species	*Ceratovacuna lanigera*	Hormaphididae	Hemiptera	1
	384469	species	*Aleurodaphis asteris*	Hormaphididae	Hemiptera	1
	2768670	species	*Paurocephala sauteri*	Liviidae	Hemiptera	1
HB_1C1	133076	subfamily	Aphidinae	Aphididae	Hemiptera	3317
	13164	species	*Myzus persicae*	Aphididae	Hemiptera	2608
	13262	species	*Schizaphis graminum*	Aphididae	Hemiptera	1349
	7029	species	*Acyrthosiphon pisum*	Aphididae	Hemiptera	1257
	69196	species	*Brevicoryne brassicae*	Aphididae	Hemiptera	224
	935269	species	*Therioaphis trifolii*	Aphididae	Hemiptera	164
	464929	subgenus	*Aphis*	Aphididae	Hemiptera	120
	33386	tribe	Macrosiphini	Aphididae	Hemiptera	115
	12998	genus	*Chaitophorus*	Aphididae	Hemiptera	84
	44664	species	*Sitobion avenae*	Aphididae	Hemiptera	25
	27482	family	Aphididae	Aphididae	Hemiptera	21
	384450	species	*Aleurodaphis mikaniae*	Hormaphididae	Hemiptera	14
	511022	species	*Lipaphis pseudobrassicae*	Aphididae	Hemiptera	7
	469901	species	*Megoura brevipilosa*	Aphididae	Hemiptera	6
	30161	family	Hormaphididae	Hormaphididae	Hemiptera	6
	1425443	species	*Neotoxoptera formosana*	Aphididae	Hemiptera	5
	97051	genus	*Cinara*	Aphididae	Hemiptera	4
	506608	species	*Cinara cedri*	Aphididae	Hemiptera	3
	345560	species	*Aphis rumicis*	Aphididae	Hemiptera	3
	1037414	family	Hepialidae	Hepialidae	Lepidoptera	2
	80764	genus	*Aphis*	Aphididae	Hemiptera	1
	330460	species	*Hyperomyzus lactucae*	Aphididae	Hemiptera	1

	198323	species	*Cinara tujafilina*	Aphididae	Hemiptera	1
	1078272	species	*Schizolachnus orientalis*	Aphididae	Hemiptera	1
	100479	species	*Aphis nerii*	Aphididae	Hemiptera	1
HB_2A2	253253	species	*Rhopalosiphum nymphaeae*	Aphididae	Hemiptera	9651
	464929	subgenus	*Aphis*	Aphididae	Hemiptera	1040
	384450	species	*Rhopalus parumpunctatus*	Hormaphididae	Hemiptera	986
	30161	family	Hormaphididae	Hormaphididae	Hemiptera	406
	935269	species	*Therioaphis trifolii*	Aphididae	Hemiptera	274
	384387	species	*Cinara formosana*	Aphididae	Hemiptera	193
	158624	species	*Propylea japonica*	Coccinellidae	Coleoptera	186
	2020618	subspecies	*Chaitophorus saliapterus quinquemaculatus*	Aphididae	Hemiptera	122
	99934	species	*Lachnus roboris*	Aphididae	Hemiptera	93
	1276923	species	*Hyalopterus amygdali*	Aphididae	Hemiptera	91
	1037414	family	Hepialidae	Hepialidae	Lepidoptera	89
	506608	species	*Cinara cedri*	Aphididae	Hemiptera	42
	214277	species	*Noctua pronuba*	Noctuidae	Lepidoptera	32
	13262	species	*Schizaphis graminum*	Aphididae	Hemiptera	31
	2768670	species	*Paurocephala sauteri*	Liviidae	Hemiptera	30
	384469	species	*Aleurodaphis asteris*	Hormaphididae	Hemiptera	29
	7029	species	*Acyrthosiphon pisum*	Aphididae	Hemiptera	25
	27482	family	Aphididae	Aphididae	Hemiptera	17
	1078270	species	*Ceratovacuna silvestrii*	Hormaphididae	Hemiptera	15
	312888	species	*Hyalopterus pruni*	Aphididae	Hemiptera	10
	80764	genus	*Aphis*	Aphididae	Hemiptera	6
	51655	species	*Plutella xylostella*	Plutellidae	Lepidoptera	6
	97051	genus	*Cinara*	Aphididae	Hemiptera	5
	1071973	species	*Pterocomma rufipes*	Aphididae	Hemiptera	5
	133076	subfamily	Aphidinae	Aphididae	Hemiptera	4
	2778780	species	*Hamamelistes sp. NZMC aphid 37937*	Hormaphididae	Hemiptera	3
	915061	species	*Uroleucon leonardi*	Aphididae	Hemiptera	2
	405028	species	*Issoria eugenia*	Nymphalidae	Lepidoptera	2
	220609	tribe	Cerataphidini	Hormaphididae	Hemiptera	2
	96555	species	*Trama troglodytes*	Aphididae	Hemiptera	1
	76195	species	*Papilio helenus*	Papilionidae	Lepidoptera	1
	758717	species	*Acleris sparsana*	Tortricidae	Lepidoptera	1
	749403	species	*Dreyfusia piceae*	Adelgidae	Hemiptera	1
	486031	species	*Brachycaudus sedi*	Aphididae	Hemiptera	1
	384385	species	*Macrosiphoniella yomogifoliae*	Aphididae	Hemiptera	1
	36997	species	*Ceratoglyphina styracicola*	Hormaphididae	Hemiptera	1
	33415	family	Nymphalidae	Nymphalidae	Lepidoptera	1
	334015	species	*Coreana raphaelis*	Lycaenidae	Lepidoptera	1
	33387	tribe	Aphidini	Aphididae	Hemiptera	1
	30164	genus	*Astegopteryx*	Hormaphididae	Hemiptera	1
	262662	species	*Papilio rex*	Papilionidae	Lepidoptera	1
	224525	genus	*Brachycaudus*	Aphididae	Hemiptera	1
	171587	species	*Issoria lathonia*	Nymphalidae	Lepidoptera	1
	1481755	species	*Parasa consocia*	Limacodidae	Lepidoptera	1
	1421480	species	*Cinara wahluca*	Aphididae	Hemiptera	1
	1217617	species	*Ceratovacuna indica*	Hormaphididae	Hemiptera	1
	1137966	species	*Calyptra lata*	Erebidae	Lepidoptera	1

	100479	species	*Aphis nerii*	Aphididae	Hemiptera	1
HB_2B2	384450	species	*Rhopalus parumpunctatus*	Hormaphididae	Hemiptera	1298
	464929	subgenus	*Aphis*	Aphididae	Hemiptera	665
	30161	family	Hormaphididae	Hormaphididae	Hemiptera	462
	506608	species	*Cinara cedri*	Aphididae	Hemiptera	446
	253253	species	*Rhopalosiphum nymphaeae*	Aphididae	Hemiptera	308
	935269	species	*Therioaphis trifolii*	Aphididae	Hemiptera	222
	527681	species	*Eucallipterus tiliae*	Aphididae	Hemiptera	115
	1276923	species	*Hyalopterus amygdali*	Aphididae	Hemiptera	96
	1078272	species	*Schizolachnus orientalis*	Aphididae	Hemiptera	95
	384387	species	*Cinara formosana*	Aphididae	Hemiptera	92
	13164	species	*Myzus persicae*	Aphididae	Hemiptera	92
	1037414	family	Hepialidae	Hepialidae	Lepidoptera	82
	80764	genus	*Aphis*	Aphididae	Hemiptera	73
	464946	genus	*Aphis*	Aphididae	Hemiptera	59
	158624	species	Propylea japonica	Coccinellidae	Coleoptera	50
	2020618	subspecies	*Chaitophorus saliapterus quinquemaculatus*	Aphididae	Hemiptera	43
	7029	species	*Acyrthosiphon pisum*	Aphididae	Hemiptera	34
	2768670	species	*Paurocephala sauteri*	Liviidae	Hemiptera	28
	2572208	subspecies	*Meandrusa sciron aribbas*	Papilionidae	Lepidoptera	27
	214277	species	*Noctua pronuba*	Noctuidae	Lepidoptera	25
	12998	genus	*Chaitophorus*	Aphididae	Hemiptera	21
	254363	species	*Autographa gamma*	Noctuidae	Lepidoptera	17
	384469	species	*Aleurodaphis asteris*	Hormaphididae	Hemiptera	7
	27482	family	Aphididae	Aphididae	Hemiptera	7
	1281420	species	*Pyrausta despicata*	Crambidae	Lepidoptera	7
	1078270	species	*Ceratovacuna silvestrii*	Hormaphididae	Hemiptera	7
	1071973	species	*Pterocomma rufipes*	Aphididae	Hemiptera	7
	96555	species	*Trama troglodytes*	Aphididae	Hemiptera	4
	2778780	species	*Hamamelistes sp. NZMC aphid 37937*	Hormaphididae	Hemiptera	3
	99936	species	*Pterochloroides persicae*	Aphididae	Hemiptera	2
	97051	genus	*Cinara*	Aphididae	Hemiptera	2
	96541	subfamily	Lachninae	Aphididae	Hemiptera	2
	876379	species	*Cethosia biblis*	Nymphalidae	Lepidoptera	2
	38118	genus	*Adelges*	Adelgidae	Hemiptera	2
	312888	species	*Hyalopterus pruni*	Aphididae	Hemiptera	2
	133065	species	*Adelges cooleyi*	Adelgidae	Hemiptera	2
	749403	species	*Dreyfusia piceae*	Adelgidae	Hemiptera	1
	405028	species	*Issoria eugenia*	Nymphalidae	Lepidoptera	1
	357116	species	*Dioryctria yiai*	Pyralidae	Lepidoptera	1
	334015	species	*Coreana raphaelis*	Lycaenidae	Lepidoptera	1
	1712687	species	*Plocamaphis assetacea*	Aphididae	Hemiptera	1
	1706501	species	*Leucoma chrysoscela*	Erebidae	Lepidoptera	1
	1481755	species	*Parasa consocia*	Limacodidae	Lepidoptera	1
	1350457	species	*Reticulaphis sp. JC-2013*	Hormaphididae	Hemiptera	1
	133076	subfamily	Aphidinae	Aphididae	Hemiptera	1
HB_2C2	506608	species	*Cinara cedri*	Aphididae	Hemiptera	1297
	464929	subgenus	*Aphis*	Aphididae	Hemiptera	820
	384450	species	*Rhopalus parumpunctatus*	Hormaphididae	Hemiptera	425
	27482	family	Aphididae	Aphididae	Hemiptera	331

	935269	species	*Therioaphis trifolii*	Aphididae	Hemiptera	243
	254363	species	*Autographa gamma*	Noctuidae	Lepidoptera	204
	253253	species	*Rhopalosiphum nymphaeae*	Aphididae	Hemiptera	185
	384387	species	*Cinara formosana*	Aphididae	Hemiptera	111
	30161	family	Hormaphididae	Hormaphididae	Hemiptera	100
	312888	species	*Hyalopterus pruni*	Aphididae	Hemiptera	67
	7029	species	*Acyrthosiphon pisum*	Aphididae	Hemiptera	54
	44664	species	*Sitobion avenae*	Aphididae	Hemiptera	45
	1037414	family	Hepialidae	Hepialidae	Lepidoptera	28
	12998	genus	*Chaitophorus*	Aphididae	Hemiptera	22
	41905	species	*Ceratovacuna lanigera*	Hormaphididae	Hemiptera	6
	2768670	species	*Paurocephala sauteri*	Liviidae	Hemiptera	5
	1078270	species	*Ceratovacuna silvestrii*	Hormaphididae	Hemiptera	3
	7137	species	*Galleria mellonella*	Pyralidae	Lepidoptera	2
	384469	species	*Aleurodaphis asteris*	Hormaphididae	Hemiptera	2
	30162	genus	*Aleurodaphis*	Hormaphididae	Hemiptera	2
	2572208	subspecies	*Meandrusa sciron aribbas*	Papilionidae	Lepidoptera	2
	97051	genus	*Cinara*	Aphididae	Hemiptera	1
	875884	species	*Phlogophora meticulosa*	Noctuidae	Lepidoptera	1
	80764	genus	*Aphis*	Aphididae	Hemiptera	1
	405028	species	*Issoria eugenia*	Nymphalidae	Lepidoptera	1
	330460	species	*Hyperomyzus lactucae*	Aphididae	Hemiptera	1
	1481755	species	*Parasa consocia*	Limacodidae	Lepidoptera	1
HB_3A3	935269	species	*Therioaphis trifolii*	Aphididae	Hemiptera	10752
	464929	subgenus	*Aphis*	Aphididae	Hemiptera	8373
	13262	species	*Schizaphis graminum*	Aphididae	Hemiptera	1987
	13164	species	*Myzus persicae*	Aphididae	Hemiptera	1729
	527681	species	*Eucallipterus tiliae*	Aphididae	Hemiptera	574
	400569	species	*Aphis nasturtii*	Aphididae	Hemiptera	221
	80764	genus	*Aphis*	Aphididae	Hemiptera	204
	7029	species	*Acyrthosiphon pisum*	Aphididae	Hemiptera	185
	384450	species	*Rhopalus parumpunctatus*	Hormaphididae	Hemiptera	101
	30161	family	Hormaphididae	Hormaphididae	Hemiptera	83
	27482	family	Aphididae	Aphididae	Hemiptera	62
	988118	species	*Gortyna flavago*	Noctuidae	Lepidoptera	50
	133076	subfamily	Aphidinae	Aphididae	Hemiptera	46
	97051	genus	*Cinara*	Aphididae	Hemiptera	35
	384387	species	*Cinara formosana*	Aphididae	Hemiptera	18
	1037414	family	Hepialidae	Hepialidae	Lepidoptera	14
	1134961	subspecies	*Morpho sulkowskyi calderoni*	Nymphalidae	Lepidoptera	11
	2768670	species	*Paurocephala sauteri*	Liviidae	Hemiptera	4
	1078270	species	*Ceratovacuna silvestrii*	Hormaphididae	Hemiptera	4
	7137	species	*Galleria mellonella*	Pyralidae	Lepidoptera	3
	527605	species	*Cinara fornacula*	Aphididae	Hemiptera	3
	405028	species	*Issoria eugenia*	Nymphalidae	Lepidoptera	3
	33386	tribe	Macrosiphini	Aphididae	Hemiptera	3
	214277	species	*Noctua pronuba*	Noctuidae	Lepidoptera	3
	168631	species	*Chilo suppressalis*	Crambidae	Lepidoptera	3
	100479	species	*Aphis nerii*	Aphididae	Hemiptera	3
	73260	species	*Japonica lutea*	Lycaenidae	Lepidoptera	2

	41905	species	*Ceratovacuna lanigera*	Hormaphididae	Hemiptera	2
	99933	species	*Lachnus tropicalis*	Aphididae	Hemiptera	1
	753195	species	*Lyonetia clerkella*	Lyonetiidae	Lepidoptera	1
	511030	species	*Aphis crinosa*	Aphididae	Hemiptera	1
	506608	species	*Cinara cedri*	Aphididae	Hemiptera	1
	419085	species	*Pineus cembrae*	Adelgidae	Hemiptera	1
	384469	species	*Aleurodaphis asteris*	Hormaphididae	Hemiptera	1
	345558	species	*Aphis jacobaeae*	Aphididae	Hemiptera	1
	33415	family	Nymphalidae	Nymphalidae	Lepidoptera	1
	299363	genus	*Paracymoriza*	Crambidae	Lepidoptera	1
	295286	species	*Tettigarcta crinita*	Tettigarctidae	Hemiptera	1
	2704477	species	*Yamatocallis sauteri*	Aphididae	Hemiptera	1
	1689922	species	*Cinara nr. confinis 3272*	Aphididae	Hemiptera	1
	1086141	species	*Ambulyx dohertyi*	Sphingidae	Lepidoptera	1
HB_3B3	384450	species	*Rhopalus parumpunctatus*	Hormaphididae	Hemiptera	1376
	33386	tribe	Macrosiphini	Aphididae	Hemiptera	1103
	506608	species	*Cinara cedri*	Aphididae	Hemiptera	1091
	506607	species	*Cinara pilicornis*	Aphididae	Hemiptera	1064
	30161	family	Hormaphididae	Hormaphididae	Hemiptera	713
	464929	subgenus	*Aphis*	Aphididae	Hemiptera	665
	13164	species	*Myzus persicae*	Aphididae	Hemiptera	510
	935269	species	*Therioaphis trifolii*	Aphididae	Hemiptera	290
	7029	species	*Acyrthosiphon pisum*	Aphididae	Hemiptera	281
	12998	genus	*Chaitophorus*	Aphididae	Hemiptera	278
	1276923	species	*Hyalopterus amygdali*	Aphididae	Hemiptera	267
	36997	species	*Ceratoglyphina styracicola*	Hormaphididae	Hemiptera	187
	97051	genus	*Cinara*	Aphididae	Hemiptera	179
	80764	genus	*Aphis*	Aphididae	Hemiptera	154
	527648	species	*Drepanosiphum platanoidis*	Aphididae	Hemiptera	154
	230853	species	*Cinara wahtolca*	Aphididae	Hemiptera	144
	987866	species	*Agrochola circellaris*	Noctuidae	Hemiptera	132
	1037414	family	Hepialidae	Hepialidae	Lepidoptera	124
	13262	species	*Schizaphis graminum*	Aphididae	Hemiptera	123
	1421473	species	*Cinara pectinatae*	Aphididae	Hemiptera	102
	27482	family	Aphididae	Aphididae	Hemiptera	73
	1667255	species	*Macropodaphis sp. YW-2015*	Aphididae	Hemiptera	47
	44664	species	*Sitobion avenae*	Aphididae	Hemiptera	43
	2768670	species	*Paurocephala sauteri*	Liviidae	Hemiptera	37
	988118	species	*Gortyna flavago*	Noctuidae	Lepidoptera	31
	1689206	species	*Cinara todocola*	Aphididae	Hemiptera	20
	384387	species	*Cinara formosana*	Aphididae	Hemiptera	16
	312888	species	*Hyalopterus pruni*	Aphididae	Hemiptera	9
	1086167	species	*Ambulyx substrigilis*	Sphingidae	Lepidoptera	8
	384469	species	*Aleurodaphis asteris*	Hormaphididae	Hemiptera	7
	405028	species	*Issoria eugenia*	Nymphalidae	Lepidoptera	6
	133076	subfamily	Aphidinae	Aphididae	Hemiptera	6
	2778780	species	*Hamamelistes sp. NZMC aphid 37937*	Hormaphididae	Hemiptera	5
	685722	species	*Aulacorthum cirsicola*	Aphididae	Hemiptera	4
	345571	genus	*Melanaphis*	Aphididae	Hemiptera	3
	97069	species	*Maculolachnus submacula*	Aphididae	Hemiptera	2

	93883	species	*Tineola bisselliella*	Tineidae	Lepidoptera	2
	523178	tribe	Ambulycini	Sphingidae	Lepidoptera	2
	1071973	species	*Pterocomma rufipes*	Aphididae	Hemiptera	2
	96555	species	*Trama troglodytes*	Aphididae	Hemiptera	1
	95182	subfamily	Amphipyrinae	Noctuidae	Lepidoptera	1
	910641	species	*Brachycaudus pilosus*	Aphididae	Hemiptera	1
	876379	species	*Cethosia biblis*	Nymphalidae	Lepidoptera	1
	866753	species	*Aulacorthum corydalicola*	Aphididae	Hemiptera	1
	7137	species	*Galleria mellonella*	Pyralidae	Lepidoptera	1
	512393	species	*Ampelophaga rubiginosa*	Sphingidae	Lepidoptera	1
	384386	species	*Cavariella salicicola*	Aphididae	Hemiptera	1
	357502	species	*Adelges tsugae*	Adelgidae	Hemiptera	1
	2572971	subfamily	Greenideinae	Aphididae	Hemiptera	1
	2491959	species	*Acrapex curvata*	Noctuidae	Lepidoptera	1
	1086141	species	*Ambulyx dohertyi*	Sphingidae	Lepidoptera	1
	1078270	species	*Ceratovacuna silvestrii*	Hormaphididae	Hemiptera	1
	100479	species	*Aphis nerii*	Aphididae	Hemiptera	1
HT_1	202396	species	*Rhopalosiphum padi complex*	Aphididae	Hemiptera	2192
	345571	genus	*Melanaphis*	Aphididae	Hemiptera	1197
	80764	genus	*Aphis*	Aphididae	Hemiptera	783
	27482	family	Aphididae	Aphididae	Hemiptera	686
	542820	species	*Chromaphis juglandicola*	Aphididae	Hemiptera	684
	2020618	subspecies	*Chaitophorus saliapterus quinquemaculatus*	Aphididae	Hemiptera	677
	464929	subgenus	*Aphis*	Aphididae	Hemiptera	656
	13262	species	*Schizaphis graminum*	Aphididae	Hemiptera	370
	12998	genus	*Chaitophorus*	Aphididae	Hemiptera	363
	224525	genus	*Brachycaudus*	Aphididae	Hemiptera	247
	7029	species	*Acyrthosiphon pisum*	Aphididae	Hemiptera	177
	1421473	species	*Cinara pectinatae*	Aphididae	Hemiptera	130
	464946	genus	*Aphis*	Aphididae	Hemiptera	122
	58824	species	*Plodia interpunctella*	Pyralidae	Lepidoptera	54
	271217	species	*Mythimna separata*	Noctuidae	Lepidoptera	31
	312888	species	*Hyalopterus pruni*	Aphididae	Hemiptera	30
	384387	species	*Cinara formosana*	Aphididae	Hemiptera	27
	1417364	species	*Athyma kasa*	Nymphalidae	Lepidoptera	19
	334015	species	*Coreana raphaelis*	Lycaenidae	Lepidoptera	13
	33386	tribe	Macrosiphini	Aphididae	Hemiptera	11
	351416	genus	*Athyma*	Nymphalidae	Lepidoptera	10
	384385	species	*Macrosiphoniella yomogifoliae*	Aphididae	Hemiptera	8
	915061	species	*Uroleucon leonardi*	Aphididae	Hemiptera	7
	876379	species	*Cethosia biblis*	Nymphalidae	Lepidoptera	6
	87309	genus	*Uroleucon*	Aphididae	Hemiptera	5
	935269	species	*Therioaphis trifolii*	Aphididae	Hemiptera	4
	511022	species	*Lipaphis pseudobrassicae*	Aphididae	Hemiptera	4
	30161	family	Hormaphididae	Hormaphididae	Hemiptera	4
	1350445	species	*Dermaphis crematogastri*	Hormaphididae	Hemiptera	4
	1049572	species	*Mollitrichosiphum tenuicorpus*	Aphididae	Hemiptera	4
	51655	species	*Plutella xylostella*	Plutellidae	Lepidoptera	3
	702717	species	*Tuta absoluta*	Gelechiidae	Lepidoptera	2
	486037	species	*Anuraphis pyrilaseri*	Aphididae	Hemiptera	2

	486035	species	*Macrosiphum sp. C1774*	Aphididae	Hemiptera	2
	40932	species	*Rhopalosiphum padi*	Aphididae	Hemiptera	2
	396680	species	*Tecia solanivora*	Gelechiidae	Lepidoptera	2
	384469	species	*Aleurodaphis asteris*	Hormaphididae	Hemiptera	1
	384450	species	*Rhopalus parumpunctatus*	Hormaphididae	Hemiptera	1
	33415	family	Nymphalidae	Nymphalidae	Lepidoptera	1
	299363	genus	*Paracymoriza*	Crambidae	Lepidoptera	1
	2704473	species	*Macropodaphis paradoxa*	Aphididae	Hemiptera	1
	224527	species	*Aphis spiraecola*	Aphididae	Hemiptera	1
	2082423	species	*Ourapteryx adonidaria*	Geometridae	Lepidoptera	1
	179822	species	*Chaitophorus capreae*	Aphididae	Hemiptera	1
	133076	subfamily	Aphidinae	Aphididae	Hemiptera	1
	1248151	species	*Lethe marginalis*	Nymphalidae	Lepidoptera	1
	1244005	species	*Cinara brevisaeta*	Aphididae	Hemiptera	1
	1209556	species	*Nomophila noctuella*	Crambidae	Lepidoptera	1
HT_3	464929	subgenus	*Aphis*	Aphididae	Hemiptera	33788
	13164	species	*Myzus persicae*	Aphididae	Hemiptera	8793
	80764	genus	*Aphis*	Aphididae	Hemiptera	1005
	7029	species	*Acyrthosiphon pisum*	Aphididae	Hemiptera	632
	935269	species	*Therioaphis trifolii*	Aphididae	Hemiptera	524
	133076	subfamily	Aphidinae	Aphididae	Hemiptera	361
	51655	species	*Plutella xylostella*	Plutellidae	Lepidoptera	75
	80765	species	*Aphis gossypii*	Aphididae	Hemiptera	65
	27482	family	Aphididae	Aphididae	Hemiptera	64
	910915	species	*Aphidura bozhkoae*	Aphididae	Hemiptera	63
	511022	species	*Lipaphis pseudobrassicae*	Aphididae	Hemiptera	36
	400569	species	*Aphis nasturtii*	Aphididae	Hemiptera	29
	44664	species	*Sitobion avenae*	Aphididae	Hemiptera	22
	13262	species	*Schizaphis graminum*	Aphididae	Hemiptera	21
	33387	tribe	Aphidini	Aphididae	Hemiptera	8
	224525	genus	Brachycaudus	Aphididae	Hemiptera	5
	33386	tribe	Macrosiphini	Aphididae	Hemiptera	4
	2027655	species	*Sipha burakowskii*	Aphididae	Hemiptera	3
	1904465	species	*Hybothoracaphis laevigata*	Aphididae	Hemiptera	3
	168631	species	*Chilo suppressalis*	Crambidae	Lepidoptera	3
	511041	species	*Aphis sanguisorbicola*	Aphididae	Hemiptera	2
	511038	species	*Aphis kurosawai*	Aphididae	Hemiptera	2
	345567	species	*Aphis ulmariae*	Aphididae	Hemiptera	2
	464930	subgenus	*Bursaphis*	Aphididae	Hemiptera	1
	384362	species	*Kurisakia onigurumii*	Thelaxidae	Hemiptera	1
	345558	species	*Aphis jacobaeae*	Aphididae	Hemiptera	1
	345556	species	*Aphis idaei*	Aphididae	Hemiptera	1
	224527	species	*Aphis spiraecola*	Aphididae	Hemiptera	1
	2027656	species	*Trichaitophorus ginnalarus*	Aphididae	Hemiptera	1
	1462193	species	*Sumatraphis tubercaudatus*	Aphididae	Hemiptera	1
HT_13	935269	species	*Therioaphis trifolii*	Aphididae	Hemiptera	15445
	506608	species	*Cinara cedri*	Aphididae	Hemiptera	10746
	97051	genus	*Cinara*	Aphididae	Hemiptera	2073
	464929	subgenus	*Aphis*	Aphididae	Hemiptera	1534
	7029	species	*Acyrthosiphon pisum*	Aphididae	Hemiptera	1018

	27482	family	Aphididae	Aphididae	Hemiptera	984
	13262	species	*Schizaphis graminum*	Aphididae	Hemiptera	570
	464926	species	*Aphis aurantii*	Aphididae	Hemiptera	288
	69196	species	*Brevicoryne brassicae*	Aphididae	Hemiptera	268
	384387	species	*Cinara formosana*	Aphididae	Hemiptera	242
	80764	genus	*Aphis*	Aphididae	Hemiptera	226
	987454	species	*Anarta trifolii*	Noctuidae	Lepidoptera	46
	511024	species	*Schizaphis scirpi*	Aphididae	Hemiptera	46
	12998	genus	*Chaitophorus*	Aphididae	Hemiptera	31
	13164	species	*Myzus persicae*	Aphididae	Hemiptera	25
	236772	genus	*Busseola*	Noctuidae	Lepidoptera	23
	506605	species	*Cinara maghrebica*	Aphididae	Hemiptera	21
	143948	species	*Diuraphis noxia*	Aphididae	Hemiptera	17
	254363	species	*Autographa gamma*	Noctuidae	Lepidoptera	12
	270466	species	*Limenitis camilla*	Nymphalidae	Lepidoptera	7
	527648	species	*Drepanosiphum platanoidis*	Aphididae	Hemiptera	6
	97078	species	*Stomaphis yanonis*	Aphididae	Hemiptera	5
	133076	subfamily	Aphidinae	Aphididae	Hemiptera	5
	384385	species	*Macrosiphoniella yomogifoliae*	Aphididae	Hemiptera	3
	47767	species	*Agrotis segetum*	Noctuidae	Lepidoptera	2
	33387	tribe	Aphidini	Aphididae	Hemiptera	2
	198323	species	*Cinara tujafilina*	Aphididae	Hemiptera	2
	168631	species	*Chilo suppressalis*	Crambidae	Lepidoptera	2
	99934	species	*Lachnus roboris*	Aphididae	Hemiptera	1
	99933	species	*Lachnus tropicalis*	Aphididae	Hemiptera	1
	96541	subfamily	Lachninae	Aphididae	Hemiptera	1
	384465	species	*Ceratovacuna panici*	Hormaphididae	Hemiptera	1
	33386	tribe	Macrosiphini	Aphididae	Hemiptera	1
	2137626	species	*Acrapex mondogeneta*	Noctuidae	Lepidoptera	1
	1078815	species	*Spilarctia subcarnea*	Erebidae	Lepidoptera	1
HT_19	506608	species	*Cinara cedri*	Aphididae	Hemiptera	53377
	464929	subgenus	*Aphis*	Aphididae	Hemiptera	2547
	27482	family	*Aphididae*	Aphididae	Hemiptera	1225
	419076	genus	*Pineus*	Adelgidae	Hemiptera	1160
	80764	genus	*Aphis*	Aphididae	Hemiptera	942
	7029	species	*Acyrthosiphon pisum*	Aphididae	Hemiptera	820
	935269	species	*Therioaphis trifolii*	Aphididae	Hemiptera	515
	143948	species	*Diuraphis noxia*	Aphididae	Hemiptera	484
	12998	genus	*Chaitophorus*	Aphididae	Hemiptera	426
	511022	species	*Lipaphis pseudobrassicae*	Aphididae	Hemiptera	273
	44664	species	*Sitobion avenae*	Aphididae	Hemiptera	270
	987995	species	*Noctua janthe*	Noctuidae	Lepidoptera	213
	915061	species	*Uroleucon leonardi*	Aphididae	Hemiptera	201
	97051	genus	*Cinara*	Aphididae	Hemiptera	131
	2020618	subspecies	*Chaitophorus saliapterus quinquemaculatus*	Aphididae	Hemiptera	129
	1276923	species	*Hyalopterus amygdali*	Aphididae	Hemiptera	119
	33386	tribe	Macrosiphini	Aphididae	Hemiptera	118
	875884	species	*Phlogophora meticulosa*	Noctuidae	Lepidoptera	117
	13262	species	*Schizaphis graminum*	Aphididae	Hemiptera	115
	464926	species	*Aphis aurantii*	Aphididae	Hemiptera	106

	224525	genus	*Brachycaudus*	Aphididae	Hemiptera	99
	157393	species	*Lamproptera meges*	Papilionidae	Lepidoptera	53
	1421473	species	*Cinara pectinatae*	Aphididae	Hemiptera	47
	133076	subfamily	Aphidinae	Aphididae	Hemiptera	33
	400569	species	*Aphis nasturtii*	Aphididae	Hemiptera	25
	2713608	species	*Hyalopterus arundiniformis*	Aphididae	Hemiptera	22
	312888	species	*Hyalopterus pruni*	Aphididae	Hemiptera	17
	796230	species	*Greenidea kuwanai*	Aphididae	Hemiptera	6
	384387	species	*Cinara formosana*	Aphididae	Hemiptera	6
	1078272	species	*Schizolachnus orientalis*	Aphididae	Hemiptera	6
	214277	species	*Noctua pronuba*	Noctuidae	Lepidoptera	3
	13164	species	*Myzus persicae*	Aphididae	Hemiptera	3
	1064595	species	*Schoutedenia ralumensis*	Aphididae	Hemiptera	3
	95179	subfamily	Noctuinae	Noctuidae	Lepidoptera	2
	80765	species	*Aphis gossypii*	Aphididae	Hemiptera	2
	469903	species	*Megoura lespedezae*	Aphididae	Hemiptera	2
	42282	subfamily	Satyrinae	Nymphalidae	Lepidoptera	2
	384385	species	*Macrosiphoniella yomogifoliae*	Aphididae	Hemiptera	2
	97078	species	*Stomaphis yanonis*	Aphididae	Hemiptera	1
	910915	species	*Aphidura bozhkoae*	Aphididae	Hemiptera	1
	87322	species	*Uroleucon sonchi*	Aphididae	Hemiptera	1
	87309	genus	*Uroleucon*	Aphididae	Hemiptera	1
	722268	subspecies	*Morpho deidamia granadensis*	Nymphalidae	Lepidoptera	1
	527648	species	*Drepanosiphum platanoidis*	Aphididae	Hemiptera	1
	524428	species	*Macroglossum stellatarum*	Sphingidae	Lepidoptera	1
	47767	species	*Agrotis segetum*	Noctuidae	Lepidoptera	1
	253253	species	*Rhopalosiphum nymphaeae*	Aphididae	Hemiptera	1
	168631	species	*Chilo suppressalis*	Crambidae	Lepidoptera	1
	1635117	species	*Conicofrontia diamesa*	Noctuidae	Lepidoptera	1
	1350441	species	*Nipponaphis sp. JC-2013*	Hormaphididae	Hemiptera	1
	1244010	species	*Cinara laricis*	Aphididae	Hemiptera	1
	1078815	species	*Spilarctia subcarnea*	Erebidae	Lepidoptera	1
HT_28	1078272	species	*Schizolachnus orientalis*	Aphididae	Hemiptera	14210
	419076	genus	*Pineus*	Adelgidae	Hemiptera	1323
	2020618	subspecies	*Chaitophorus saliapterus quinquemaculatus*	Aphididae	Hemiptera	319
	96541	subfamily	*Lachninae*	Aphididae	Hemiptera	200
	1925539	species	*Cinara nr. spiculosa 3417*	Aphididae	Hemiptera	134
	506605	species	*Cinara maghrebica*	Aphididae	Hemiptera	124
	158624	species	*Propylea japonica*	Coccinellidae	Coleoptera	88
	488727	species	*Astegopteryx styracophila*	Hormaphididae	Hemiptera	65
	1049572	species	*Mollitrichosiphum tenuicorpus*	Aphididae	Hemiptera	59
	202396	species	*Rhopalosiphum padi complex*	Aphididae	Hemiptera	40
	27482	family	Aphididae	Aphididae	Hemiptera	37
	1134961	subspecies	*Morpho sulkowskyi calderoni*	Nymphalidae	Lepidoptera	34
	420590	species	*Dendrolimus superans*	Lasiocampidae	Lepidoptera	33
	99934	species	*Lachnus roboris*	Aphididae	Hemiptera	25
	1244020	species	*Eulachnus nigricola*	Aphididae	Hemiptera	17
	464929	subgenus	*Aphis*	Aphididae	Hemiptera	16
	542820	species	*Chromaphis juglandicola*	Aphididae	Hemiptera	13
	7029	species	*Acyrthosiphon pisum*	Aphididae	Hemiptera	11

97051	genus	*Cinara*	Aphididae	Hemiptera	7
97063	species	*Cinara pseudotaxifoliae*	Aphididae	Hemiptera	6
1244010	species	*Cinara laricis*	Aphididae	Hemiptera	6
1276923	species	*Hyalopterus amygdali*	Aphididae	Hemiptera	5
76195	species	*Papilio helenus*	Papilionidae	Lepidoptera	3
796230	species	*Greenidea kuwanai*	Aphididae	Hemiptera	2
13164	species	*Myzus persicae*	Aphididae	Hemiptera	2
400569	species	*Aphis nasturtii*	Aphididae	Hemiptera	1
133076	subfamily	Aphidinae	Aphididae	Hemiptera	1
13122	genus	*Lymantria*	Erebidae	Lepidoptera	1
1088304	species	*Theretra oldenlandiae*	Sphingidae	Lepidoptera	1
1078267	species	*Mindarus keteleerifoliae*	Mindaridae	Hemiptera	1
1071973	species	*Pterocomma rufipes*	Aphididae	Hemiptera	1

Table S4. Average number of families and species detected in the honey samples from the three regions investigated and results of the t-test statistics in the comparison between the number of families and species detected by the COI and CYTB metabarcodes for each region investigated.

**Region**	**COI**		**CYTB**		***P* value**	
	**Average no. of families**	**Average no. of species**	**Average no. of families**	**Average no. of species**	**No. of families**	**No. of species**
Calabria	2.83	11.58	7.17	41.00	3.82E-04	5.00E-06
Emilia-Romagna	6.13	11.38	9.25	30.00	0.09	2.21E-03
Türkiye	6.00	12.40	7.60	33.60	0.30	4.81E-03
Total	4.52	11.68	7.92	36.00	1.90E-04	5.00E-10

Table S5. Results of t-test statistics within marker between the number of families and species detected for each region investigated.

**Regions**	**COI**		**CYTB**	
	***P* families**	***P* species**	***P* families**	***P* species**
**Calabria vs Emilia-Romagna**	0.003	0.892	0.254	0.064
**Calabria vs Türkiye**	0.014	0.629	0.771	0.207
**Emilia-Romagna vs Türkiye**	0.913	0.474	0.405	0.540

Table S6. Number of reads assigned to different COI mitochondrial haplotypes (mitotypes) for *Metcalfa pruinosa*, *Thelaxes suberi*, *Cinara cedri* and *Aphis gossypii* identified in the analysed honey samples. In blue, mitotypes identified in this study.

COI Mitotypes	ID NCBI Nucleotide	HC_1	HC_2	HC_3	HC_4	HC_5	HC_6	HC_7	HC_8	HC_9	HC_10	HC_11	HC_12	HB_1A1	HB_1A2	HB_1C1	HB_2A2	HB_2B2	HB_2C2	HB_3A3	HB_3B3	HT_1	HT_3	HT_13	HT_19	HT_28
		N. reads	N. reads	N. reads	N. reads	N. reads	N. reads	N. reads	N. reads	N. reads	N. reads	N. reads	N. reads	N. reads	N. reads	N. reads	N. reads	N. reads	N. reads	N. reads	N. reads	N. reads	N. reads	N. reads	N. reads	N. reads
Metcalfa pruinosa	**total**	**0**	**0**	**0**	**0**	**0**	**0**	**0**	**0**	**0**	**0**	**0**	**0**	**6447**	**46719**	**71**	**9813**	**5672**	**26056**	**38660**	**29633**	**0**	**0**	**0**	**0**	**0**
MP_Hap1	KJ412927.1	0	0	0	0	0	0	0	0	0	0	0	0	0	0	0	0	0	0	0	0	0	0	0	0	0
MP_Hap2	KT382723.1	0	0	0	0	0	0	0	0	0	0	0	0	0	83	0	0	0	0	0	0	0	0	0	0	0
MP_Hap3	MK302865.1	0	0	0	0	0	0	0	0	0	0	0	0	6447	46534	69	9800	5672	25776	38592	29631	0	0	0	0	0
MP_Hap4	KR346216.1	0	0	0	0	0	0	0	0	0	0	0	0	0	0	0	0	0	0	0	0	0	0	0	0	0
MP_Hap5	KJ412951.1	0	0	0	0	0	0	0	0	0	0	0	0	0	75	1	0	0	210	33	0	0	0	0	0	0
MP_Hap6	KR033745.1	0	0	0	0	0	0	0	0	0	0	0	0	0	0	0	0	0	0	0	0	0	0	0	0	0
MP_Hap7	KJ412936.1	0	0	0	0	0	0	0	0	0	0	0	0	0	0	0	0	0	0	0	0	0	0	0	0	0
MP_Hap8	KR036383.1	0	0	0	0	0	0	0	0	0	0	0	0	0	0	0	0	0	0	0	0	0	0	0	0	0
MP_Hap9	KJ412929.1	0	0	0	0	0	0	0	0	0	0	0	0	0	0	0	0	0	0	0	0	0	0	0	0	0
MP_Hap10	KJ412925.1	0	0	0	0	0	0	0	0	0	0	0	0	0	24	0	0	0	0	0	0	0	0	0	0	0
MP_Hap11	KT382709.1	0	0	0	0	0	0	0	0	0	0	0	0	0	0	0	0	0	0	0	0	0	0	0	0	0
MP_Hap12	KJ412947.1	0	0	0	0	0	0	0	0	0	0	0	0	0	3	1	13	0	70	35	2	0	0	0	0	0
Thelaxes suberi	**total**	**0**	**0**	**106**	**4857**	**0**	**0**	**0**	**0**	**0**	**0**	**0**	**0**	**0**	**0**	**0**	**1780**	**31**	**0**	**76**	**0**	**0**	**0**	**501**	**0**	**66136**
TS_Hap1	KF639660.1	0	0	0	0	0	0	0	0	0	0	0	0	0	0	0	6	0	0	0	0	0	0	390	0	46
TS_Hap2	MW052859.1	0	0	0	0	0	0	0	0	0	0	0	0	0	0	0	10	0	0	23	0	0	0	111	0	360
TS_Hap3	KR029854.1	0	0	106	4857	0	0	0	0	0	0	0	0	0	0	0	1764	31	0	53	0	0	0	0	0	65730
Cinara cedri	**total**	**0**	**0**	**32**	**137**	**242**	**0**	**0**	**0**	**0**	**66**	**0**	**0**	**300**	**0**	**0**	**0**	**3023**	**4488**	**0**	**130**	**0**	**0**	**23591**	**52626**	**0**
CC_Hap1	KJ433268.1	0	0	0	0	0	0	0	0	0	0	0	0	0	0	0	0	1794	2418	0	129	0	0	23591	52626	0
CC_Hap2	LT600418.1	0	0	32	137	242	0	0	0	0	66	0	0	300	0	0	0	1229	2070	0	1	0	0	0	0	0
Aphis gossypii	**total**	**236**	**616**	**95**	**478**	**463**	**321**	**584**	**495**	**196**	**440**	**467**	**460**	**2**	**0**	**0**	**0**	**0**	**0**	**1**	**0**	**1**	**26**	**0**	**0**	**0**
AG_Hap1	KR032414	0	0	0	0	0	0	0	0	0	0	0	0	0	0	0	0	0	0	0	0	0	0	0	0	0
AG_Hap2	MH821159	0	0	1	2	0	0	2	0	0	4	0	1	0	0	0	0	0	0	0	0	0	1	0	0	0
AG_Hap3	JQ690333	196	485	36	233	450	5	31	213	11	243	115	183	1	0	0	0	0	0	0	0	1	11	0	0	0
AG_Hap4	MN320355	0	0	0	0	0	0	0	0	0	0	0	0	0	0	0	0	0	0	0	0	0	0	0	0	0
AG_Hap5	JQ916116	0	0	0	0	0	0	0	0	0	0	0	0	0	0	0	0	0	0	0	0	0	0	0	0	0
AG_Hap6	KP152429	0	0	0	0	0	0	0	0	0	0	0	0	0	0	0	0	0	0	0	0	0	0	0	0	0

AG_Hap7	AY227082	0	0	0	0	0	0	0	0	0	0	0	0	0	0	0	0	0	0	0	0	0	0	0	0	0
AG_Hap8	MN319883	0	0	0	0	0	0	0	0	0	0	0	0	0	0	0	0	0	0	0	0	0	0	0	0	0
AG_Hap9	MT461595	0	0	0	0	0	0	0	0	0	0	0	0	0	0	0	0	0	0	0	0	0	0	0	0	0
AG_Hap10	KY679409	0	0	0	0	0	0	0	0	0	0	0	0	0	0	0	0	0	0	0	0	0	0	0	0	0
AG_Hap11	MN319919	0	0	0	0	0	0	0	0	0	0	0	0	0	0	0	0	0	0	0	0	0	0	0	0	0
AG_Hap12	MN319919	0	0	0	0	0	0	0	0	0	0	0	0	0	0	0	0	0	0	0	0	0	0	0	0	0
AG_Hap13	JQ916093	0	0	0	0	0	0	0	0	0	0	0	0	0	0	0	0	0	0	0	0	0	0	0	0	0
AG_Hap14	MH821226	0	0	0	1	1	0	1	0	0	0	0	1	0	0	0	0	0	0	0	0	0	0	0	0	0
AG_Hap15	JQ690329	0	0	0	0	0	0	0	0	0	0	0	0	0	0	0	0	0	0	0	0	0	0	0	0	0
AG_Hap16	KP152430	1	0	0	0	0	0	41	0	0	1	0	0	0	0	0	0	0	0	1	0	0	0	0	0	0
AG_Hap17	GU559858	0	0	0	0	0	0	0	0	0	0	0	0	0	0	0	0	0	0	0	0	0	0	0	0	0
AG_Hap18	JX051429	0	0	0	1	0	0	0	0	0	0	0	0	0	0	0	0	0	0	0	0	0	1	0	0	0
AG_Hap19	KP152451	0	0	0	0	0	0	0	0	0	0	0	0	0	0	0	0	0	0	0	0	0	1	0	0	0
AG_Hap20	KP152444	0	0	0	0	0	0	0	1	1	0	0	0	0	0	0	0	0	0	0	0	0	0	0	0	0
AG_Hap21	MH821192	0	0	0	0	0	0	0	0	0	0	0	0	0	0	0	0	0	0	0	0	0	0	0	0	0
AG_Hap22	HQ215543	0	0	0	1	0	0	1	0	0	0	1	0	0	0	0	0	0	0	0	0	0	0	0	0	0
AG_Hap23	MN320145	0	0	0	0	0	0	0	0	0	0	0	0	0	0	0	0	0	0	0	0	0	0	0	0	0
AG_Hap24	JQ690335	37	124	56	229	5	314	502	278	180	185	349	271	0	0	0	0	0	0	0	0	0	7	0	0	0
AG_Hap25	KF446152	0	0	0	0	0	0	0	0	0	0	0	0	0	0	0	0	0	0	0	0	0	0	0	0	0
AG_Hap26	KP152479	0	0	1	1	1	0	1	0	1	0	0	0	0	0	0	0	0	0	0	0	0	0	0	0	0
AG_Hap27	KM115479	1	4	1	5	1	0	2	0	2	6	0	2	1	0	0	0	0	0	0	0	0	2	0	0	0
AG_Hap28	MH821188	0	1	0	0	0	0	0	0	0	1	0	0	0	0	0	0	0	0	0	0	0	1	0	0	0
AG_Hap29	FJ965686	0	0	0	0	0	0	0	0	0	0	0	0	0	0	0	0	0	0	0	0	0	0	0	0	0
AG_Hap30	JX966028	0	0	0	0	0	0	0	0	0	0	0	0	0	0	0	0	0	0	0	0	0	0	0	0	0
AG_Hap31	KM268006	0	0	0	1	1	0	0	0	0	0	0	1	0	0	0	0	0	0	0	0	0	0	0	0	0
AG_Hap32	JQ916104	0	0	0	0	0	0	0	0	0	0	0	0	0	0	0	0	0	0	0	0	0	0	0	0	0
AG_Hap33	MN319812	0	0	0	0	0	0	0	0	0	0	0	0	0	0	0	0	0	0	0	0	0	0	0	0	0
AG_Hap34	JQ067097	0	1	0	2	3	1	2	1	1	0	1	1	0	0	0	0	0	0	0	0	0	2	0	0	0
AG_Hap35	MT461596	0	0	0	0	0	0	0	0	0	0	0	0	0	0	0	0	0	0	0	0	0	0	0	0	0
AG_Hap36	AB506729	1	1	0	2	1	1	1	2	0	0	1	0	0	0	0	0	0	0	0	0	0	0	0	0	0

Table S7. Number of reads assigned to CYTB mitochondrial haplotypes (mitotypes) for *Cinara cedri* and *Myzus persicae* identified in the analysed honey samples. In blue, mitotypes identified in this study.

**CYTB Mitotypes**		**HC_1**	**HC_2**	**HC_3**	**HC_4**	**HC_5**	**HC_6**	**HC_7**	**HC_8**	**HC_9**	**HC_10**	**HC_11**	**HC_12**	**HB_1A1**	**HB_1A2**	**HB_1C1**	**HB_2A2**	**HB_2B2**	**HB_2C2**	**HB_3A3**	**HB_3B3**	**HT_1**	**HT_3**	**HT_13**	**HT_19**	**HT_28**
		N. reads	N. reads	N. reads	N. reads	N. reads	N. reads	N. reads	N. reads	N. reads	N. reads	N. reads	N. reads	N. reads	N. reads	N. reads	N. reads	N. reads	N. reads	N. reads	N. reads	N. reads	N. reads	N. reads	N. reads	N. reads
Cinara cedri	**total**	**2**	**0**	**15**	**21**	**6**	**0**	**0**	**0**	**0**	**0**	**0**	**0**	**0**	**0**	**3**	**40**	**398**	**1017**	**0**	**770**	**0**	**0**	**8259**	**41541**	**0**
CC_CYTB_Hap1	KF694101.1	2	0	15	21	6	0	0	0	0	0	0	0	0	0	3	40	398	1017	0	770	0	0	8259	41541	0
CC_CYTB_Hap2	KY064315.1	0	0	0	0	0	0	0	0	0	0	0	0	0	0	0	0	0	0	0	0	0	0	0	0	0
Myzus persicae	**total**	**9602**	**690**	**759**	**3555**	**5543**	**2917**	**3543**	**3916**	**2014**	**674**	**3235**	**836**	**5499**	**1706**	**1666**	**0**	**88**	**0**	**1658**	**305**	**0**	**7673**	**24**	**3**	**0**
My_CYTB_Hap1	KU236024.1	9602	690	759	3555	5543	2917	3543	3916	2014	674	3235	836	5499	1706	1666	0	88	0	1658	305	0	7673	24	3	0
My_CYTB_Hap2	KU877171.1	0	0	0	0	0	0	0	0	0	0	0	0	0	0	0	0	0	0	0	0	0	0	0	0	0
My_CYTB_Hap3	KP722575.1	0	0	0	0	0	0	0	0	0	0	0	0	0	0	0	0	0	0	0	0	0	0	0	0	0

Table S8. Correspondence between *Metcalfa pruinosa* mitotypes identified in our study with those reported in Utzeri et al. [18], Lee et al. [43] and Park et al. [42], as well as those deduced from GenBank entries.

Haplotypes from this study	Haplotype from Utzeri et al. [18]	Haplotypes from Lee et al. [43]	Haplotypes from Park et al. [42]	GenBank entry
MP_Hap1	Hap4	MPH04	H10	KJ412927.1
MP_Hap2	Hap2	MPH15	H11, H13	KT382723.1
MP_Hap3	Hap6	MPH01	H1, H2	MK302865.1
MP_Hap4	Hap1	MPH02, MPH03, MPH08, MPH09, MPH10, MPH11, MPH13, MPH16. MPH17, MPH18, MPH19, MPH20	H3, H8, H9, H15, H16, H18, H19	KR346216.1
MP_Hap5	Hap12		H4	KJ412951.1
MP_Hap6	Hap7			KR033745.1
MP_Hap7	Hap9	MPH07	H17	KJ412936.1
MP_Hap8	Hap8			KR036383.1
MP_Hap9	Hap11	MPH05	H14	KJ412929.1
MP_Hap10	Hap10			KJ412925.1
MP_Hap11	Hap3	MPH14	H12	KT382709.1
MP_Hap12	Hap5	MPH06, MPH12	H6, H7	KJ412947.1

Supplementary Figures

Figure S1. Alignment of the CYTB sequence region amplified in this study with the corresponding gene region in aphid species representative of different subfamilies.

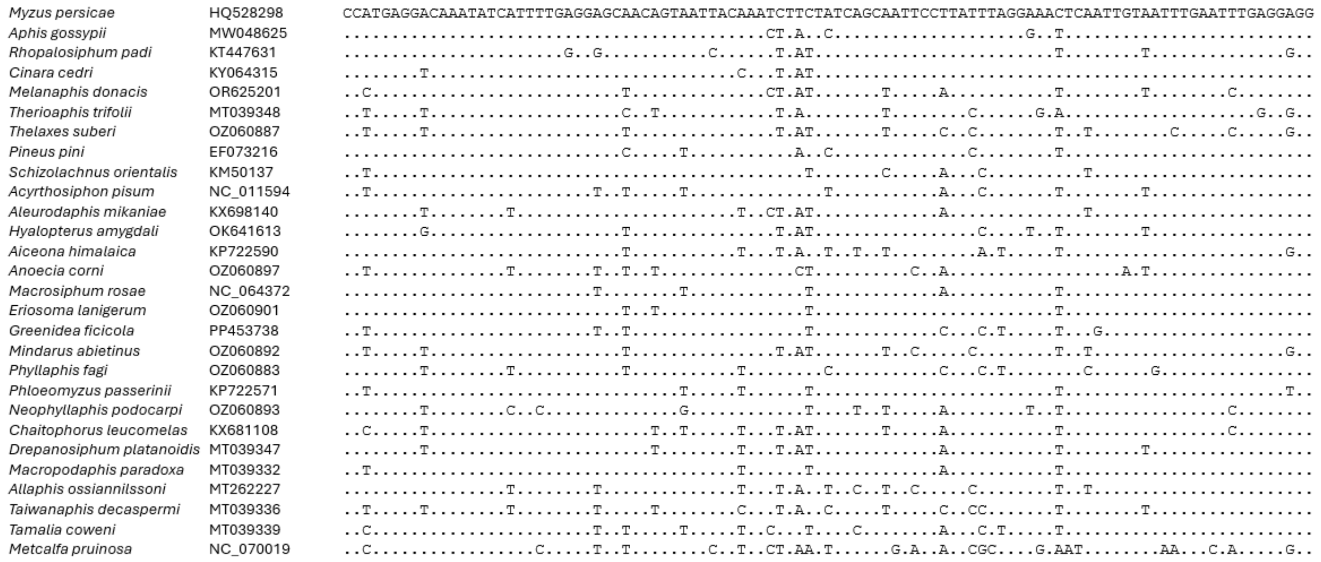

Figure S2. Sequence alignment of CYTB mitochondrial haplotypes found in *Cinara cedri* and *Myzus persicae*. Nucleotide positions identical with those of the first sequence are marked with a dot.

Cinara cedri

```
CC_CYTB_Hap1 AGGAGCTACAGTAATTACAAATTTATTGTCAGCTATCCCATATCTAGGTAATTCAATTGTTATTT
CC_CYTB_Hap2 ......A...........C........A.....A..T..T...T....A..C........A....
```

Myzus persicae

```
My_CYTB_Hap1 AGGAGCAACAGTAATTACAAATCTTCTATCAGCAATTCCTTATTTAGGAAACTCAATT
My_CYTB_Hap2 G..................................................T......
My_CYTB_Hap3 ...................................................T......
```
